# Lewis Acid-Catalyzed 2,3-Dihydrofuran Acetal Ring-Opening Benzannulations toward Functionalized 1-Hydroxycarbazoles

**DOI:** 10.3390/molecules27238344

**Published:** 2022-11-30

**Authors:** Shaoren Yuan, Gabriel Guerra Faura, Hailey E. Areheart, Natalie E. Peulen, Stefan France

**Affiliations:** 1 Georgia Institute of Technology, School of Chemistry and Biochemistry, Atlanta, GA 30332, USA; 2 Petit Institute for Bioengineering and Bioscience, Atlanta, GA 30332, USA

**Keywords:** benzannulation, carbazoles, dihydrofuran acetals, ring-opening cyclizations, synthetic methods

## Abstract

The development of a Lewis acid-catalyzed, intramolecular ring-opening benzannulation of 5-(indolyl)2,3-dihydrofuran acetals is described. The resulting 1-hydroxycarbazole-2-carboxylates are formed in up to 90% yield in 1 h. The dihydrofuran acetals are readily accessed from the reactions of enol ethers and α-diazo-β-indolyl-β-ketoesters. To highlight the method’s synthetic utility, a formal total synthesis of murrayafoline A, a bioactive carbazole-containing natural product, was undertaken.

## 1. Introduction

**Figure 1:**
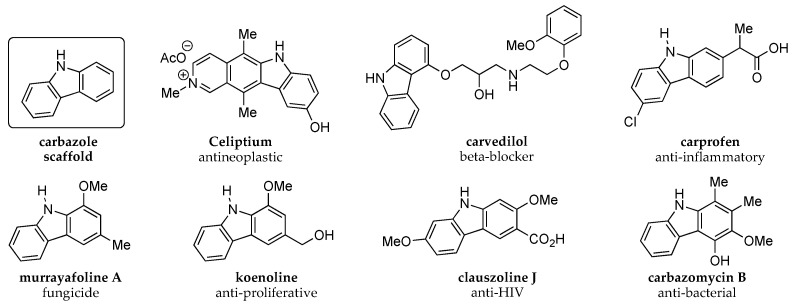
The Carbazole Scaffold and Representative Bioactive Derivatives.

The carbazole scaffold and its derivatives represent a privileged class of nitrogen-containing heteroaromatic structures often found in bioactive natural products and pharmaceutical drugs (Figure 1) [1,2,3,4,5,6,7]. For instance, Celiptium^®^ is a marketed drug utilized for metastatic breast cancer [8], while carvedilol is used treat high blood pressure and heart failure [9]. In another example, carprofen is an anti-flammatory drug prescribed in veterinary medicine [10]. Carbazoles are also frequently employed in the development of advanced materials due to their conjugated tricyclic structure, which provides a tunable π-extended system [11]. This feature has been exploited for optical and thermoelectronic applications such as fluorescent dyes for bioimaging and conductive polymers for transistors, light emitting diodes, biosensors, and photovoltaic devices [12,13,14].

**Figure 2:**
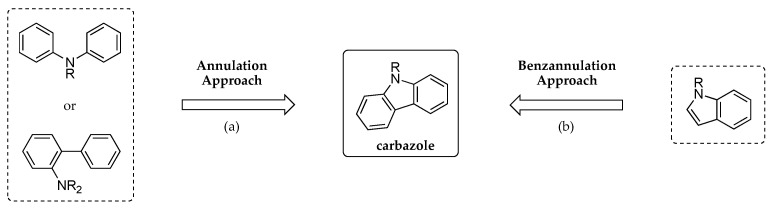
Standard approaches to the carbazole framework.

The synthesis of carbazoles generally follows two distinct approaches (Figure 2): (a) an annulation reaction to generate the central pyrrole ring or (b) a benzannulation reaction in which a benzene ring is appended on the five-member ring of an indole [15,16,17,18,19]. While several syntheses have been reported [20,21,22,23,24,25,26,27,28,29,30,31,32,33,34,35,36,37,38,39,40,41,42,43,44], the abundant presence of the carbazole core in bioactive molecules and photoelectronic materials makes them attractive targets for the development of new synthetic methodologies, often targeting new substitution patterns, modularity and mild reaction conditions.

**Figure 3:**
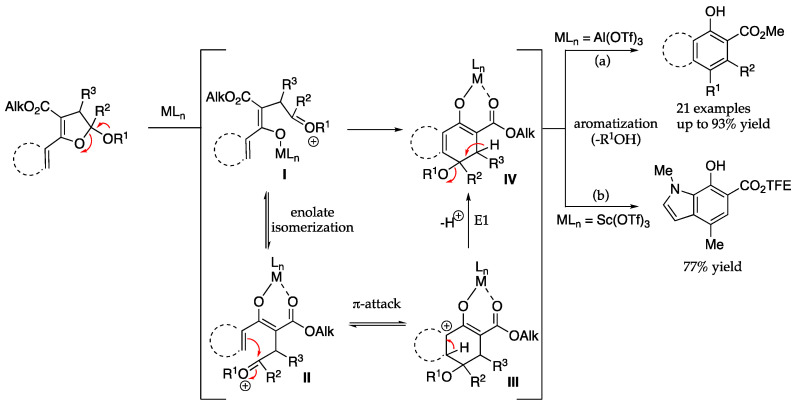
Dihydrofuran acetals as building blocks for benzannulation to generate (a) arenes and oxygen-/sulfur-containg heteroaromatics and (b) indoles [45,46].

Our lab previously established a Lewis acid-catalyzed approach to intramolecular benzannulation using (hetero)aryl-substituted 2,3-dihydrofuran acetals as precursors (Figure 3) [45,46]. Vicinal hydroxybenzoates are thus produced following dihydrofuran ring opening (via acetal hydrolysis), enolate isomerization, intramolecular π-attack (on the resulting oxonium **II**), and subsequent alcohol elimination. We demonstrated the versatility of this benzannulation approach using dihydrofurans substituted with arenes and oxygen- and sulfur-containing-heteroaromatics, resulting in the formation of naphthalenes, benzofurans, dibenzo[*b*,*d*]furans, phenanthrenes, and benzothiophenes in good to high yields (Figure 3a) [45]. More recently, we demonstrated that a (2-pyrrolyl)-substituted 2,3-dihydrofuran acetal was readily converted to the corresponding 7-hydroxyindole-6-carboxylate in 77% yield (Figure 3b)[46].

**Scheme 1:**
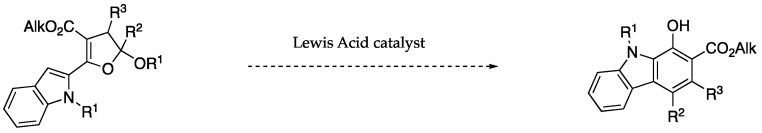
Proposed intramolecular ring-opening benzannulation approach to 1-hydroxycarbazoles.

Substituted 1-hydroxycarbazoles are an important class of bioactive carbazoles that have been the targets of synthetic efforts over the last 15 years [47,48]. Many of these efforts begin with commercial 1-hydroxycarbazole, requiring numerous steps for regioselective substituent installation [49]. Direct regioselective methods to 1-hydroxycarbazoles are relatively scarce in the literature. Thus, a need still exists for the efficient syntheses of these structural motifs. Encouraged by the successful example of indole formation from a dihydrofuran acetal [46], we herein discuss our efforts to develop efficient Lewis acid-catalyzed intramolecular ring-opening benzannulations of the corresponding indolyl-substituted dihydrofuran acetals to form 1-hydroxy-9*H*-carbazole-2-carboxylates (Scheme 1).

## 2. Results and Discussion

### 2.1. Synthesis of Dihydrofuran Acetals 3

**Scheme 2:**
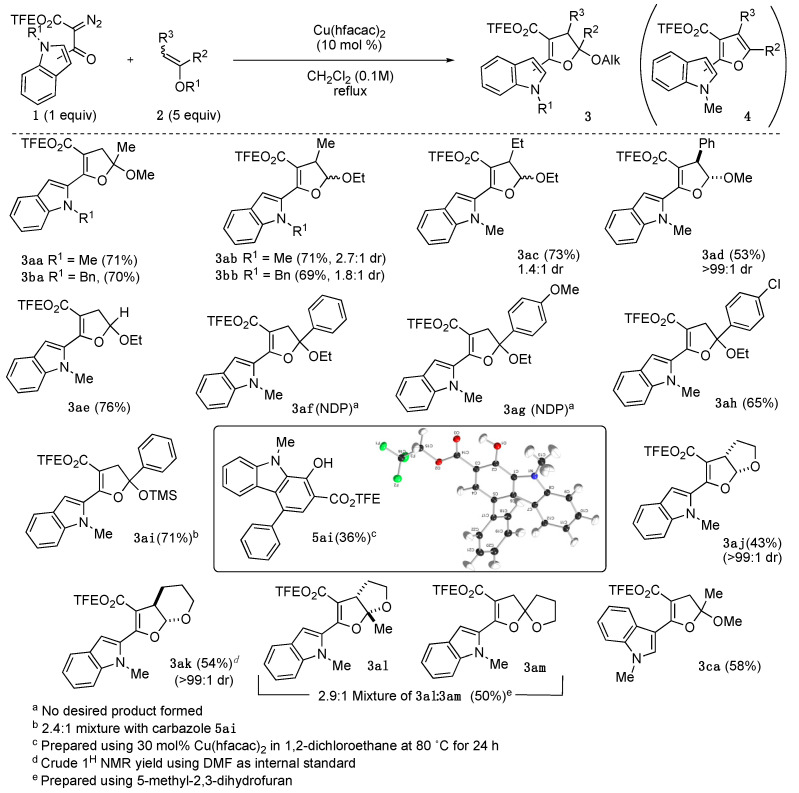
Synthesis of Dihydrofuran Acetals **3**.

Synthesis of dihydrofuran acetals **3** were accomplished through the Cu(hfacac)_2_-catalyzed decomposition of *N*-indolyl α-diazo-β-ketoesters **1** in the presence of enol ethers **2** (Scheme 2) [46]. In most cases, the dihydrofuran-forming reaction proceeded as expected with yields up to 76%. The outliers included the reactions with 1-aryl-substituted enol ethers **2f–2i**, dihydropyran **2k**, and 5-methyl-2,3-dihydrofuran **2l**.

Under the reaction conditions, no dihydrofuran products were detected with the 1-aryl-susbtituted ethyl enol ethers **2f** and **2g**. Instead, the corresponding furans (**4af** and **4ag**) were obtained. It is likely that the dihydrofuran serves as a short-lived intermediate which readily undergoes Cu-promoted EtOH elimination to provide the conjugated furan. In contrast, when a *p*-chloro group is placed on the phenyl ring, dihydrofuran **3ah** is readily formed in 65% yield. Employing the corresponding 1-aryl silyl enol ether **2i** in the reaction conditions, unexpectedly provided a 2.4:1 inseparable mixture of dihydrofuran **3ai** (50%) along with carbazole **5ai** (21%). After some minor attempts to design direct one-pot or tandem transformations, we determined that carbazole **5ai** could only be isolated in up to 36% yield when diazo **1a** and enol ether **2i** were treated with 20 mol% Cu(hfacac)_2_ in 1,2-dichloroethane at 70 °C for 24 h.

For tetrahydropyran **2k**, the desired dihydrofuran **3ak** was obtained as an inseparable mixture with unidentifiable material in a 56% ^1^H NMR yield. The crude mixture was carried forward. 5-Methyl-2,3-dihydrofuran **2l** underwent partial in situ alkene isomerization to form 2-methylene tetrahydrofuran **2m**. This isomerization resulted in the formation of a 2.9:1 mixture of fused-bicyclic dihydrofuran **3al** and spirocyclic dihydrofuran **3am** in 50% total yield.

### 2.2. Acid Screening for Ring-Opening Benzannulation

Dihydrofuran **3aa** was selected as the initial system to begin optimizing the ring-opening benzannulation (Table 1). Based on our previous work, we began by screening Lewis and Brønsted acids at 10 mol% catalyst loading in toluene at 70 °C. We confirmed that no reaction occurs in the absence of an acid catalyst (entry 1). Al(OTf)_3_, the best performing Lewis acid in our previous study, provided carbazole **5aa** in 79% yield (entry 2). Both Sc(OTf)_3_ and Ga(OTf)_3_ gave the carbazole with yields of 71% and 66%, respectively (entries 3 and 4). Divalent metals, Zn(OTf)_2_ and Mg(OTf)_2_, offered little or no product (entries 5 and 6). In contrast, Yb(OTf)_3_ generated carbazole **5aa** in 90% yield (entry 7). In(OTf)_3_ afforded 82% yield of carbazole (entry 8), while tetravalent Hf(OTf)_4_ gave the product in 79% yield (entry 9). To test the influence of any potential TfOH formed in the reaction, we ran a series of control reactions. First, YbCl_3_ and AlCl_3_ were employed in the reaction. YbCl_3_ gave low conversion with only 24% yield of **5aa** (entry 10). AlCl_3_ provided **5aa** in 80% yield (entry 11). With TfOH, **5aa** was formed in 66% yield (entry 12). Given that TfOH provided good reactivity as well, *p*TsOH•H_2_O was used as another comparative Brønsted acid. Carbazole **5aa** was formed in 55% yield along with 19% yield of furan **4aa** (entry 13). These control reactions demonstrate that while Lewis acid catalysis is definitely in play, we must acknowledge the presence of cooperative catalysis by TfOH. We decided to move forward with Yb(OTf)_3_ as the Lewis acid catalyst of choice given the high product yield. Further changes to either the temperature, solvent, or concentration failed to provide better product outcomes.

### 2.3. Benzannulation Substrate Scope

**Scheme 3:**
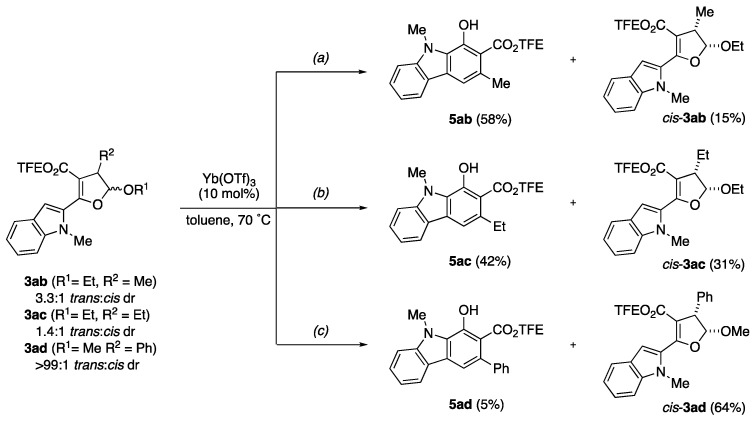
Unexpected Conversion Issues for 4-Substituted Dihydrofuran Acetals (a) **3ab**, (b) **3ac**, and (c) **3ad**.

With dihydrofuran **3aa** readily converting to carbazole **5aa** with high yield, we next sought to explore the benzannulation reaction with the other synthesized substrates. We first examined 3-substituted dihydrofuran acetals **3ab**–**3ad** (Scheme 3). Under the reactions, 2-ethoxy-3-methyl-substituted dihydrofuran **3ab** (as a 3.4:1 *trans*:*cis* diastereomeric mixture) gave partial conversion to the corresponding carbazole-2-carboxylate **5ab** in 58% yield (Scheme 3a). Interestingly, the recovered dihydrofuran (15%) was the *cis*-diastereomer. This outcome equates to a ~3.8:1 carbazole:*cis*-dihydrofuran ratio which closely parallels the initial dihydrofuran *trans*:*cis* ratio. Similar results were obtained with 3-ethyl substituted dihydrofuran **3ac** (as 1.4:1 *trans*:*cis* mixture). Carbazole **5ac** was formed in 42% yield along with 31% of recovered *cis*-dihydrofuran, a matching ~1.4:1 ratio (Scheme 3b). Seemingly contradictory, the *trans*-3-phenyl-substituted substrate **3ad** afforded only trace (~5%) benzannulation product (Scheme 3c). Instead, epimerization of the acetal center occurred to generate *cis*-**3ad** in 64% yield, which is fairly unreactive under the reaction conditions. It is plausible that the phenyl substituent participates in the mechanism through anchimeric assistance resulting in stabilization of the dihydrofuran and reversible ring-opening. Thus, we can infer that the *trans*-dihydrofuran isomers react faster than the corresponding *cis*-isomers under the reaction conditions.

**Scheme 4:**
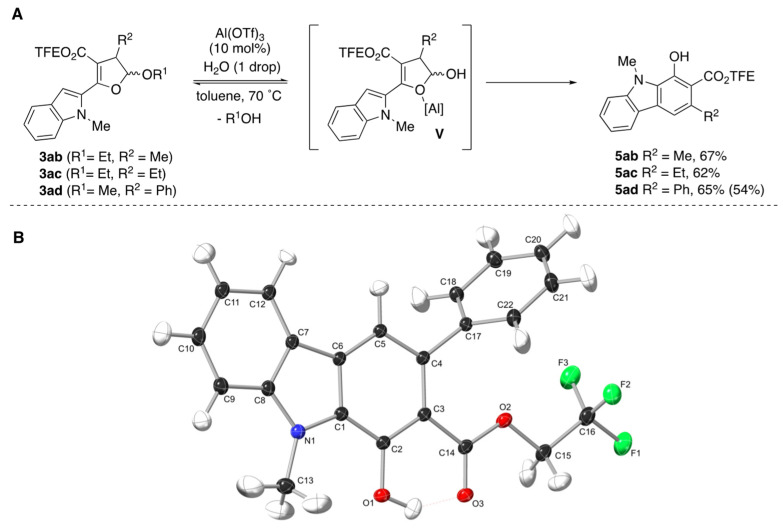
(**A**) Effect of Al(OTf)_3_ and H_2_O (1 drop) on Benzannulations of Dihydrofurans **3ab**–**3ad** and (**B**) the Crystal Structure of **5ad** (drawn at 50% probability level). Yield in parentheses represents product yield using TfOH (10 mol%).

To overcome this reactivity issue, we first turned to revisiting some of the other Lewis acids that effectively formed the carbazole. Disappointingly, similar outcomes were obtained whether In(OTf)_3_, Sc(OTf)_3_, or Al(OTf)_3_ was used in place of Yb(OTf)_3_. At this point, we went back to our previous work for inspiration and decided to try Al(OTf)_3_ with a drop of H_2_O [45]. These conditions previously prevented furan formation from the corresponding dihydrofuran acetals. Satisfyingly, subjecting dihydrofurans **3ab**–**3ad** to Al(OTf)_3_ and 1 drop of H_2_O, provided the desired carbazoles **5ab**, **5ac**, and **5ad** in 67%, 62%, and 65% yields, respectively (Scheme 4). Our rationale for the shift in reactivity with the added water is the likely formation of TfOH (in line with the catalyst screen) which facilitates generation of dihydrofuran hemiacetal intermediate **V** that undergoes ring-opening. To confirm our hypothesis, we treated **3ad** with TfOH (10 mol%) in toluene at 70 °C and obtained carbazole **5ad** in 54% yield.

**Figure 4:**
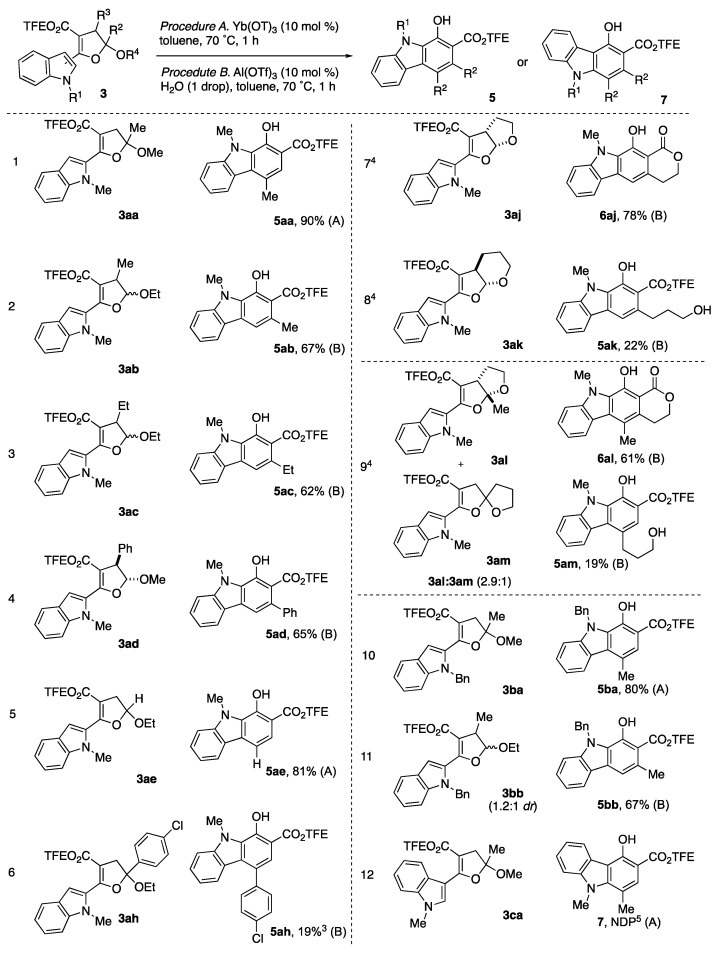
Substrate Scope of the Ring Opening Benzannulation of Dihydrofuran Acetals **3**. ^1^ *Procedure A*: Reaction performed with dihydrofuran acetal **3** and Yb(OTf)_3_ (10 mol%) in toluene (0.1 M) at 70 °C for 1 h. ^2^ *Procedure B*: Reaction performed with dihydrofuran acetal **3**, Al(OTf)_3_ (10 mol%), H_2_O (one drop) in toluene (0.1 M) at 70 °C for 1 h. ^3^ Inseparable mixture with furan **4ah**. ^4^ No water was added. ^5^ No desired product formed using either procedure. Obtained furan **4ca** instead.

With two sets of reaction conditions, we proceeded forward with the exploration of the substrate scope. Figure 4 summarizes the outcomes of the study, including those systems previously discussed (entries 1–4). The trisubstituted dihydrofuran **3ab** (derived from ethyl vinyl ether) smoothly converted using Yb(OTf)_3_ to its 1-hydroxy carbazole-2-carboxylate **5ab** in 81% yield (entry 5). For 2-(4-chlorophenyl)-2-methoxy dihydrofuran **3ah**, elimination to furan **4ah** was the predominant outcome observed for both Yb(OTf)_3_ and Al(OTf)_3_/H_2_O. The formed carbazole **5ah** (5% for Yb and 19% for Al) was inseparable from the furan product in both cases (entry 6). Next, we examined the fused bicyclic dihydrofurans **3aj** and **3ak** (entries 7 and 8). Dihydrofuran **3aj** was synthesized as the *cis*-diastereomer. Under the Al(OTf)_3_/H_2_O conditions, only furan **4aj** was obtained. After some minor optimization, we found that Al(OTf)_3_ without added water produced a 78% yield of lactono-carbazole **6aj** which resulted from the intramolecular transesterification of **5aj** (entry 7). Under the same conditions (Al(OTf)_3_ with no added water), tetrahydropyran-fused dihydrofuran acetal **3ak** readily provided carbazole **5ak** in 22% (entry 8). No lactonization was observed which is consistent with entropic and enthalpic considerations for seven-membered ring formation [50]. Based on the reactions with the bicyclic dihydrofurans, we rationalized two things relative to the tetrasubstituted monocyclic dihydrofurans **3ab**–**3ad**: (1) Diastereoselectivity does not seem to be a factor in the reactivity; (2) It is likely that hemiacetal formation is unfavorable with the added water due to the stability of the bicyclic acetal framework. When the mixture of fused-bicyclic dihydrofuran **3al** and spirocyclic dihydrofuran **3am** was subjected to the same conditions (Al(OTf)_3_, no water), lactono-carbazole **6al** was obtained in 61% yield along with 19% yield of carbazole **5am** (entry 9).

For the future purposes of synthesis, the effects of changing the *N*-methyl substituent to a *N*-benzyl group was explored. The corresponding carbazole **5ba** was obtained in 80% yield using Yb(OTf)_3_ for dihydrofuran **3ba** (entry 10), whereas Al(OTf)_3_/H_2_O was used to generate 67% yield of carbazole **5bb** from dihydrofuran **3bb** (entry 11). 

Lastly, we attempted the benzannulation of the 3-indolyl dihydrofuran **3ca** (entry 12). In all cases, regardless of what procedure or Lewis acid employed, only furan **4ca** was obtained.

### 2.4. Formal Synthesis of Murrayafoline A

**Scheme 5:**
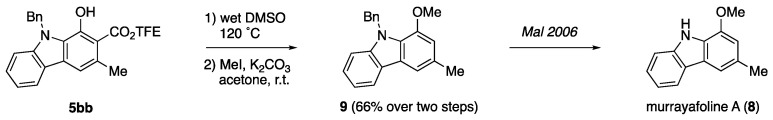
Formal Synthesis of Murrayafoline A (**8**) [30].

Using carbazole **5bb** as a precursor, we undertook the formal synthesis of murrayafoline A (**8**, Scheme 5). Murrayafoline A is a monomeric carbazolic alkaloid that has been isolated from kilograms of the dried powdered roots of several species of the genus *Murraya*, *Glycosmis*, and *Clausena* in up to 3% yield [51,52,53]. Murrayafoline A was shown to exhibit a number of interesting biological activities including strong fungicidal activity (12.5 µg dose against *C. cucumerinum*) [52], cancer growth inhibitory activity (HT-1080 cells) [53], and Wnt/β-catenin signaling antagonist activity [54]. It has been the target of numerous several syntheses due to its role as a precursor to access a variety of congeners and non-natural derivatives. From carbazole **5bb**, we could readily access *N*-benzyl murrayafoline A **9** in 66% yield over two steps following Krapcho decarbalkoxylation and *O*-methylation. Carbazole **9** has been shown by Mal and coworkers [47] to readily convert to the target molecule in in one step.

## 3. Experimental

**A. General.** All reactions were performed under protection of N_2_ in flame-dried glassware unless water was applied as solvent. Chromatographic purification was performed as flash chromatography with Silicycle SiliaFlash P60 silica gel (40–63 µm) or preparative thin-layer chromatography (prep-TLC) using silica gel F254 (1000 µm) plates and solvents indicated as eluent with 0.1–0.5 bar pressure. For quantitative flash chromatography, technical grades solvents were utilized. Analytical thin-layer chromatography (TLC) was performed on Silicycle SiliaPlate TLC silica gel F254 (250 µm) TLC glass plates. Visualization was accomplished with UV light. Infrared (IR) spectra were obtained via thin film IR on a salt plate using a Nicolet 6700 Fourier-transform infrared spectrophotometer. The IR bands are characterized as broad (br), weak (w), medium (m), and strong (s). Proton and carbon nuclear magnetic resonance spectra (Supplementary Materials: ^1^H NMR, ^13^C NMR and ^19^F NMR) were recorded on a Bruker 400 MHz spectrometer or on a Bruker 500 MHz spectrometer or on a Bruker 700 MHz spectrometer with solvent resonances as the internal standard (^1^H NMR: CDCl_3_ at 7.26 ppm; ^13^C NMR: CDCl_3_ at 77.0 ppm). ^1^H NMR data are reported as follows: chemical shift (ppm), multiplicity (s = singlet, d = doublet, dd = doublet of doublets, dt = doublet of triplets, ddd = doublet of doublet of doublets, t = triplet, q = quartet, p = pentet, m = multiplet, br = broad), coupling constants (Hz), and integration. Mass spectra were obtained through EI on a Micromass AutoSpec machine or through ESI on a Thermo Orbitrap XL. The accurate mass analyses run in EI mode were at a mass resolution of 10,000 and were calibrated using PFK (perfluorokerosene) as an internal standard. The accurate mass analyses run in EI mode were at a mass resolution of 30,000 using the calibration mixture supplied by Thermo. Crystal structures for carbazoles **5ad** (Deposition Number 2213553) and **5ai** (Deposition Number 2213554) were deposited in the Cambridge Structural Database. 

**B. Synthesis of Indolyl-α-diazo-β-ketoesters 1**. *General Procedure:* Using the following modified literature procedure [55]: To a dry flask charged with a stir bar and the corresponding indole carboxylic acid (1.0 equiv.), dry CH_2_Cl_2_ was added to make a 0.5 M solution, followed by addition of catalytic DMF (a few drops). The solution was then cooled to 0 °C and oxalyl chloride (1.2 equiv.) was slowly added over 1 min. After 15 min, the reaction was allowed to warm to room temperature with continued stirring. After 3 h at room temperature, the reaction was concentrated under reduced pressure and the acid chloride residue was dissolved in dry THF to make a 1 M solution (keeping it under inert atmosphere). The solution was added slowly to the prepared enolate at −78 °C. The enolate was prepared by first adding LHMDS (1 M in THF, 3.0 equiv.) to a dry flask charged with a stir bar under nitrogen and cooling to −78 °C. The corresponding acetate (1.05 equiv.) was added to the solution of LHMDS in one shot and stirred for 45 min at −78 °C. After 30 min from the addition of the acid chloride to the enolate solution, the reaction was quenched with 0.5 M HCl at −78 °C, extracted with EtOAc three times, dried using Na_2_SO_4_, and filtered through celite. The combined organic layers were concentrated under reduced pressure and purified by flash chromatography on silica gel using 15–30% EtOAc/Hexanes as the mobile phase to produce pure product II. To an ice-cold solution of II (1.0 equiv.) and 4-acetamidobenzenesulfonyl azide (*p*-ABSA, 1.05 equiv.) in MeCN (0.2 M), NEt_3_ (1.1 equiv.) was slowly added. The reaction was left to warm gradually to room temperature overnight. Upon completion, the reaction mixture was vacuum-filtered through a celite plug to remove the formed solids. The filtrate was concentrated under vacuum and the obtained residue was purified on silica gel (15–30% EtOAC/Hexanes) to afford pure diazo compound **1**.

*2,2,2-Trifluoroethyl 2-diazo-3-(1-methyl-1H-indol-2-yl)-3-oxopropanoate (***1a***)*: Prepared by general diazo synthesis route, and started with *N*-methylindole-2-carboxylic acid (5.00 g, 28.5 mmol). It afforded 7.76 g (23.9 mmol, 84% over 3 steps) final product **1a** as a yellow solid. **^1^H NMR** (700 MHz, CDCl_3_) δ 7.69 (dt, *J* = 8.1, 1.0 Hz, 1H), 7.42–7.36 (m, 2H), 7.20 (s, 1H), 7.17 (ddd, *J* = 7.9, 5.0, 2.7 Hz, 1H), 4.65 (q, *J* = 8.2 Hz, 2H), 3.97 (s, 3H). **^13^C NMR** (176 MHz, CDCl_3_) δ 175.5, 159.6, 140.1, 132.3, 126.1, 125.6, 123.1, 122.6 (q, *J* = 277.7 Hz), 120.9, 111.9, 110.2, 60.5 (q, *J* = 37.4 Hz), 31.8. **IR** 2950.79 (w), 2142.71 (s), 1741.70 (s), 1728.65 (s), 1613.26 (m), 1315.51 (s), 1274.18 (s), 1170. 92 (s), 1108.74 (m). **HMRS** (ESI) *m*/*z*: [M + H]^+^ calc. for. C_14_H_10_F_3_N_3_O_3_, 326.0747; Found 326.0748. 

*2,2,2-Trifluoroethyl 3-(1-benzyl-1H-indol-2-yl)-2-diazo-3-oxopropanoate (***1b***)*: Prepared by general diazo synthesis route, and started with *N*-benzylindole-2-carboxylic acid (2.00 g, 7.96 mmol) [55]. It afforded 2.46 g (6.14 mmol, 77% over 3 steps) final product **1b** as a yellow gel. **^1^H NMR** (500 MHz, CDCl_3_) δ 7.74 (d, *J* = 8.1 Hz, 1H), 7.34 (d, *J* = 3.4 Hz, 2H), 7.30 (s, 1H), 7.28–7.17 (m, 4H), 7.06–7.02 (m, 2H), 5.71 (s, 2H), 4.63 (q, *J* = 8.2 Hz, 2H). **^13^C NMR** (126 MHz, CDCl_3_) δ 175.6, 159.5, 139.8, 137.9, 132.2, 128.6, 127.2, 126.3, 126.2, 125.8, 123.1, 122.6 (q, J = 277.5 Hz), 121.2, 112.7, 110.8, 75.3, 60.5 (q, J = 37.1 Hz), 48.0. **IR** 3031.90 (m), 2139.42 (s), 1764.60 (s), 1717.14 (m), 1665.47 (s), 1283.34 (m), 1167.42 (s), 1141.86 (m). **HMRS** (ESI) *m*/*z*: [M + H]^+^ calc. C_20_H_14_F_3_N_3_O_3_, 402.1060; Found 402.1060.

*2,2,2-Trifluoroethyl 2-diazo-3-(1-methyl-1H-indol-3-yl)-3-oxopropanoate (***1c***)*: Prepared by general diazo synthesis route, and started with *N*-methylindole-3-carboxylic acid (5.00 g, 28.5 mmol). It afforded 7.32 g (22.5 mmol, 79.0% over 3 steps) final product **1c** as a yellow solid. **^1^H NMR** (700 MHz, CDCl_3_) δ 8.38–8.34 (m, 1H), 8.19 (s, 1H), 7.39–7.28 (m, 3H), 4.63 (q, *J* = 8.3 Hz, 2H), 3.85 (s, 3H). **^13^C NMR** (176 MHz, CDCl_3_) δ 176.2, 160.3, 137.6, 136.8, 127.6, 123.5, 122.9, 122.8 (q, *J* = 277.7 Hz), 122.5, 113.1, 109.6, 60.3 (q, *J* = 37.1 Hz), 33.7. **IR** 3148. 66 (m), 3012.10 (w), 2916.12 (w), 2148.29 (s), 1740.71 (s), 1731.42 (s), 1583.12 (m), 1307.18 (s), 1279.46 (s), 1167.31 (s), 1120.22 (m). **HMRS** (ESI) *m*/*z*: [M + H]^+^ calc. for. C_14_H_10_F_3_N_3_O_3_, 326.0747; Found 326.0731. 

**C. Synthesis of 2,3-dihydrofuran acetals 3.***General Procedure:* To a dry flask charged with a stir bar and a solution of Cu(hfacac)_2_ (29 mg, 61.5 μmol) and 2 (3.07 mmol) in anhydrous CH_2_Cl_2_ (6 mL) was added diazo **1** (615 μmol). The reaction was stirred vigorously under reflux condition for overnight or monitored by TLC for completion. After consuming all starting diazo, the mixture was diluted with Et_2_O (30 mL) and washed with saturated thiourea (20 mL). The organic layer was dried over Na_2_SO_4_, concentrated under reduced pressure, and purified by column chromatography. Since the ^19^F signals of all DHF acetal products have similar chemical shifts (−73.3 ppm to −73.8 ppm) and coupling constant (t, *J* = 8.5 Hz), only the **^19^F NMR** of **3aa** is reported and attached.

*2,2,2-Trifluoroethyl 5-methoxy-5-methyl-2-(1-methyl-1H-indol-2-yl)-4,5-dihydrofuran-3-carboxylate (***3aa***):* Prepared following general procedure using 2-methoxypropene **2a** (221 mg, 3.07 mmol) and diazo **1a** (200 mg, 615 μmol). Purification via silica gel column chromatography (5% EtOAC/Hexanes, R_f_ = 0.39 in 20% EtOAC/Hexanes) afforded **3aa** as pale-yellow solid (181 mg, 79%). **^1^H NMR** (700 MHz, CDCl_3_) δ 7.65 (dt, *J* = 8.0, 1.0 Hz, 1H), 7.34 (dq, *J* = 8.4, 1.0 Hz, 1H), 7.30 (ddd, *J* = 8.3, 6.9, 1.2 Hz, 1H), 7.17 (d, *J* = 1.0 Hz, 1H), 7.13 (ddd, *J* = 8.0, 6.9, 1.1 Hz, 1H), 4.47 (qq, *J* = 8.5, 4.2 Hz, 2H), 3.81 (s, 3H), 3.43 (s, 3H), 3.21 (d, *J* = 16.6 Hz, 1H), 3.09 (d, *J* = 16.6 Hz, 1H), 1.73 (s, 3H). **^13^C NMR** (176 MHz, CDCl_3_) δ 162.3, 158.3, 138.7, 127.7, 126.8, 123.9, 123.15 (q, *J* = 277.0 Hz), 121.9, 120.1, 111.6, 109.7, 108.9, 102.7, 59.82 (q, J = 36.4 Hz), 50.4, 40.5, 31.8, 24.5. **^19^F NMR** (471 MHz, CDCl_3_) δ −73.43 (t, *J* = 8.5 Hz, 3F). **IR** 3050.91(m), 2945.23, 2836.57(m), 1720.57(s), 1711.31(s), 1168.64(s), 1106.59(s), 1047.59(s). **HMRS** (ESI) *m*/*z*: [M + H]^+^ calc. for C_18_H_18_F_3_NO_4_, 370.1260; Found 370.1262.

*2,2,2-Trifluoroethyl 2-(1-benzyl-1H-indol-2-yl)-5-methoxy-5-methyl-4,5-dihydrofuran-3-carboxylate (***3ba***):* Prepared following general procedure using 2-methoxypropene **2a** (221 mg, 3.07 mmol) and diazo **1b** (247 mg, 615 μmol). Purification via silica gel column chromatography (5% EtOAC/Hexanes, R_f_ = 0.52 in 20% EtOAC/Hexanes) afforded **3ba** as pale-yellow solid (192 mg, 70%). **^1^H NMR** (500 MHz, CDCl_3_) δ 7.69 (d, *J* = 7.9 Hz, 1H), 7.29 (s, 1H), 7.28–7.16 (m, 5H), 7.14 (ddd, *J* = 8.0, 6.3, 1.6 Hz, 1H), 7.00 (d, *J* = 6.9 Hz, 2H), 5.49 (s, 2H), 4.47 (qd, *J* = 8.5, 2.1 Hz, 2H), 3.17 (s, 3H), 3.13 (d, *J* = 16.7 Hz, 1H), 2.99 (d, *J* = 16.6 Hz, 1H), 1.57 (s, 3H). **^13^C NMR** (126 MHz, CDCl_3_) δ 162.3, 158.0, 138.3, 138.0, 128.5, 127.6, 127.1, 127.1, 126.0, 124.0, 123.1 (d, *J* = 277.4 Hz), 121.9, 120.4, 111.6, 110.3, 109.7, 102.9, 59.8 (q, *J* = 36.3 Hz), 50.2, 48.5, 40.6, 24.1. **IR** 3061.65(m), 3032.41(m), 2930.32(m), 2851.80(m), 1720.26(s), 1708.31(s), 1167.72(s), 1160.70(s), 1048.76(s). **HMRS** (ESI) *m*/*z*: [M + H]^+^ calc. for C_24_H_22_F_3_NO_4_, 446.1573; Found 446.1576.

*2,2,2-Trifluoroethyl 5-ethoxy-4-methyl-2-(1-methyl-1H-indol-2-yl)-4,5-dihydrofuran-3-carboxylate (***3ab***)*: Prepared following general procedure using 1-ethoxypropene (mixture of *cis*:*trans* = 2.7:1) **2b** (265 mg, 3.07 mmol) and diazo **1a** (200 mg, 615 μmol). Purification via silica gel column chromatography (5% EtOAC/Hexanes, R_f_ = 0.45 in 20% EtOAC/Hexanes) afforded **3ab** (167 mg, 70%) as a mixture of diastereomers (*dr* = 3.3:1). **^1^H NMR** (500 MHz, CDCl_3_) for *trans* isomer: δ 7.64 (d, *J* = 7.7 Hz, 1H), 7.35–7.27 (m, 2H), 7.13 (d, *J* = 6.6 Hz, 1H), 7.03 (s, 1H), 5.74 (d, *J* = 7.5 Hz, 1H), 4.57–4.48 (m, 1H), 4.44–4.35 (m, 1H), 3.78 (s, 3H), 3.74–3.66 (m, 1H), 3.51 (p, *J* = 7.1 Hz, 1H), 1.34 (d, *J* = 7.0 Hz, 3H), 1.29 (t, *J* = 7.1 Hz, 3H); For *cis* isomer: δ 7.64 (d, *J* = 7.7 Hz, 1H), 7.35–7.27 (m, 2H), 7.16–7.09 (m, 2H), 5.27 (d, *J* = 1.8 Hz, 1H), 4.57–4.48 (m, 1H), 4.44–4.35 (m, 1H), 3.80 (s, 3H), 3.74–3.66 (m, 1H), 3.27 (qd, *J* = 7.1, 1.9 Hz, 1H),1.34 (d, *J* = 7.0 Hz, 3H), 1.29 (t, *J* = 7.1 Hz, 3H); **^13^C NMR** (126 MHz, CDCl_3_) for *trans* isomer (major isomer): δ 162.6, 158.1, 138.5, 128.2, 126.8, 123.51, 121.7, 120.0, 111.5, 109.6, 107.9, 107.8, 65.8, 59.6 (q, *J* = 36.3 Hz), 40.8, 31.5, 15.0, 11.8. The ^13^C signal that is directly coupled by fluorine is not reported due to the low intensity. **IR** 3058.43 (m), 2978.89 (s), 2933.93 (s), 1720.49 (s), 1711.45 (s), 1626.02 (m), 1281.91 (m), 1167.26 (s), 1084.81(s). **HMRS** (ESI) *m*/*z*: [M + H]^+^ calc. for C_19_H_20_F_3_NO_4_, 384.1417; Found 384.1417.

*2,2,2-Trifluoroethyl 2-(1-benzyl-1H-indol-2-yl)-5-ethoxy-4-methyl-4,5-dihydrofuran-3-carboxylate (***3bb***):* Prepared following general procedure but set up **as 1 g (2.5 mmol) scale**: using 1-ethoxypropene (mixture of *cis*:*trans* = 1.2:1) **2b** (1.07 g, 12.46 mmol), diazo **1b** (1.00 g, 2.49 mmol) and Cu(hfacac)_2_ (119 mg, 0.249 mmol) in anhydrous CH_2_Cl_2_ (24 mL). Purification via silica gel column chromatography (5% EtOAC/Hexanes, R_f_ = 0.48 in 20% EtOAC/Hexanes) afforded **3bb** as the mixture of diastereomers (*dr* = 1.81: 1) (800 mg, 70%). **^1^H NMR** (500 MHz, CDCl_3_) for *trans* isomer: δ 7.74–7.67 (m, 1H), 7.30–7.19 (m, 5H), 7.18–7.11 (m, 2H), 7.07 (d, *J* = 6.8 Hz, 2H), 5.60 (d, *J* = 7.5 Hz, 1H), 5.46 (d, *J* = 6.5 Hz, 1H), 5.44 (d, *J* = 6.5 Hz, 1H), 4.59–4.47 (m, 1H), 4.48–4.36 (m, 1H), 3.68–3.55 (m, 1H), 3.52–3.46 (m, 1H), 3.42 (p, *J* = 7.1 Hz, 1H), 1.28 (d, *J* = 7.1 Hz, 3H), 1.16 (d, *J* = 7.1 Hz, 3H); For *cis* isomer: δ 7.74–7.67 (m, 1H), 7.30–7.19 (m, 5H), 7.18–7.11 (m, 2H), 7.07 (d, *J* = 6.8 Hz, 2H), 5.49 (d, *J* = 6.5 Hz, 1H), 5.46 (d, *J* = 4.7 Hz, 1H), 5.17 (d, *J* = 1.9 Hz, 1H), 4.59–4.47 (m, 1H), 4.48–4.36 (m, 1H), 3.68–3.55 (m, 1H), 3.52–3.46 (m, 1H), 3.23 (qd, J = 7.1, 2.0 Hz, 1H), 1.28 (d, *J* = 7.1 Hz, 3H), 1.15 (d, *J* = 7.1 Hz, 3H). **^13^C NMR** (126 MHz, CDCl_3_) for both diastereomers: δ 162.4, 162.2, 157.80, 157.78, 138.2, 138.1, 138.0, 137.9, 128.42, 128.38, 127.2, 127.1, 126.2, 126.0, 123.9, 123.7, 121.9, 121.8, 120.3, 120.2, 111.5, 110.3, 110.2, 109.5, 109.4, 109.1, 109.0, 107.7, 65.5, 64.6, 59.7 (q, *J* = 36.3 Hz) 59.6 (q, *J* = 36.3 Hz), 48.49, 48.46, 44.0, 40.7, 17.4, 14.9, 14.8, 11.6. **IR** 3063.84 (w), 2977.64 (m), 2930.38 (m), 1711.27 (s), 1708.59 (s), 1281.37 (s), 1167.60 (s). **HMRS** (ESI) *m*/*z*: [M + H]^+^ calc. for C_25_H_24_F_3_NO_4_, 460.1730; Found 460.1731. 

*2,2,2-Trifluoroethyl 5-ethoxy-4-ethyl-2-(1-methyl-1H-indol-2-yl)-4,5-dihydrofuran-3-carboxylate (***3ac***):* Prepared following general procedure using 1-ethoxybutene (mixture of *cis*:*trans* = 1.5:1) **2c** (308 mg, 3.07 mmol) and diazo **1a** (200 mg, 615 μmol). Purification via silica gel column chromatography (5% EtOAC/Hexanes, R_f_ = 0.48 in 20% EtOAC/Hexanes) afforded **3ac** as the mixture of diastereomers (*dr* = 1.4:1) (179 mg, 73%). **^1^H NMR** (400 MHz, CDCl_3_) for *trans* isomer: δ 7.65 (d, *J* = 7.0 Hz, 1H), 7.39–7.25 (m, 2H), 7.19–7.09 (m,1H), 7.02 (d, *J* = 0.9 Hz, 1H), 5.78 (d, *J* = 7.4 Hz, 1H), 4.58–4.33 (m, 2H), 3.98 (dqd, *J* = 9.6, 7.1, 3.9 Hz, 1H), 3.78 (s, 3H), 3.78–3.66 (m, 1H), 3.38 (ddd, *J* = 9.3, 7.3, 3.5 Hz, 1H), 1.96–1.79 (m, 2H), 1.30 (t, *J* = 6.9 Hz, 3H), 1.06 (t, *J* = 7.4 Hz, 3H); For *cis* isomer: δ 7.66 (d, *J* = 7.0 Hz, 1H), 7.39–7.25 (m, 2H), 7.19–7.09 (m, 2H), 5.37 (d, *J* = 1.9 Hz, 1H), 4.58–4.33 (m, 2H), 3.98 (dqd, *J* = 9.6, 7.1, 3.9 Hz, 1H), 3.78 (s, 3H), 3.78–3.66 (m, 1H), 3.21 (ddd, *J* = 8.6, 3.9, 1.9 Hz, 1H), 1.73–1.55 (m, 2H),1.32 (t, *J* = 6.9 Hz, 3H), 1.02 (t, *J* = 7.4 Hz, 3H). **^13^C NMR** (101 MHz, CDCl_3_) for both diastereomers: δ 162.6, 162.3, 158.3, 138.6, 138.4, 128.4, 128.1, 126.9, 126.8, 123.7, 123.5, 121.8, 121.6, 120.03, 119.95, 109.7, 109.6, 108.6, 107.8, 107.68, 107.65, 107.2, 65.9, 64.7, 59.7 (q, *J* = 36.3 Hz), 59.6 (q, *J* = 36.3 Hz), 50.4, 47.2, 31.5, 31.4, 24.3, 19.6, 15.1, 15.0, 12.3, 10.4. The ^13^C signal that is directly coupled by fluorine is not reported due to the low intensity. **IR** 2968.85 (m), 2936.33 (w), 2877.54 (w), 1720.23 (s), 1711.33 (m), 1277.33 (m), 1168.55 (s), 1084.69 (s). **HMRS** (ESI) *m*/*z*: [M + H]^+^ calc. for C_20_H_22_F_3_NO_4_, 398.1574; Found 398.1576. 

*2,2,2-Trifluoroethyl 5-methoxy-2-(1-methyl-1H-indol-2-yl)-4-phenyl-4,5-dihydrofuran-3-carboxylate (***3ad***):* Prepared following general procedure using beta-methoxystyrene (pure *cis* isomer) **2d** (413 mg, 3.07 mmol) and diazo **1a** (200 mg, 615 μmol). Purification via silica gel column chromatography (5% EtOAC/Hexanes, R_f_ = 0.33 in 20% EtOAC/Hexanes) afforded **3ad** as yellow gel (140 mg, 53%). **^1^H NMR** (500 MHz, CDCl_3_) δ 7.69 (dt, *J* = 7.9, 1.0 Hz, 1H), 7.40–7.29 (m, 7H), 7.25 (d, *J* = 0.9 Hz, 1H), 7.16 (ddd, *J* = 7.9, 6.8, 1.1 Hz, 1H), 5.86 (d, *J* = 7.8 Hz, 1H), 4.66 (d, *J* = 7.8 Hz, 1H), 3.89 (s, 3H), 3.50 (s, 3H). **^13^C NMR** (126 MHz, CDCl_3_) δ 162.0, 159.0, 138.8, 135.7, 129.2, 128.0, 127.6, 127.2, 126.8, 123.9, 122.8 (q, *J* = 277.5 Hz), 121.9, 120.2, 109.7, 108.9, 108.6, 107.9, 59.6 (q, *J* = 36.5 Hz), 57.7, 52.5, 31.8. **IR** 3061.65 (w), 3030.38 (w), 2937.40 (m), 2845.72 (w), 1720.41 (s), 1711.54 (m), 1277.50 (m), 1168.60 (s), 1092.46 (s). **HMRS (ESI)** *m*/*z*: [M + H]^+^ calc. for C_23_H_20_F_3_NO_4_, 432.1417; Found 432.1418. 

*2,2,2-Trifluoroethyl 5-ethoxy-2-(1-methyl-1H-indol-2-yl)-4,5-dihydrofuran-3-carboxylate (***3ae***):* Prepared following general procedure using ethoxyethene **2e** (413 mg, 3.07 mmol) and diazo **1a** (200 mg, 615 μmol). Purification via silica gel column chromatography (5% EtOAC/Hexanes, R_f_ = 0.39 in 20% EtOAC/Hexanes) afforded **3ae** as yellow solid (171 mg, 75%). **^1^H NMR** (500 MHz, CDCl_3_) δ 7.66 (dt, J = 8.0, 1.0 Hz, 1H), 7.37–7.28 (m, 2H), 7.16 (d, *J* = 0.8 Hz, 1H), 7.14 (ddd, *J* = 7.9, 6.8, 1.1 Hz, 1H), 5.75 (dd, *J* = 7.4, 2.8 Hz, 1H), 4.55–4.41 (m, 2H), 3.97 (dq, *J* = 9.5, 7.1 Hz, 1H), 3.81 (s, 3H), 3.71 (dq, *J* = 9.5, 7.1 Hz, 1H), 3.34 (dd, *J* = 16.6, 7.4 Hz, 1H), 3.05 (dd, *J* = 16.6, 2.8 Hz, 1H), 1.30 (t, *J* = 7.1 Hz, 3H). **^13^C NMR** (126 MHz, CDCl_3_) δ 162.3, 158.1, 138.5, 127.8, 126.8, 123.7, 123.1 (q, *J* = 277.3 Hz), 121.8, 120.0, 109.7, 108.7, 105.2, 102.8, 64.8, 59.7 (q, *J* = 36.4 Hz), 37.2, 31.7, 15.1. **IR** 2977.71 (m), 2938.65 (w), 1720.30 (s), 1711.16 (m), 1629.92 (m), 1283.07 (s), 1247.21 (s), 1169.49 (s), 1080.27 (s). **HMRS (ESI)** *m*/*z*: [M + H]^+^ calc. for C_18_H_18_F_3_NO_4_, 370.1261; Found 370.1262. 

*2,2,2-Trifluoroethyl 5-(4-chlorophenyl)-5-ethoxy-2-(1-methyl-1H-indol-2-yl)-4,5-dihydrofuran-3-carboxylate (***3ah***):* Prepared following general procedure using 1-chloro-4-(1-ethoxyvinyl)benzene **2h** (562 mg, 3.07 mmol, which was prepared according to previous reported literature [46]) and diazo **1a** (200 mg, 615 μmol). Purification via silica gel column chromatography (5% EtOAC/Hexanes, R_f_ = 0.52 in 20% EtOAC/Hexanes) afforded **3ah** as yellow solid (192 mg, 65%). **^1^H NMR** (500 MHz, CDCl_3_) δ 7.71 (dt, *J* = 8.0, 1.0 Hz, 1H), 7.50–7.47 (m, 2H), 7.44–7.39 (m, 3H), 7.35 (ddd, *J* = 8.3, 6.9, 1.2 Hz, 1H), 7.28 (d, *J* = 0.8 Hz, 1H), 7.18 (ddd, *J* = 8.0, 6.8, 1.1 Hz, 1H), 4.50 (qd, *J* = 8.5, 2.6 Hz, 2H), 3.90 (s, 3H), 3.71 (dq, *J* = 9.5, 7.1 Hz, 1H), 3.57 (d, *J* = 16.6 Hz, 1H), 3.41 (dq, *J* = 9.4, 7.1 Hz, 1H), 3.31 (d, *J* = 16.6 Hz, 1H), 1.24 (t, *J* = 7.0 Hz, 3H). **^13^C NMR** (126 MHz, CDCl_3_) δ 162.1, 157.7, 138.7, 138.6, 134.6, 128.9, 127.7, 127.1, 126.8, 123.9, 123.1 (q, *J* = 277.5 Hz), 121.8, 120.2, 111.4, 109.7, 108.9, 103.5, 59.9 (d, *J* = 36.4 Hz), 59.7, 44.8, 31.9, 15.3. **IR** 3058.41 (w), 2978.18 (m), 2936.26 (w), 1724.57 (s), 1711.38 (m), 1283.63 (m), 1234.06 (m), 1171.08 (s), 1093.74 (s). **HMRS (ESI)** *m*/*z*: [M + H]^+^ calc. for C_24_H_21_ClF_3_NO_4_, 480.1184; Found 480.1186. 

*Reaction of Diazo***1a***and Trimethyl((1-phenylvinyl)oxy)silane***2i**. Following the general procedure using trimethyl((1-phenylvinyl)oxy)silane **2i** (591 mg, 3.07 mmol) and diazo **1a** (200 mg, 615 μmol), an inseparable mixture of dihydrofuran **3ai**, carbazole **5ai**, and acetophenone (hydrolysis product of enol silane **2i**) was isolated following column chromatography (1–5% EtOAC/Hexanes). According to quantitative ^1^H-NMR (with DMF as the internal standard), the yields of **3ai** and **5ai** were 50% and 21%, respectively. 

*2,2,2-Trifluoroethyl 2-(1-methyl-1H-indol-2-yl)-3a,4,5,6a-tetrahydrofuro[2,3-b]furan-3-carboxylate (***3aj***):* Prepared following general procedure using dihydrofuran **2j** (215 mg, 3.07 mmol), diazo **1a** (200 mg, 615 μmol) and 20 mol% Cu(hfacac)_2_ (59 mg, 123 μmol). This reaction was kept at reflux for 2 d. Purification via silica gel column chromatography (5% EtOAC/Hexanes, R_f_ = 0.3 in 20% EtOAC/Hexanes) afforded **3aj** as yellow gel (112 mg, 50%). **^1^H NMR** (500 MHz, CDCl_3_) δ 7.68 (dt, *J* = 7.9, 1.0 Hz, 1H), 7.37–7.30 (m, 2H), 7.25 (s, 1H), 7.15 (ddd, *J* = 7.9, 6.6, 1.4 Hz, 1H), 6.34 (d, *J* = 6.3 Hz, 1H), 4.63 (dq, *J* = 12.7, 8.5 Hz, 1H), 4.45 (dq, *J* = 12.7, 8.5 Hz, 1H), 4.19 (ddd, *J* = 8.7, 5.6, 2.4 Hz, 1H), 4.07–4.02 (m, 1H), 3.90–3.84 (m, 1H), 3.82 (s, 3H), 2.25 (dt, *J* = 8.4, 4.0 Hz, 2H). **^13^C NMR** (126 MHz, CDCl_3_) δ 162.0, 160.6, 138.7, 127.0, 126.6, 124.0, 123.1 (q, *J* = 277.5 Hz), 121.8, 120.1, 109.9, 109.7, 109.3, 104.1, 67.1, 59.7 (q, *J* = 36.3 Hz), 47.6, 31.9, 31.8. **IR** 2958.56 (m), 2881.14 (m), 1721.91 (s), 1711.23 (m), 1619.47 (m), 1280.55 (s), 1233.10 (s), 1168.90 (s), 1077.28 (s). **HMRS (ESI)** *m*/*z*: [M + H]^+^ calc. for C_18_H_16_F_3_NO_4_, 368.1104; Found 368.1104. 

*2,2,2-Trifluoroethyl 2-(1-methyl-1H-indol-2-yl)-3a,5,6,7a-tetrahydro-4H-furo[2,3-b]pyran-3-carboxylate (***3ak***):* Prepared following general procedure using 2,3-dihydropyran **2k** (259 mg, 3.07 mmol), diazo **1a** (200 mg, 615 μmol) and 20 mol% Cu(hfacac)_2_ (59 mg, 123 μmol). The reaction was kept at reflux for 3 d for complete consumption of starting material. Purification via silica gel column chromatography (5% EtOAC/Hexanes, R_f_ = 0.36 in 20% EtOAC/Hexanes) afforded **3ak** as yellow gel. DMF was applied as internal NMR standard to quantify the yield (54%), since there were some undefined impurities. **^1^H NMR** (500 MHz, CDCl_3_) δ 7.65 (d, *J* = 8.0 Hz, 1H), 7.36–7.28 (m, 2H), 7.21 (s, 1H), 7.12 (ddd, *J* = 7.9, 6.8, 1.2 Hz, 1H), 6.03 (d, *J* = 7.3 Hz, 1H), 4.55 (dq, *J* = 12.8, 8.5 Hz, 1H), 4.45 (dq, *J* = 12.7, 8.5 Hz, 1H), 3.94–3.86 (m, 2H), 3.82 (s, 3H), 3.21 (td, *J* = 7.3, 6.2 Hz, 1H), 2.23–2.13 (m, 1H), 1.79–1.70 (m, 3H). **^13^C NMR** (126 MHz, CDCl_3_) δ 162.5, 159.3, 138.9, 127.6, 126.8, 124.0, 123.2 (q, *J* = 277.7 Hz), 121.9, 120.2, 109.8, 109.4, 108.1, 104.8, 61.7, 59.7 (q, *J* = 36.4 Hz), 38.2, 31.9, 23.5, 20.3. **IR** 3057.59 (w), 2949.15 (m), 1720.38 (s), 1711.18 (m), 1618.58 (m), 1613.47 (m), 1279.06 (m), 1161.26 (s), 1093.65 (s). **HMRS (ESI)** *m*/*z*: [M + H]^+^ calc. for C_19_H_18_F_3_NO_4_, 382.1261; Found 382.1261.

*Reaction of Diazo***1a***and 5-methyl 2,3-dihydrofuran (***2l***).* Following the general procedure with commercial 5-methyl 2,3-dihydropyran **2l** (259 mg, 3.07 mmol), diazo **1a** (200 mg, 615 μmol) and 20 mol% Cu(hfacac)_2_ (59 mg, 123 μmol), a 2.85:1 mixture (50% total yield according to ^1^H-NMR) of dihydrofurans **3al** and **3am** was isolated due to the in situ isomerization of **2l** to 2-methylene tetrahydrofuran (**2m**) in the reaction pot. Interestingly, if synthesized 5-methyl 2,3-dihydropyran **2l** [56] was used (see Supporting Information for ^1^H-NMR) instead of the commercial material, only **3am** was isolated as the major product when the reaction was run at 65 °C. Carbazole **5ai** could be prepared directly in 36% yield by the reaction of diazo **1a** and **2i** using Cu(hfacac)_2_ (30 mol%) with 1,2-dichloroethane as the solvent at 80 °C for 24 h. 

*2,2,2-Trifluoroethyl 2-(1-methyl-1H-indol-2-yl)-1,6-dioxaspiro[4.4]non-2-ene-3-carboxylate (***3am***):* Prepared according to what is described previously. Purification via silica gel column chromatography (5% EtOAc/Hexanes, R_f_ = 0.3 in 20% EtOAc/Hexanes) afforded **3am** as yellow gel (132 mg, 56%). **^1^H NMR** (500 MHz, CDCl_3_) δ 7.63 (dd, *J* = 7.9, 1.0 Hz, 1H), 7.34–7.26 (m, 2H), 7.14–7.09 (m, 2H), 4.53–4.40 (m, 2H), 4.19–4.09 (m, 2H), 3.78 (s, 3H), 3.31 (d, *J* = 16.6 Hz, 1H), 3.27 (d, *J* = 16.6 Hz, 1H), 2.47–2.42 (m, 1H), 2.26–2.17 (m, 1H), 2.16–2.07 (m, 2H). **^13^C NMR** (126 MHz, CDCl_3_) δ 162.3, 157.8, 138.6, 127.9, 126.8, 123.7, 123.2 (q, *J* = 277.3 Hz), 121.8, 120.0, 117.7, 109.6, 108.6, 102.6, 69.1, 59.7 (q, *J* = 36.4 Hz), 38.5, 36.7, 31.6, 23.9. **IR** 3058.34 (w), 2922.83 (s), 2853.16 (m), 1720.57 (s), 1614.31 (s), 1463.21 (m), 1284.80 (s), 1171.14 (s). **HMRS (ESI)** *m*/*z*: [M + H]^+^ calc. for 382.1261; Found 382.1261. 

*2,2,2-Trifluoroethyl 5-methoxy-5-methyl-2-(1-methyl-1H-indol-3-yl)-4,5-dihydrofuran-3-carboxylate (***3ca***):* Prepared following general procedure using 2-methoxypropene **2a** (221 mg, 3.07 mmol) and diazo **1c** (200 mg, 615 μmol). The reaction reached completion within 1 h under reflux. Purification via silica gel column chromatography (5% EtOAC/Hexanes, R_f_ = 0.5 in 20% EtOAC/Hexanes) afforded **3ca** as yellow solid (132 mg, 58%). **^1^H NMR** (500 MHz, CDCl_3_) δ 8.88 (s, 1H), 8.19 (d, *J* = 8.0 Hz, 1H), 7.38 (d, *J* = 8.2 Hz, 1H), 7.32 (t, 1H), 7.27 (d, *J* = 1.1 Hz, 1H), 4.63–4.50 (m, 2H), 3.89 (s, 3H), 3.44 (s, 3H), 3.24–3.04 (m, 2H), 1.78 (s, 3H). **^13^C NMR** (126 MHz, CDCl_3_) δ 164.2, 163.5, 136.8, 136.6, 126.9, 123.5 (d, *J* = 277.6 Hz), 122.7, 122.7, 121.5, 110.5, 109.7, 104.4, 94.5, 59.5 (q, *J* = 35.8 Hz), 50.2, 39.7, 33.5, 25.1. **IR** 3113.53 (w), 2938.52 (w), 1763.42 (s), 1759.62 (s), 1653.54 (s), 1530.37 (m), 1282.47 (m), 1169.54 (s), 1087.58 (m). **HMRS (ESI)** *m*/*z*: [M + H]^+^ calc. for C_18_H_18_F_3_NO_4_, 370.1260; Found 370.1262. 

**D. Ring-opening benzannulation of DHF toward 1-hydroxycarbazoles 5 and 6.**

*General procedure A*: To a vial or pressured tube charged with a stir bar and a solution of DHF **3** in toluene (0.1 M) was added Yb(OTf)_3_ (10 mol%). Then, the suspension was set under sonication for 1 min. The reaction was stirred under 70 °C for 1 h or monitored by TLC for completion. After consuming all DHF, the mixture was concentrated under reduced pressure, and purified by column chromatography.

*General procedure B*: To a vial or pressured tube charged with a stir bar and a solution of DHF **3** in toluene (0.1 M) was added Al(OTf)_3_ (10 mol%). Then, the suspension was set under sonication for 1 min, and 1 small drop of water (~4 mg) was added to the mixture. After the mixture was sonicated for another 5 min, the reaction was stirred vigorously under 70 °C for 1 h. After consuming all DHF, the mixture was concentrated under reduced pressure, and purified by column chromatography. 

Note: Since the ^19^F signals of all carbazole products have similar chemical shifts (−73.3 ppm to −73.8 ppm) with same coupling constant (t, *J* = 8.5 Hz), only the ^19^F NMR of **5aa** is reported and attached.

*2,2,2-Trifluoroethyl 1-hydroxy-4,9-dimethyl-9H-carbazole-2-carboxylate (***5aa***):* Prepared following general procedure A using DHF **3aa** (89 mg, 0.24 mmol) and Yb(OTf)_3_ (15 mg, 24 μmol) in toluene (2.4 mL). Purification via silica gel column chromatography (1–10% EtOAC/Hexanes, R_f_ = 0.59 in 20% EtOAC/Hexanes) afforded **5aa** as yellow crystal (73 mg, 90%). **^1^H NMR** (700 MHz, CDCl_3_) δ 10.99 (s, 1H), 8.21 (dt, *J* = 8.0, 1.0 Hz, 1H), 7.56 (ddd, *J* = 8.2, 7.0, 1.2 Hz, 1H), 7.46 (d, *J* = 8.3 Hz, 1H), 7.40 (s, 1H), 7.28 (ddd, *J* = 8.0, 7.0, 1.0 Hz, 1H), 4.75 (q, *J* = 8.4 Hz, 2H), 4.25 (s, 3H), 2.80 (s, 3H). **^13^C NMR** (176 MHz, CDCl_3_) δ 169.7, 149.6, 142.5, 128.4, 128.1, 126.8, 124.1, 123.3, 123.0 (q, *J* = 277.7 Hz), 122.8, 119.6, 119.5, 109.1, 105.7, 60.6 (q, J = 36.7 Hz), 32.1, 20.4. **^19^F NMR** (471 MHz, CDCl_3_) δ −73.44 (t, *J* = 8.5 Hz, 3F). **IR** 3057.01 (w), 2963.78 (w), 1670.40 (s), 1278.20 (s), 1234.57 (s), 1157.37 (s), 1127.22 (s). **HMRS (ESI)** *m*/*z*: [M + H]^+^ calc. for C_17_H_14_F_3_NO_3_, 338.0999; Found 338.0998. 

*2,2,2-Trifluoroethyl 1-hydroxy-3,9-dimethyl-9H-carbazole-2-carboxylate (***5ab***):* Prepared following general procedure B using DHF **3ab** (110 mg, 0.287 mmol) and Al(OTf)_3_ (14 mg, 29 μmol) in toluene (2.8 mL). Purification via silica gel column chromatography (1–10% EtOAC/Hexanes, R_f_ = 0.59 in 20% EtOAC/Hexanes) afforded **5ab** as yellow crystal (65 mg, 67%). **^1^H NMR** (400 MHz, CDCl_3_) δ 11.83 (s, 1H), 7.52 (ddd, *J* = 8.3, 7.0, 1.2 Hz, 1H), 7.41–7.37 (m, 2H), 7.23 (ddd, *J* = 7.9, 7.1, 1.0 Hz, 1H), 4.72 (q, *J* = 8.4 Hz, 2H), 4.19 (s, 3H), 2.67 (d, *J* = 0.9 Hz, 3H). **^13^C NMR** (101 MHz, CDCl_3_) δ 171.3, 152.7, 142.8, 130.4, 128.0, 127.3, 123.1 (q, *J* = 277.8 Hz) 121.7, 121.0, 119.2, 114.3, 109.2, 106.3, 60.9 (q, *J* = 36.8 Hz), 32.0, 24.5. **IR** 3030.13 (w), 2961.61 (m), 2937.58 (m), 1649.38 (s), 1637.42 (s), 1316.84 (s), 1272.05 (s), 1158.44 (s), 1036.63 (s). **HMRS (ESI)** *m*/*z*: [M + H]^+^ calc. for C_17_H_14_F_3_NO_3_, 338.0999; Found 338.0998. 

*2,2,2-Trifluoroethyl 3-ethyl-1-hydroxy-9-methyl-9H-carbazole-2-carboxylate (***5ac***):* Prepared following general procedure B using DHF **3ac** (108 mg, 0.272 mmol) and Al(OTf)_3_ (13 mg, 27 μmol) in toluene (2.7 mL). Purification via silica gel column chromatography (1–10% EtOAC/Hexanes, R_f_ = 0.59 in 20% EtOAC/Hexanes) afforded **5ac** as yellow crystal (59 mg, 62%). **^1^H NMR** (500 MHz, CDCl_3_) δ 11.80 (s, 1H), 8.03 (dt, *J* = 7.8, 1.0 Hz, 1H), 7.53 (ddd, *J* = 8.3, 7.1, 1.2 Hz, 1H), 7.44 (s, 1H), 7.40 (dt, *J* = 8.4, 0.9 Hz, 1H), 7.24 (ddd, *J* = 7.9, 7.1, 0.9 Hz, 1H), 4.74 (q, *J* = 8.4 Hz, 2H), 4.19 (s, 3H), 3.07 (q, *J* = 7.4 Hz, 2H), 1.29 (t, *J* = 7.4 Hz, 3H). **^13^C NMR** (126 MHz, CDCl_3_) δ 171.1, 152.6, 142.8, 136.9, 128.1, 127.3 (d, J = 2.3 Hz), 123.0 (q, *J* = 277.2 Hz), 121.8, 121.0, 119.2, 113.1, 109.2, 105.7, 61.0 (q, *J* = 36.8 Hz), 32.0, 30.1, 16.8. **IR** 3026.24 (w), 2954.17 (m), 2870.75 (w), 1649.94 (s), 1315.64 (s), 1259.43 (s), 1158.68 (s), 1123.55 (s), 1036.82 (m). **HMRS (ESI)** *m*/*z*: [M + H]^+^ calc. for C_18_H_16_F_3_NO_3_, 352.1155; Found 352.1153.

*2,2,2-Trifluoroethyl 1-hydroxy-9-methyl-3-phenyl-9H-carbazole-2-carboxylate (***5ad***):* Prepared following general procedure B using DHF **3ad** (100 mg, 0.232 mmol) and Al(OTf)_3_ (11 mg, 23 μmol) in toluene (2.3 mL). Purification via silica gel column chromatography (1–10% EtOAC/Hexanes, R_f_ = 0.59 in 20% EtOAC/Hexanes) afforded **5ad** as yellow crystal (60 mg, 65%). **^1^H NMR** (500 MHz, CDCl_3_) δ 11.38 (s, 1H), 8.02 (dt, *J* = 7.9, 1.0 Hz, 1H), 7.56 (ddd, *J* = 8.3, 7.0, 1.2 Hz, 1H), 7.51 (s, 1H), 7.46–7.33 (m, 6H), 7.26 (ddd, *J* = 7.9, 6.6, 0.9 Hz, 1H), 4.38 (q, *J* = 8.4 Hz, 2H), 4.26 (s, 3H). **^13^C NMR** (126 MHz, CDCl_3_) δ 170.7, 151.2, 143.5, 142.8, 135.0, 128.4, 127.7, 127.4, 126.4, 122.2 (q, *J* = 277.5 Hz), 122.0, 121.1, 119.6, 114.9, 109.3, 106.1, 60.6 (q, *J* = 37.2 Hz), 32.1. **IR** 3022.72 (w), 2916.53 (w), 1655.22 (s), 1321.40 (s), 1281.60 (m), 1159.52 (s), 1030.67 (m). **HMRS (ESI)** *m*/*z*: [M + H]^+^ calc. for C_22_H_16_F_3_NO_3_, 400.1155; Found 400.1154.

*2,2,2-Trifluoroethyl 1-hydroxy-9-methyl-9H-carbazole-2-carboxylate (***5ae***):* Prepared following general procedure A using DHF **3ae** (150 mg, 0.406 mmol) and Yb(OTf)_3_ (25 mg, 41 μmol) in toluene (4 mL). Purification via silica gel column chromatography (1–10% EtOAC/Hexanes, R_f_ = 0.59 in 20% EtOAC/Hexanes) afforded **5ae** as yellow crystal (106 mg, 81%). **^1^H NMR** (500 MHz, CDCl_3_) δ 11.15 (s, 1H), 8.07 (dt, *J* = 7.8, 1.0 Hz, 1H), 7.65 (d, *J* = 8.4 Hz, 1H), 7.60–7.51 (m, 2H), 7.43 (dt, *J* = 8.4, 0.9 Hz, 1H), 7.26 (ddd, *J* = 7.9, 7.1, 0.9 Hz, 1H), 4.75 (q, *J* = 8.3 Hz, 2H), 4.23 (s, 3H). **^13^C NMR** (126 MHz, CDCl_3_) δ 169.7, 151.2, 142.4, 129.4, 128.3, 127.4, 123.0 (q, *J* = 277.3 Hz), 122.0, 121.1, 119.5, 119.5, 111.5, 109.2, 106.2, 60.6 (q, *J* = 36.9 Hz), 32.1. **IR** 3061.58 (w), 2964.60 (m), 2933.49 (w), 1668.50 (s), 1630.09 (m), 1464.87 (s), 1250.61 (s), 1144.95 (s), 1040.08 (m). **HMRS (ESI)** *m*/*z*: [M + H]^+^ calc. for C_16_H_12_F_3_NO_3_, 324.0842; Found 324.0840. 

*Reaction of dihydrofuran***3ah***with Al(OTf)_3_:* Following the general procedure B with dihydrofuran **3ah**, Al(OTf)_3_, and no added, an inseparable mixture of carbazole **5ah** and furan **4ah** was formed. Since product **5ah** and **4ah** run at similar R_f_ value, q-NMR was applied to quantify the yield of each one. According to quantitative **^1^H-NMR** (with DMF as internal standard), the respective yield of **5ah** and **4ah** in the mixture were 19% and 68%.

*2,2,2-Trifluoroethyl 1-hydroxy-9-methyl-4-phenyl-9H-carbazole-2-carboxylate (***5ai***):* To a dry flask charged with a stir bar and the mixture of **3ai** and **5ai** prepared in previous section, dry 1,2-DCE (6 mL) was added, followed by addition of Cu(hfacac)_2_ (59 mg, 123 μmol, 20 mol%.). The solution was then heated to 80 °C for 24 h, diluted with Et_2_O (30 mL) and washed with saturated thiourea (20 mL). The organic layer was dried over Na_2_SO_4_, concentrated under reduced pressure, and purified by column chromatography (silica gel, 0–5% EtOAC/Hexanes), which afforded **5ai** as pale-yellow crystal (88 mg, 36% over two steps). **^1^H NMR** (500 MHz, CDCl_3_) δ 11.18 (s, 1H), 7.57–7.46 (m, 7H), 7.43 (dt, *J* = 8.4, 0.9 Hz, 1H), 7.35 (dt, *J* = 8.1, 1.0 Hz, 1H), 6.99 (ddd, *J* = 8.1, 6.9, 1.2 Hz, 1H), 4.75 (q, *J* = 8.4 Hz, 2H), 4.30 (s, 3H). **^13^C NMR** (126 MHz, CDCl_3_) δ 169.7, 150.5, 142.7, 140.3, 129.4, 129.1, 128.6, 128.5, 127.6, 127.1, 127.0, 123.1, 122.9 (q, *J* = 277.4 Hz), 121.8, 120.2, 119.2, 109.1, 105.9, 60.6 (q, *J* = 37.2 Hz), 32.2. **IR** 3056.30 (w), 2946.60 (w), 1658.71 (s), 1465.88 (m), 1236.68 (s), 1234.71 (s) 1155.21 (s), 1128.94 (m). **HMRS (ESI)** *m*/*z*: [M-H]^−^ calc. for C_22_H_16_F_3_NO_3_, 398.1010; Found 398.1004. 

*11-Hydroxy-10-methyl-4,10-dihydropyrano[3,4-b]carbazol-1(3H)-one (***6aj***):* Prepared following general procedure B using DHF **3aj** (88 mg, 0.24 mmol) and Al(OTf)_3_ (11 mg, 24 μmol) in toluene (2.4 mL) without addition of water. Purification via silica gel column chromatography (1–10% EtOAC/Hexanes, R_f_ = 0.38 in 20% EtOAC/Hexanes) afforded **5ae** as yellow crystal (50 mg, 78%). **^1^H NMR** (500 MHz, CDCl_3_) δ 12.01 (s, 1H), 8.02 (dt, *J* = 7.8, 1.0 Hz, 1H), 7.54 (ddd, *J* = 8.3, 7.1, 1.2 Hz, 1H), 7.42 (dt, *J* = 8.4, 0.9 Hz, 1H), 7.36 (t, *J* = 1.1 Hz, 1H), 7.24 (ddd, *J* = 8.0, 7.0, 1.0 Hz, 1H), 4.62 (dd, *J* = 6.4, 5.6 Hz, 2H), 4.21 (s, 3H), 3.19 (ddd, *J* = 6.6, 5.5, 1.1 Hz, 2H). **^13^C NMR** (126 MHz, CDCl_3_) δ 171.3, 151.5, 142.6, 128.7, 128.4, 127.5, 121.8, 121.0, 119.4, 109.3, 109.0, 104.0, 68.8, 31.9, 28.1. **IR** 3056.02 (w), 2914.82 (m), 1648.69 (s), 1468.19 (m), 1302.74 (s), 1253.14 (s), 1124.21 (s). **HMRS (ESI)** *m*/*z*: [M + H]^+^ calc. for C_16_H_13_NO_3_, 268.0968; Found 268.0968. 

*2,2,2-Trifluoroethyl 1-hydroxy-3-(3-hydroxypropyl)-9-methyl-9H-carbazole-2-carboxylate (***5ak***):* Prepared following general procedure B using DHF **3ak** (75 mg, 0.24 mmol) and Al(OTf)_3_ (9 mg, 20 μmol) in toluene (2 mL) without addition of water. Purification via silica gel column chromatography (10–30% EtOAC/Hexanes, R_f_ = 0.14 in 20% EtOAC/Hexanes) afforded **5ak** as yellow crystal (25 mg, 33%). **^1^H NMR** (500 MHz, CDCl_3_) δ 11.81 (s, 1H), 8.03 (dt, *J* = 7.8, 1.0 Hz, 1H), 7.53 (ddd, *J* = 8.3, 7.0, 1.2 Hz, 1H), 7.46 (s, 1H), 7.41 (dt, *J* = 8.4, 0.9 Hz, 1H), 7.24 (ddd, *J* = 7.9, 7.0, 0.9 Hz, 1H), 4.78 (q, *J* = 8.4 Hz, 2H), 4.21 (s, 3H), 3.72 (t, *J* = 6.4 Hz, 2H), 3.16–3.12 (m, 2H), 1.95–1.88 (m, 2H), 1.49 (s, br, 1H). **^13^C NMR** (126 MHz, CDCl_3_) δ 171.0, 152.9, 142.8, 134.1, 128.1, 127.5, 127.4, 123.1 (q, *J* = 277.3 Hz), 121.7, 121.1, 119.3, 114.1, 109.2, 105.7, 62.4, 61.0 (q, *J* = 36.8 Hz), 35.2, 33.3, 32.1. **IR** 3324.04 (w, br), 2958.85 (m), 2925.22 (m), 1647.07 (s), 1434.70 (m), 1313.12 (s), 1256.98 (s), 1157.55 (s), 1035.10 (s). **HMRS (ESI)** *m*/*z*: [M + H]^+^ calc. for C_19_H_18_F_3_NO_4_, 382.1261; Found 382.1259. 

*11-Hydroxy-5,10-dimethyl-4,10-dihydropyrano[3,4-b]carbazol-1(3H)-one (***6al***) and 2,2,2-Trifluoroethyl 1-hydroxy-4-(3-hydroxypropyl)-9-methyl-9H-carbazole-2-carboxylate (***5am***):* To a vial charged with a stir bar and a 2.85:1 mixture of **3al** and **3am** (100 mg, 0.262 mmol) in toluene (2.5 mL) was added Al(OTf)_3_ (12 mg, 26.2 μmol). The suspension was set under sonication for 1 min, followed by stirred and heated under 70 °C for 1 h. Then, another portion of Al(OTf)_3_ (12 mg, 26.2 μmol) was added to the reaction followed by increasing temperature to 85 °C for additional 1 h. After consuming all DHF, the mixture was concentrated under reduced pressure, and purified by column chromatography.

**6al***:* Purification on a silica gel column chromatography (1–10% EtOAC/Hexanes, R_f_ = 0.38 in 20% EtOAC/Hexanes) afforded 5al as yellow crystal (45 mg, 61%). **^1^H NMR** (400 MHz, CDCl_3_) δ 12.06 (s, 1H), 8.24 (d, *J* = 8.1 Hz, 1H), 7.55 (ddd, *J* = 8.3, 7.0, 1.1 Hz, 2H), 7.44 (dt, *J* = 8.3, 0.9 Hz, 2H), 7.25 (ddd, *J* = 8.1, 7.1, 1.1 Hz, 1H), 4.60 (dd, *J* = 6.5, 5.6 Hz, 3H), 4.22 (s, 5H), 3.15 (t, *J* = 6.1 Hz, 3H), 2.69 (s, 5H). **^13^C NMR** (101 MHz, CDCl_3_) δ 171.8, 150.0, 142.7, 127.9, 127.4, 126.8, 125.4, 123.5, 122.7, 119.6, 119.3, 109.1, 103.9, 68.3, 32.0, 24.9, 15.6. **IR** 2994.64 (w), 2922.00 (m), 2851.13 (w), 1654.70 (s), 1626.95 (m), 1317.00 (s), 1224. 95 (s), 1158. 46 (m), 1134.11 (m). HMRS (ESI) *m*/*z*: [M + H]^+^ calc. for C_17_H_15_NO_3_, 282.1125; Found 282.1118.

**5am**: Purification via silica gel column chromatography (10–30% EtOAC/Hexanes, R_f_ = 0.14 in 20% EtOAC/Hexanes) afforded **5am** as pale-yellow crystal (19 mg, 19%). **^1^H NMR** (500 MHz, CDCl_3_) δ 11.03 (s, 1H), 8.20 (dt, *J* = 8.1, 0.9 Hz, 1H), 7.56 (ddd, *J* = 8.2, 7.0, 1.2 Hz, 1H), 7.47 (dt, *J* = 8.3, 0.9 Hz, 1H), 7.43 (s, 1H), 7.28 (ddd, *J* = 8.1, 7.1, 1.1 Hz, 1H), 4.76 (q, *J* = 8.3 Hz, 2H), 4.26 (s, 3H), 3.84 (t, *J* = 6.3 Hz, 2H), 3.30–3.21 (m, 2H), 2.13–2.05 (m, 2H). 1.41 (s, br, 1H). **^13^C NMR** (126 MHz, CDCl_3_) δ 169.6, 149.8, 142.6, 128.8, 128.1, 127.4, 126.8, 123.3, 123.0 (q, *J* = 277.5 Hz), 121.9, 119.7, 118.8, 109.2, 105.8, 62.6, 60.6 (q, *J* = 37.0 Hz), 32.5, 32.1, 30.0. **IR** 3325.97 (s, br), 2931.32 (m), 2891.86 (w), 1662.28 (s), 1332.21 (s), 1278.97 (s), 1239.36 (s), 1153.52(s), 1040.43 (s). **HMRS (ESI)** *m*/*z*: [M + H]^+^ calc. for C_19_H_18_F_3_NO_4_, 382.1261; Found 382.1258.

*2,2,2-Trifluoroethyl 9-benzyl-1-hydroxy-4-methyl-9H-carbazole-2-carboxylate (***5ba***):* Prepared following general procedure A using DHF **3ba** (175 mg, 0.393 mmol) and Yb(OTf)_3_ (24 mg, 39 μmol) in toluene (3.9 mL). Purification via silica gel column chromatography (1–10% EtOAC/Hexanes, R_f_ = 0.59 in 20% EtOAC/Hexanes) afforded **5ba** as yellow crystal (133 mg, 82%). **^1^H NMR** (500 MHz, CDCl_3_) δ 11.36 (s, 1H), 8.58 (dt, *J* = 8.0, 0.9 Hz, 1H), 7.84 (ddd, *J* = 8.2, 7.0, 1.2 Hz, 1H), 7.80–7.74 (m, 2H), 7.67–7.52 (m, 4H), 7.53–7.47 (m, 2H), 6.30 (s, 2H), 5.07 (q, *J* = 8.3 Hz, 2H), 3.16 (s, 3H). **^13^C NMR** (126 MHz, CDCl_3_) δ 169.5, 149.20, 142.0, 138.6, 128.5, 128.3, 127.9, 127.1, 126.9, 126.3, 124.0, 123.3, 123.04, 122.97 (q, *J* = 277.1 Hz), 119.9, 119.8, 109.8, 105.9, 60.6 (q, *J* = 36.8 Hz), 48.5, 20.4. **IR** 2969.19 (w), 2918.79 (w), 1664.19 (s), 1459.67 (s), 1302.85 (m), 1156.55 (s), 1122.03 (s). **HMRS (ESI)** *m*/*z*: [M + H]^+^ calc. for C_23_H_18_F_3_NO_3_, 414.1311; Found 414.1313.

*2,2,2-Trifluoroethyl 9-benzyl-1-hydroxy-3-methyl-9H-carbazole-2-carboxylate (***5bb***):* Prepared following general procedure B using DHF **3bb** (624 mg, 1.36 mmol) and Al(OTf)_3_ (64 mg, 136 μmol) in toluene (14 mL) with couple drops of water. Purification on a silica gel column chromatography (1–10% EtOAC/Hexanes, R_f_ = 0.59 in 20% EtOAC/Hexanes) afforded 5bb as yellow crystal (376 mg, 67%). **^1^H NMR** (500 MHz, CDCl_3_) δ 11.88 (s, 1H), 8.06 (d, J = 7.8 Hz, 1H), 7.49–7.45 (m, 2H), 7.38 (d, J = 8.3 Hz, 1H), 7.26–7.18 (m, 4H), 7.17–7.13 (m, 2H), 5.93 (s, 2H), 4.74 (q, J = 8.3 Hz, 2H), 2.71 (s, 3H). 13C NMR (126 MHz, CDCl_3_) δ 171.3, 152.5, 142.4, 138.8, 130.9, 128.5, 128.4, 127.5, 127.1, 126.9, 126.4, 123.0 (q, *J* = 277.4 Hz), 122.0, 121.1, 119.6, 114.4, 109.9, 106.7, 60.9 (q, *J* = 37.0 Hz), 48.5, 24.6. **IR** 3030.93 (w), 2974.09 (w), 2936.23 (m), 1645.01 (s), 1435.07 (s), 1314.23 (s), 1251.83 (s), 1150.59 (s), 1038.06 (s). **HMRS (ESI)** *m*/*z*: [M + H]^+^ calc. for C_23_H_18_F_3_NO_3_, 414.1311; Found 414.1313.

**E. Synthesis of benzyl-protected Murrayafoline A (9) from Carbazole 5bb**: The conversion from **5bb** to murrayafoline A was inspired by a reported literature procedure [57]. Wet DMSO (10 mL) was added to a vial charged with a stir bar and **5bb** (310 mg, 0.75 mmol). The mixture was heating at 120 °C for overnight (~18 h), poured into 40 mL brine, extracted with 50 mL EtOAc. The organic layer was washed with brine (2 × 40 mL) and the combined aqueous layer was extracted with another 50 mL EtOAc. The combined organic layer was dried over Na_2_SO_4_, concentrated under reduced pressure, and purified by column chromatography to afford the decarboxylated carbazole product as a yellow solid (151 mg, 70%, with 18% recovery of **5bb**). Next, anhydrous acetone (20 mL) was added to a flask charged with a stir bar, decarboxylated product (151 mg, 0.525 mmol), and K_2_CO_3_ (363 mg, 2.63 mmol, 5 eq.). The mixture was stirred at 0 °C for 20 min, then MeI (373 mg, 2.63 mmol, 5 eq.) was slowly added to cold suspension. The reaction mixture was removed from the ice-bath stirred at room temperature overnight (~18 h). The reaction was concentrated under reduced pressure, re-dissolved in Et_2_O (60 mL), and washed with brine (30 mL). The organic layer was dried over Na_2_SO_4_, concentrated under reduced pressure, and purified by column chromatography to afford *N*-benzyl murrayafoline A (**9**) as a yellow gel (150 mg, 94%). Characterizations were consistent with previously reported literature [48] and ^1^H-NMR spectrum is attached.

## 4. Conclusions

In summary, we have reported a Lewis acid-catalyzed synthesis of substituted 1-hydroxycarbazole-2-carboxylates in yields up to 90%. The approach employs 2,3-dihydrofuran acetals as substrates for an intramolecular ring-opening benzannulation. The dihydrofurans are readily accessed from the Cu(II)-catalyzed reaction of enol ethers and α-diazo-β-indolyl-β-ketoesters. The substituent pattern of the enol ether is ultimately reflected in the carbazole products as 1,1-disubstituted enol ethers afford 4-substituted-1-hydroxycarbazole-2-carboxylates, 1,2-disubstituted enol ethers provide the corresponding 3-substituted-1-hydroxycarbazole derivatives, and a 1,1,2-trisubstituted enol ether gives the 3,4-disubstituted-1-hydroxycarbazole-2-carboxylates. Thus, a modular approach to dihydrofurans allows for strategic synthetic design to access carbazole structural diversity. As an example, a formal synthesis of murrayafoline A, a bioactive natural product, was outlined.

## Figures and Tables

**Table 1:** Acid Screening ^1^.

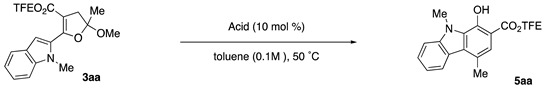		
Entry	Acid	Yield (%) ^2^
1	none	NR ^3^
2	Al(OTf)_3_	79
3	Sc(OTf)_3_	71
4	Ga(OTf)_3_	66
5	Zn(OTf)_2_	11 ^4^
6	Mg(OTf)_2_	NR ^3^
7	Yb(OTf)_3_	90
8	In(OTf)_3_	82
9	Hf(OTf)_4_	79
10	YbCl_3_	24 ^4,5^
11	AlCl_3_	80 ^5^
12	TfOH	66 ^5^
13	*p*TsOH•H_2_O	55 ^5^

^1^ Reaction performed with dihydrofuran acetal **3aa** and indicated acid catalyst (10 mol%) in toluene (0.1 M) at 70 °C for 1 h. ^2^ Isolated yields after column chromatography. ^3^ No Reaction after 12 h. ^4^ Did not go to completion after 12 h. ^5^ Crude ^1^H NMR yield of **5aa** using dimethylsulfone as internal standard.

## Data Availability

The data presented in this study are available within the article or the Supplementary Materials here. Samples of diazo compounds, dihydrofurans, and carbazoles are available from the authors.

## Supplementary Materials

The following supporting information can be downloaded at: https://www.mdpi.com/article/10.3390/molecules27238344/s1, The charts for ^1^H, ^13^C, and representative ^19^F NMRs are available online.

## References

[1] Knölker H.-J., Reddy K.R. (2002). Isolation and Synthesis of Biologically Active Carbazole Alkaloids. Chem. Rev..

[2] Schmidt A.W., Reddy K.R., Knölker H.-J. (2012). Occurrence, Biogenesis, and Synthesis of Biologically Active Carbazole Alkaloids. Chem. Rev..

[3] Issa S., Prandina A., Bedel N., Rongved P., Yous S., Le Borgne M., Bouaziz Z. (2019). Carbazole Scaffolds in Cancer Therapy: A Review from 2012 to 2018. J. Enzyme Inhib. Med. Chem..

[4] Knölker H.-J., Reddy K.R., Cordell G.A. (2008). Chapter 4—Biological and Pharmacological Activities of Carbazole Alkaloids. The Alkaloids.

[5] Bashir M., Bano A., Ijaz A.S., Chaudhary B.A. (2015). Recent Developments and Biological Activities of N-Substituted Carbazole Derivatives: A Review. Molecules.

[6] Głuszyńska A. (2015). Biological Potential of Carbazole Derivatives. Eur. J. Med. Chem..

[7] Caruso A., Ceramella J., Iacopetta D., Saturnino C., Mauro M.V., Bruno R., Aquaro S., Sinicropi M.S. (2019). Carbazole Derivatives as Antiviral Agents: An Overview. Molecules.

[8] Juret P., Heron J.F., Couette J.E., Delozier T., Le Talaer J.Y. (1982). Hydroxy-9-methyl-2-ellipticinium for osseous metastases from breast cancer: A 5 year experience. Cancer Treat. Rep..

[9] Wisler J.W., DeWire S.M., Whalen E.J., Violin J.D., Drake M.T., Ahn S., Shenoy S.K., Lefkowitz R.J. (2007). A unique mechanism of -blocker action: Carvedilol stimulates-arrestin signaling. Proc. Natl. Acad. Sci. USA.

[10] Fox S.M., Johnston S.A. (1997). Use of carprofen for the treatment of pain and inflammation in dogs. J. Am. Vet. Med. Assoc..

[11] Grazulevicius J.V., Strohriegl P., Pielichowski J., Pielichowski K. (2003). Carbazole-containing polymers: Synthesis, properties and applications. Prog. Polym. Sci..

[12] Li J., Grimsdale A.C. (2010). Carbazole-based polymers for organic photovoltaic devices. Chem. Soc. Rev..

[13] Jiang H., Sun J., Zhang J. (2012). A Review on Synthesis of Carbazole-based Chromophores as Organic Light-emitting Materials. Curr. Org. Chem..

[14] Yin J., Ma Y., Li G., Peng M., Lin W. (2020). A versatile small-molecule fluorescence scaffold: Carbazole derivatives for bioimaging. Coord. Chem. Rev..

[15] Aggarwal T., Verma A.K. (2019). Recent Advances in the Synthesis of Carbazoles from Indoles. Org. Biomol. Chem..

[16] Banerjee A., Kundu S., Bhattacharyya A., Sahu S., Maji M.S. (2021). Benzannulation Strategies for the Synthesis of Carbazoles, Indolocarbazoles, Benzocarbazoles, and Carbolines. Org. Chem. Front..

[17] Georgiades S.N., Nicolaou P.G. (2019). Chapter One—Recent Advances in Carbazole Syntheses. Adv. Heterocycl. Chem..

[18] Roy J., Jana A.K., Mal D. (2012). Recent Trends in the Synthesis of Carbazoles: An Update. Tetrahedron.

[19] Yaqub G., Hussain E., Rehman M.A., Mateen B. (2009). Review on advancements in synthesis of carbazoles. Asian J. Chem..

[20] For Pertinent Examples of Carbazole Synthesis (since 2017), See Refs 20–44. SciFinder; Chemical Abstracts Service: Columbus, OH; Research Topic: “Carbazole Synthesis”. https://scifinder.cas.org.

[21] Munoz-Torres M.A., Martinez-Lara F., Solas M., Suarez-Pantiga S., Sanz R. (2022). “Back-to-Front” Indole and Carbazole Synthesis from N,N-Bis-(2-bromoallyl)amines by Carbolithiation Reactions with Gold-Catalysis. Adv. Synth. Catal..

[22] Barrera E., Hernandez-Benitez R.I., Gonzalez-Gonzalez C.A., Escalante C.H., Fuentes-Benites A., Gonzalez-Romero C., Becerra-Martinez E., Delgado F., Tamariz J. (2022). Synthesis of Diarylamines and Methylcarbazoles and Formal Total Synthesis of Alkaloids Ellipticine and Olivacine. Eur. J. Org. Chem..

[23] Campbell E., Taladriz-Sender A., Paisley O.I., Kennedy A.R., Bush J.T., Burley G.A. (2021). A Chemo- and regioselective tandem [3 + 2]heteroannulation strategy for carbazole synthesis: Combining two mechanistically distinct bond-forming processes. ChemRxiv.

[24] Reshma H.P., Emmanuel B.D., Beevi J., Dharan S.S. (2021). Insilico design, synthesis and biological evaluation of novel carbazole derivatives. J. Pharm. Sci. Res..

[25] Merkushev A.A., Makarov A.S., Shpuntov P.M., Abaev V.T., Trushkov I.V., Uchuskin M.G. (2021). Oxidative Rearrangement of 2-(2-Aminobenzyl)furans: Synthesis of Functionalized Indoles and Carbazoles. Eur. J. Org. Chem..

[26] Alavi S., Lin J.-B., Grover H.K. (2021). Copper-Catalyzed Annulation of Indolyl α-Diazocarbonyl Compounds Leads to Structurally Rearranged Carbazoles. Org. Lett..

[27] Points G.L., Beaudry C.M. (2021). Regioselective Synthesis of Substituted Carbazoles, Bicarbazoles, and Clausine C. Org. Lett..

[28] Faltracco M., Damian M., Ruijter E. (2021). Synthesis of Carbazoles and Dihydrocarbazoles by a Divergent Cascade Reaction of Donor–Acceptor Cyclopropanes. Org. Lett..

[29] Wang Y., Jia D., Zeng J., Liu Y., Bu X., Yang X. (2021). Benzocarbazole Synthesis via Visible-Light-Accelerated Rh(III)-Catalyzed C–H Annulation of Aromatic Amines with Bicyclic Alkenes. Org. Lett..

[30] Faltracco M., Ortega-Rosales S., Janssen E., Cioc R.C., Vande Velde C.M.L., Ruijter E. (2021). Synthesis of Carbazoles by a Diverted Bischler–Napieralski Cascade Reaction. Org. Lett..

[31] Hao T., Huang L., Wei Y., Shi M. (2021). Copper-Catalyzed Synthesis of Indolyl Benzo[b]Carbazoles and Their Photoluminescence Property. Org. Lett..

[32] Wu C.-J., Cao W.-X., Chen B., Tung C.-H., Wu L.-Z. (2021). Tandem [2+2] Cycloaddition/Rearrangement toward Carbazoles by Visible-Light Photocatalysis. Org. Lett..

[33] Reddy C.R., Srinivasu E., Sathish P., Subbarao M., Donthiri R.R. (2021). One-Pot Arylative Benzannulation of 2-Carbonyl-3-Propargyl Indoles with Boronic Acids Leading to Arylated Carbazoles. J. Org. Chem..

[34] Guo T., Han L., Wang T., Lei L., Zhang J., Xu D. (2020). Copper-Catalyzed Three-Component Formal [3 + 1 + 2] Benzannulation for Carbazole and Indole Synthesis. J. Org. Chem..

[35] Tian X., Song L., Hashmi A.S.K. (2020). Synthesis of Carbazoles and Related Heterocycles from Sulfilimines by Intramolecular C−H Aminations. Angew. Chem. Int. Ed..

[36] Singh S., Samineni R., Pabbaraja S., Mehta G. (2019). A General Carbazole Synthesis via Stitching of Indole-Ynones with Nitromethanes: Application to Total Synthesis of Carbazomycin A, Calothrixin B, and Staurosporinone. Org. Lett..

[37] Bal A., Maiti S., Mal P. (2018). Iodine(III)-Enabled Distal C-H Functionalization of Biarylsulfonanilides. J. Org. Chem..

[38] Maiti S., Bose A., Mal P. (2018). Oxidative N-Arylation for Carbazole Synthesis by C-C Bond Activation. J. Org. Chem..

[39] Tharra P., Baire B. (2018). Regioselective Cyclization of (Indol-3-yl)pentyn-3-ols as an Approach to (Tetrahydro)carbazoles. Org. Lett..

[40] Shao C., Zhou B., Wu Z., Ji X., Zhang Y. (2018). Synthesis of Carbazoles from 2-Iodobiphenyls by Palladium-Catalyzed C−H Activation and Amination with Diaziridinone. Adv. Synth. Catal..

[41] Gonzalez J.F., Rocchi D., Tejero T., Merino P., Menendez J.C. (2017). One-pot synthesis of functionalized carbazoles via a CAN-catalyzed multicomponent process comprising a C-H activation step. J. Org. Chem..

[42] Alimi I., Remy R., Bochet C.G. (2017). Photochemical C-H Activation: Generation of Indole and Carbazole Libraries, and First Total Synthesis of Clausenawalline D. Eur. J. Org. Chem..

[43] Maiti S., Mal P. (2017). Dehydrogenative Aromatic Ring Fusion for Carbazole Synthesis via C-C/C-N Bond Formation and Alkyl Migration. Org. Lett..

[44] Yan Q., Gin E., Wasinska-Kalwa M., Banwell M.G., Carr P.D. (2017). A Palladium-Catalyzed Ullmann Cross-Coupling/Reductive Cyclization Route to the Carbazole Natural Products 3-Methyl-9H-carbazole, Glycoborine, Glycozoline, Clauszoline K, Mukonine, and Karapinchamine A. J. Org. Chem..

[45] Aponte-Guzmán J., Phun L.H., Cavitt M.A., Taylor J.E., Davy J.C., France S. (2016). Catalytic, Cascade Ring-Opening Benzannulations of 2,3-Dihydrofuran O,O- and N,O-Acetals. Chem. Eur. J..

[46] Guerra Faura G., Nguyen T., France S. (2021). Catalyst-Controlled Chemodivergent Reactions of 2-Pyrrolyl-α-diazo-β-ketoesters and Enol Ethers: Synthesis of 1,2-Dihydrofuran Acetals and Highly Substituted Indoles. J. Org. Chem..

[47] Mal D., Senapati B., Pahari P. (2006). Regioselective synthesis of 1-hydroxycarbazoles via anionic [4 + 2] cycloaddition of furoindolones: A short synthesis of murrayafoline-A. Tetrahedron Lett..

[48] Youn S.W., Kim Y.H., Jo Y.H. (2019). Palladium-Catalyzed Regioselective Synthesis of 1-Hydroxycarbazoles Under Aerobic Conditions. Adv. Synth. Catal..

[49] Padmaja P., Reddy N. (2015). Padmaja Pannala and Reddy Narayana, Hydroxycarbazoles as Starting Materials in Organic Syntheses. Curr. Org. Synth..

[50] Galli C., Mandolini L. (2000). The Role of Ring Strain on the Ease of Ring Closure of Bifunctional Chain Molecules. Eur. J. Org. Chem..

[51] Itoigawa M., Kashiwada Y., Ito C., Furukawa H., Tachibana Y., Bastow K.F., Lee K.-H. (2000). Antitumor Agents. 203. Carbazole Alkaloid Murrayaquinone A and Related Synthetic Carbazolequinones as Cytotoxic Agents. J. Nat. Prod..

[52] Cuong N.M., Wilhelm H., Porzel A., Arnold N., Wessjohann L. (2008). 1-O-Substituted derivatives of murrayafoline A and their antifungal properties. Nat. Prod. Res..

[53] Cui C.-B., Yan S.-Y., Cai B., Yao X.-S. (2002). Carbazole alkaloids as new cell cycle inhibitor and apopTosis inducers from *Clausena dunniana*. J. Asian Nat. Prod. Res..

[54] Choi H., Gwak J., Cho M., Ryu M.-J., Lee J.-H., Kim S.K., Kim Y.H., Lee G.W., Yun M.-Y., Cuong N.M. (2010). Murrayafoline A attenuates the Wnt/β-catenin pathway by promoting the degradation of intracellular β-catenin proteins. Biochem. Biophys. Res. Commun..

[55] Parker A.N., Martin M.C., Shenje R., France S. (2019). Calcium-Catalyzed Formal [5 + 2] Cycloadditions of Alkylidene β-Ketoesters with Olefins: Chemodivergent Synthesis of Highly Functionalized Cyclohepta[b]Indole Derivatives. Org. Lett..

[56] Cuzzupe A.N., Hutton C.A., Lilly M.J., Mann R.K., McRae K.J., Zammit S.C., Rizzacasa M.A. (2001). Total Synthesis of the Epidermal Growth Factor Inhibitor (-)-Reveromycin B. J. Org. Chem..

[57] Evans D.A., Scheidt K.A., Downey C.W. (2001). Synthesis of (-)-Epibatidine. Org. Lett..

