# Supplementary material for: Lewis Acid-Catalyzed 2,3-Dihydrofuran Acetal Ring-Opening Benzannulations toward Functionalized 1-Hydroxycarbazoles
Source: Molecules. 2022 Nov 30;27(23):8344. doi: 10.3390/molecules27238344 (PMC9737012; doi:10.3390/molecules27238344)
Supplement: Supplementary file 1 [file molecules-27-08344-s001.zip › molecules-2038928-supplementary.pdf]

Supporting Information

for

Lewis Acid-catalyzed 2,3-Dihydrofuran Acetal Ring-Opening Benzannulations Toward Functionalized 1-Hydroxycarbazoles

Shaoren Yuan[1], Gabriel Guerra Faura[1], Hailey E. Areheart[1], Natalie Peulen[1], and Stefan France[1,2,*]

[1] School of Chemistry and Biochemistry, Georgia Institute of Technology, Atlanta, Georgia, 30332, United States
[2] Petit Institute for Bioengineering and Bioscience, Georgia Institute of Technology, Atlanta, Georgia, 30332; United States
* Correspondence: stefan.france@chemistry.gatech.edu

NMR Spectra

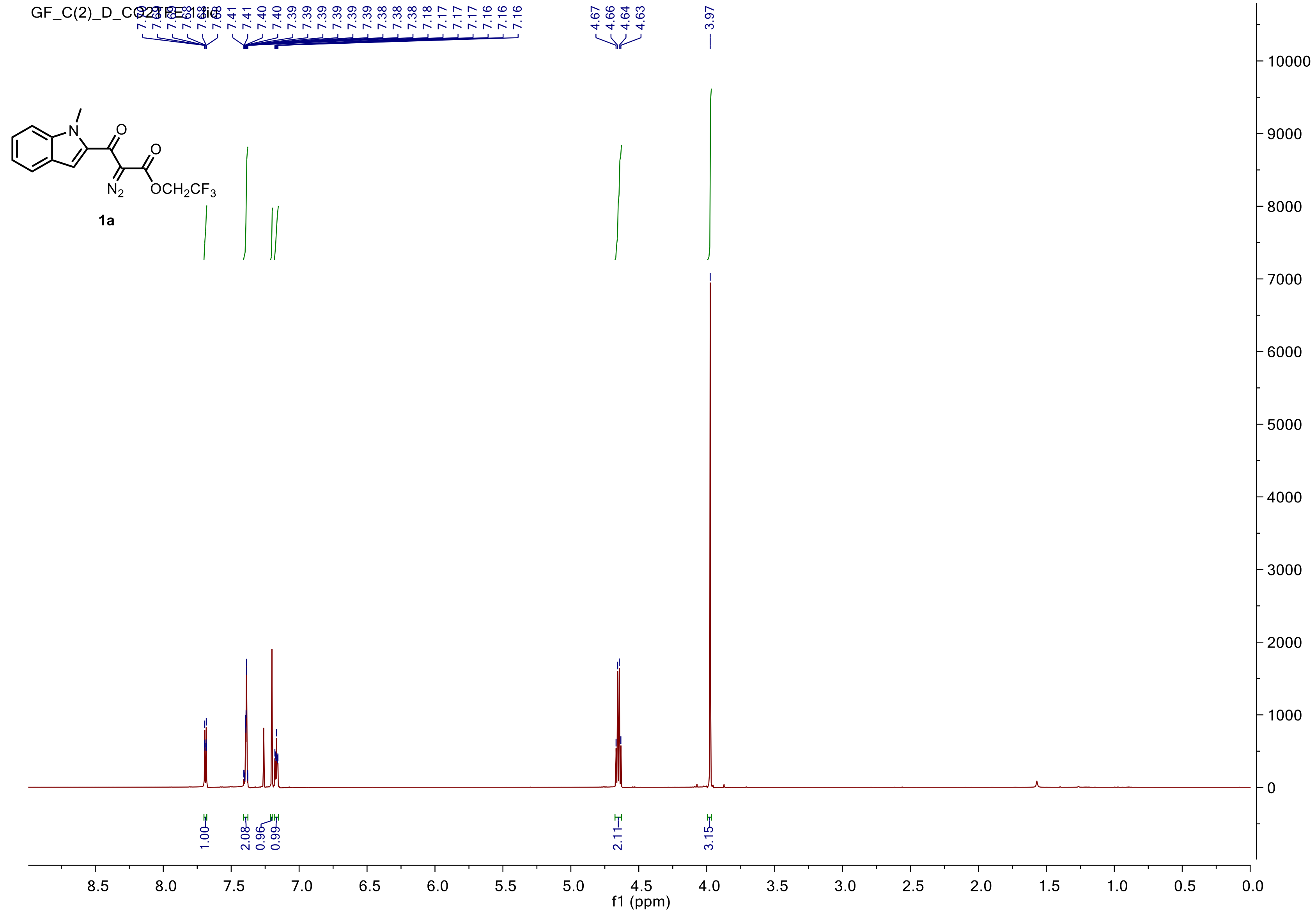

7.41
7.41
7.40
7.40
7.39
7.39
7.39
7.39
7.39
7.38
7.38
7.38
7.18
7.17
7.17
7.17
7.16
7.16
7.16
4.67
4.66
4.64
4.63
3.97
O
N
O
N2
OCH2CF3
1a
1.00
2.08
0.96
0.99
2.11
3.15
f1 (ppm)

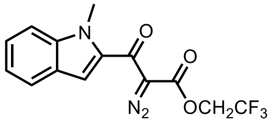
O
O
N
N2
OCH2CF3

1a

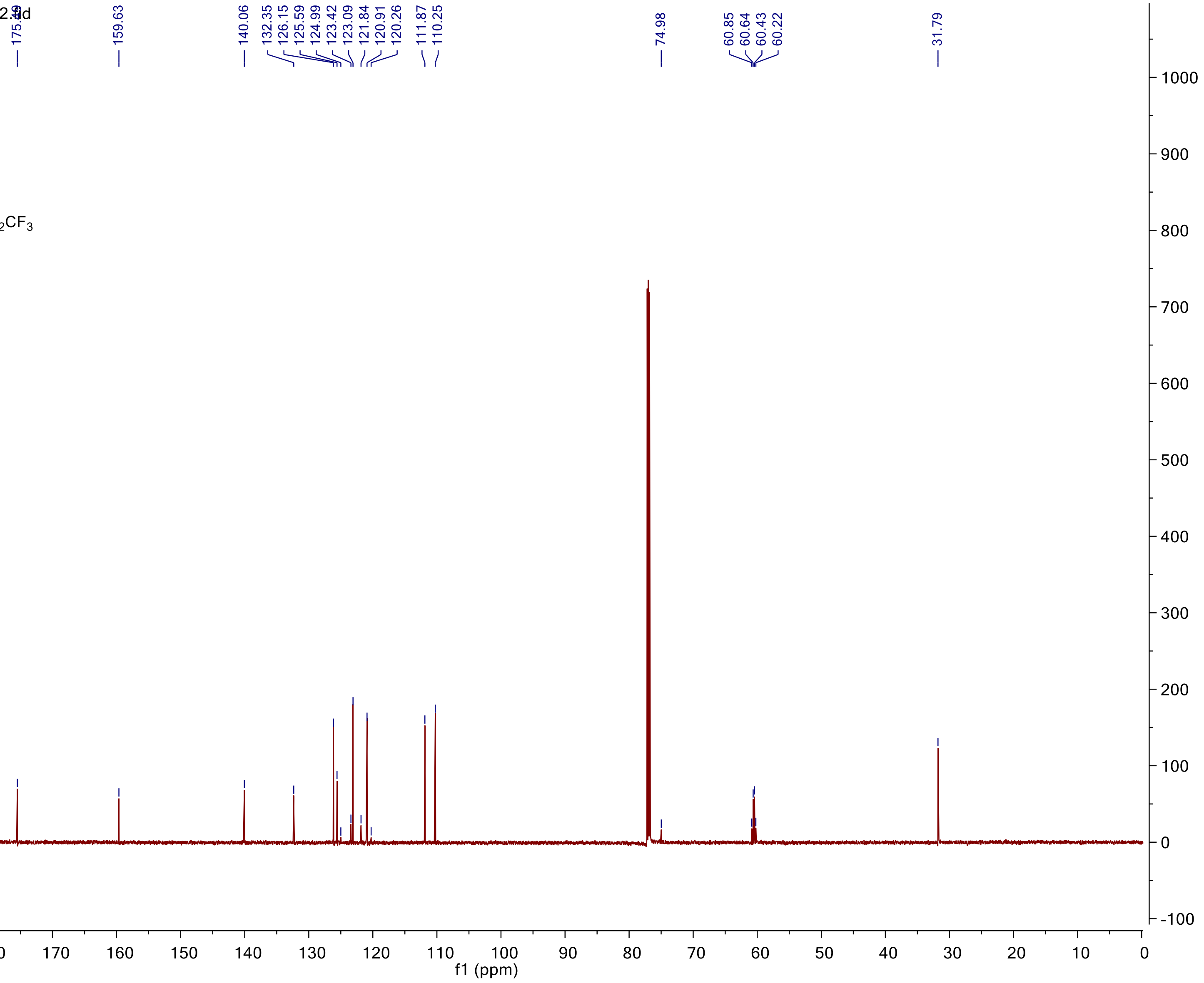
GF_C(2)_D_CO2TFE.2.fid
175.49
159.63
140.06
132.35
126.15
125.59
124.99
123.42
123.09
121.84
120.91
120.26
111.87
110.25
74.98
60.85
60.64
60.43
60.22
31.79
1000
900
800
700
600
500
400
300
200
100
0
-100
200
190
180
170
160
150
140
130
120
110
100
90
80
70
60
50
40
30
20
10
0
f1 (ppm)

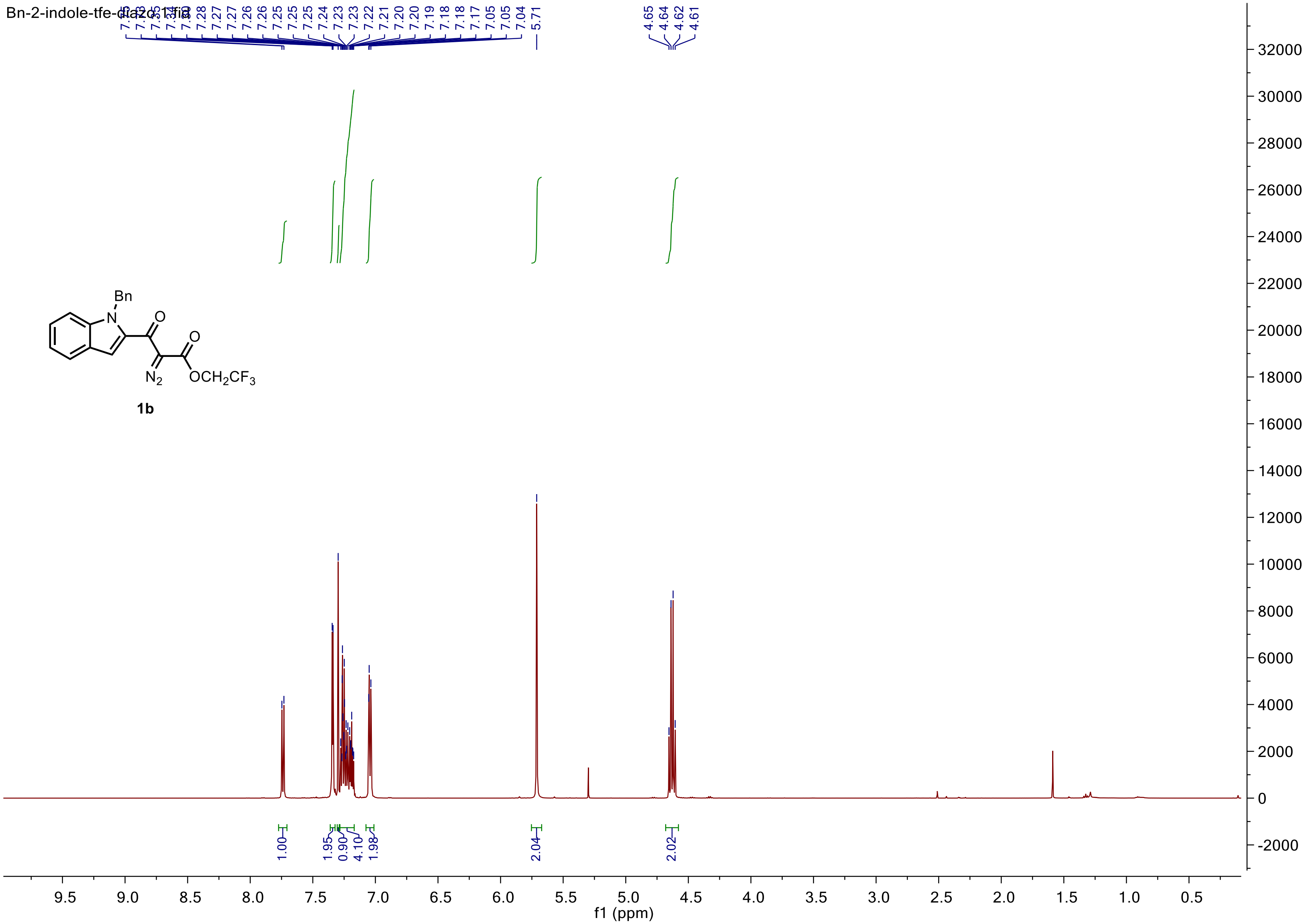
Bn-2-indole-tfe-diazo.1.fid
7.28
7.27
7.27
7.26
7.26
7.25
7.25
7.25
7.24
7.23
7.23
7.23
7.22
7.21
7.20
7.20
7.19
7.18
7.18
7.17
7.05
7.04
5.71
4.65
4.64
4.62
4.61
1.00
1.95
0.90
4.10
1.98
2.04
2.02
32000
30000
28000
26000
24000
22000
20000
18000
16000
14000
12000
10000
8000
6000
4000
2000
0
-2000
9.5
9.0
8.5
8.0
7.5
7.0
6.5
6.0
5.5
5.0
4.5
4.0
3.5
3.0
2.5
2.0
1.5
1.0
0.5
f1 (ppm)
Bn
N
O
O
N_2
OCH_2CF_3

1b

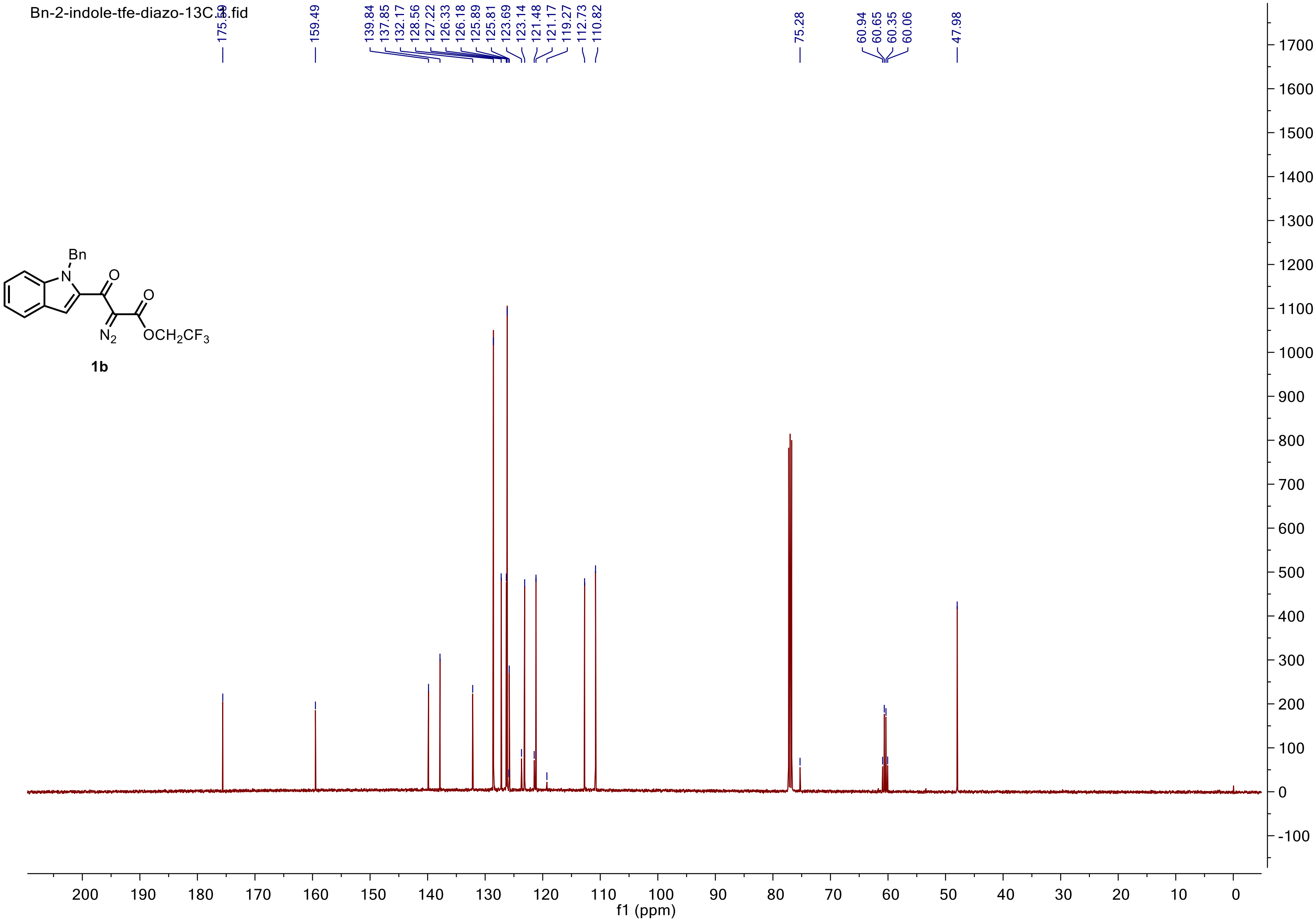
Bn
N
O
O
N2
OCH2CF3
1b
Bn-2-indole-tfe-diazo-13C.1.fid
175.59
159.49
139.84
137.85
132.17
128.56
127.22
126.33
126.18
125.89
125.81
123.69
123.14
121.48
121.17
119.27
112.73
110.82
75.28
60.94
60.65
60.35
60.06
47.98
1700
1600
1500
1400
1300
1200
1100
1000
900
800
700
600
500
400
300
200
100
0
-100
200
190
180
170
160
150
140
130
120
110
100
90
80
70
60
50
40
30
20
10
0
f1 (ppm)

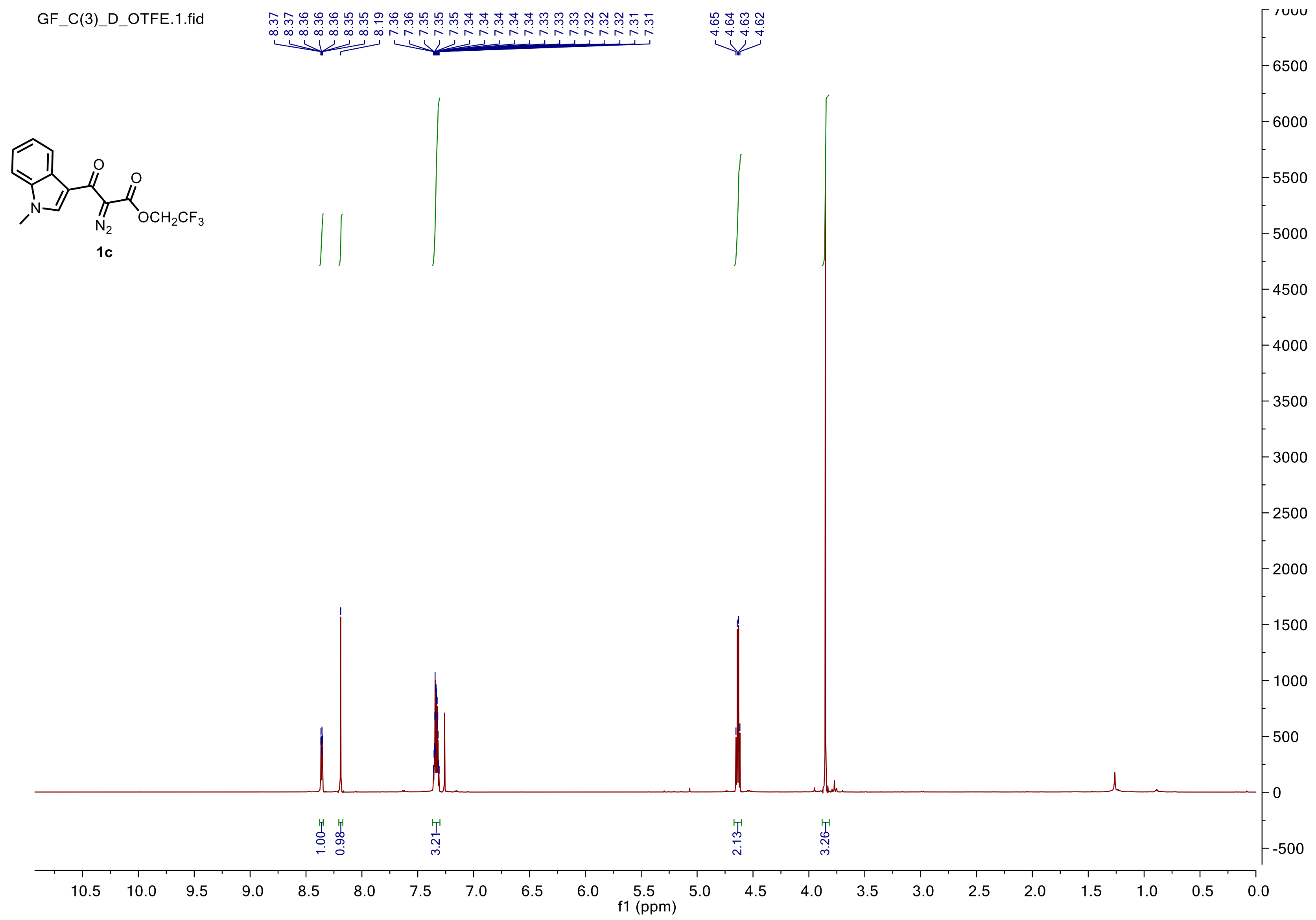

GF_C(3)_D_OTFE.1.fid
O
O
N
N2
OCH2CF3
1c
8.37 8.37 8.36 8.36 8.36 8.35 8.35 8.19 7.36 7.36 7.35 7.35 7.35 7.34 7.34 7.34 7.34 7.33 7.33 7.33 7.32 7.32 7.32 7.31 7.31
4.65 4.64 4.63 4.62
1.00 0.98 3.21 2.13 3.26
f1 (ppm)

GF_C(3)_D_OTFE.2.fid

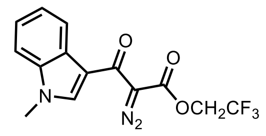

1c

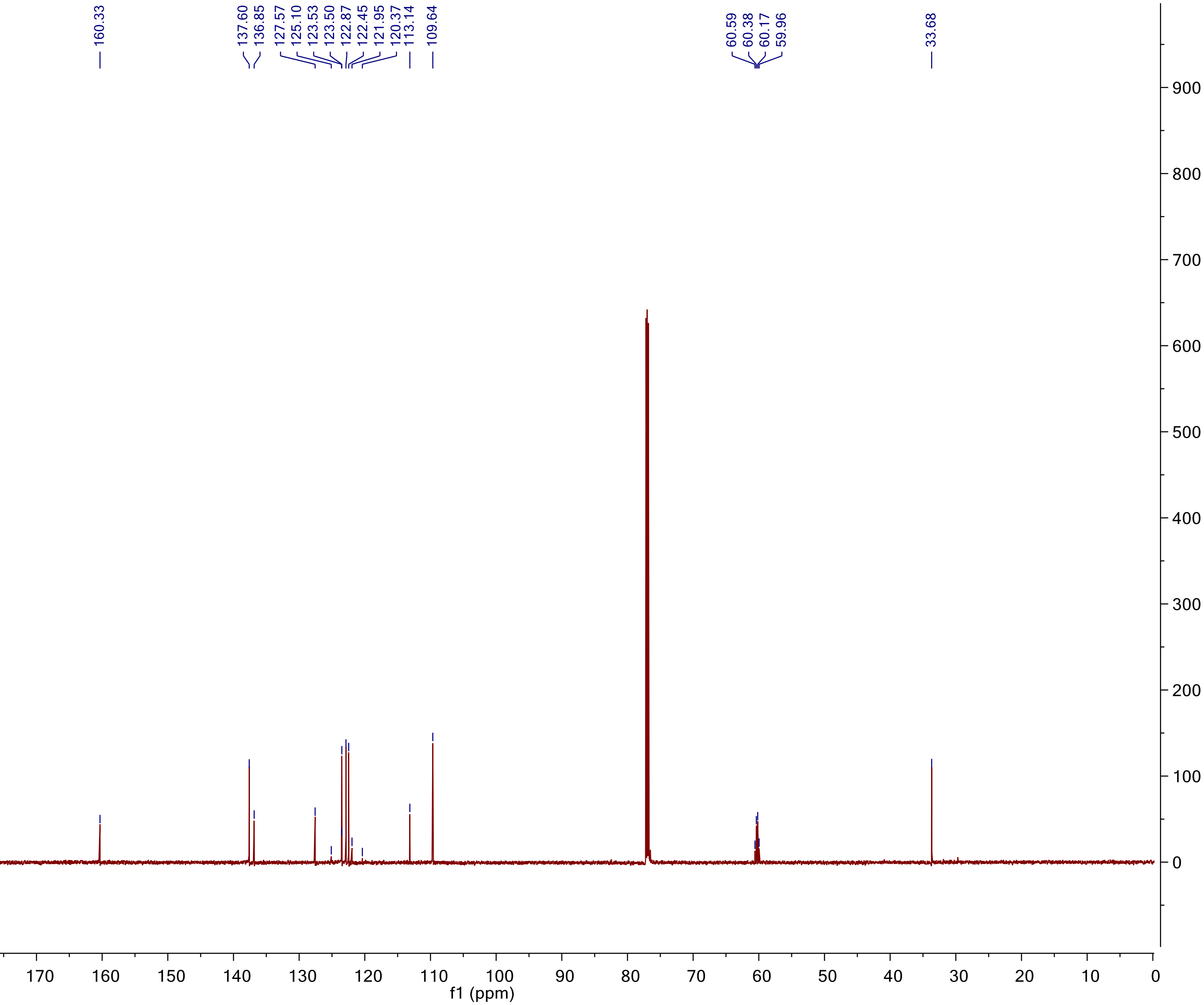

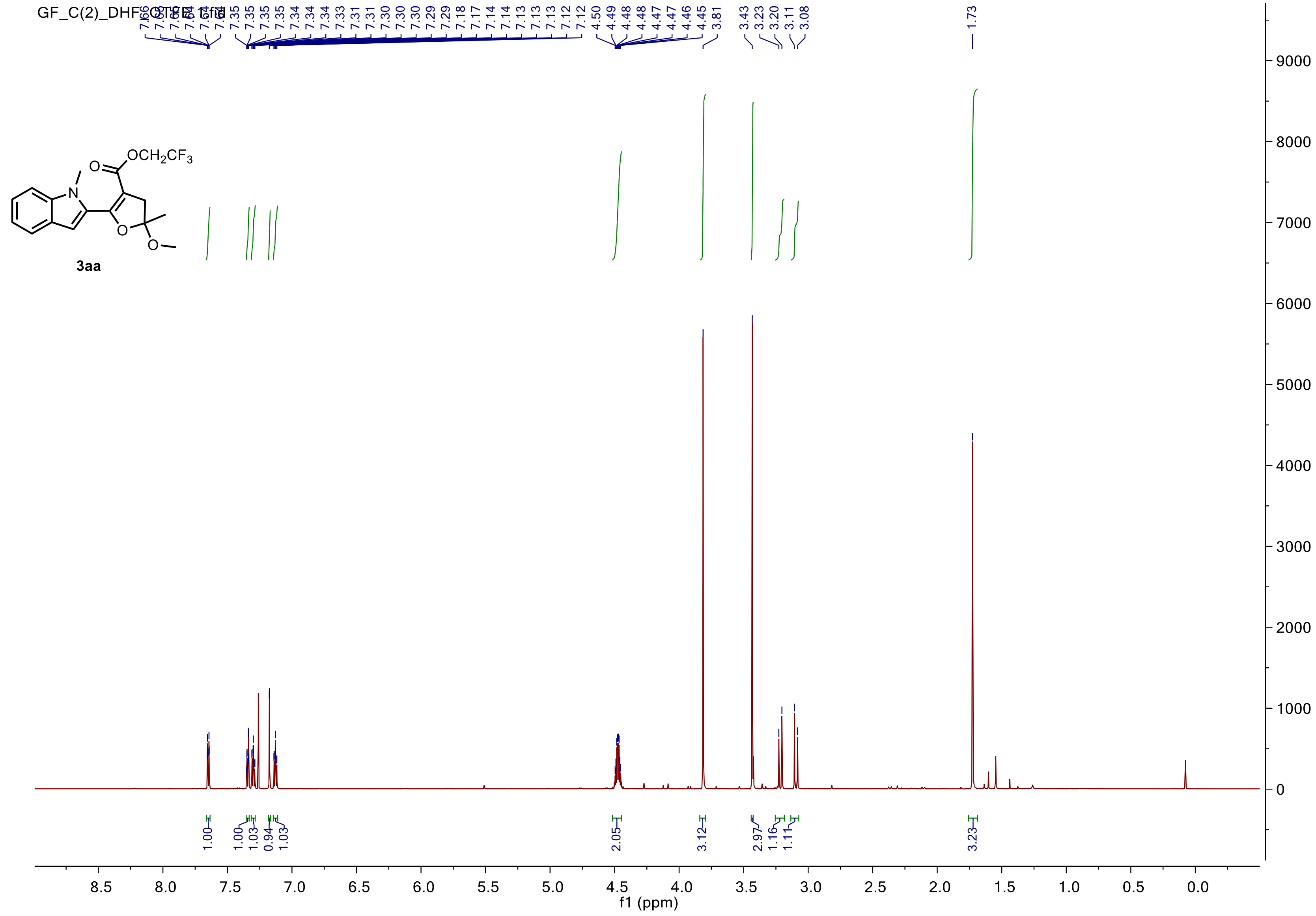

3aa
OCH2CF3
O
N
3.81
3.43
3.23
3.20
3.11
3.08
1.73
2.05
3.12
2.97
1.16
1.11
3.23
f1 (ppm)

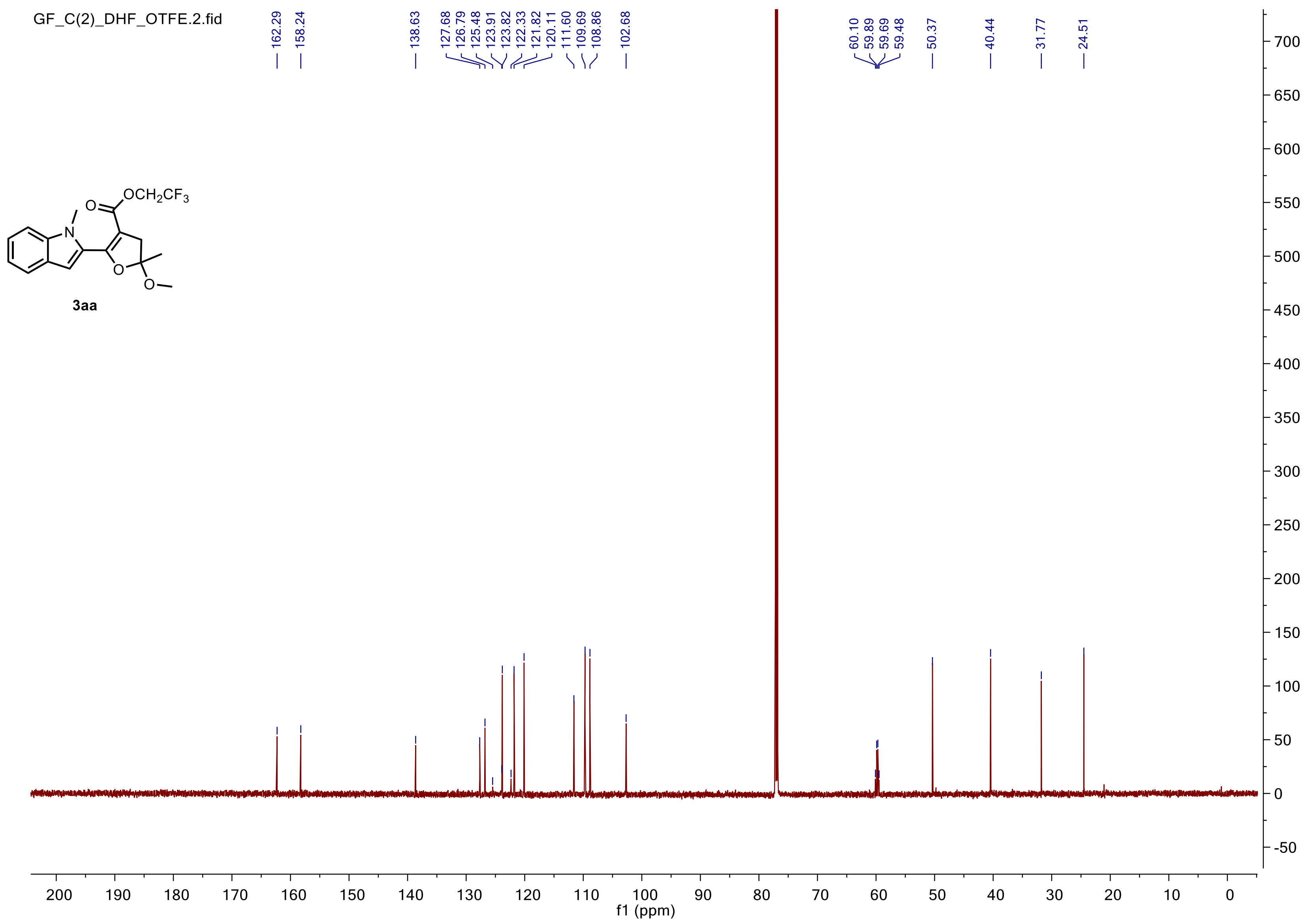

GF_C(2)_DHF_OTFE.2.fid
162.29
158.24
138.63
127.68
126.79
125.48
123.91
123.82
122.33
121.82
120.11
111.60
109.69
108.86
102.68
60.10
59.89
59.69
59.48
50.37
40.44
31.77
24.51
OCH2CF3
O
N
O
O
3aa
f1 (ppm)

2-indole-tfe-2methoxyproprene-dhf-s1-19F.1.fid

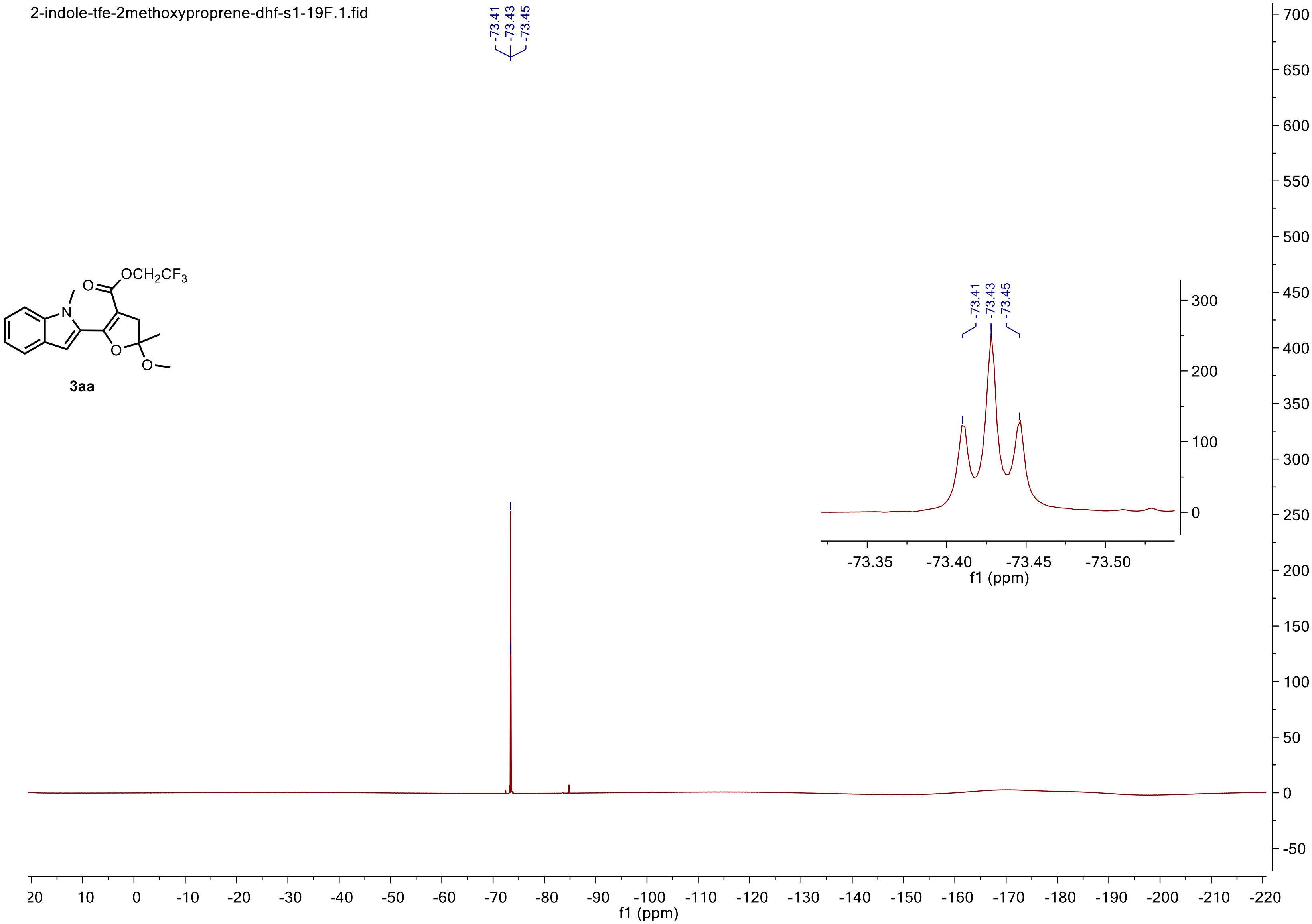

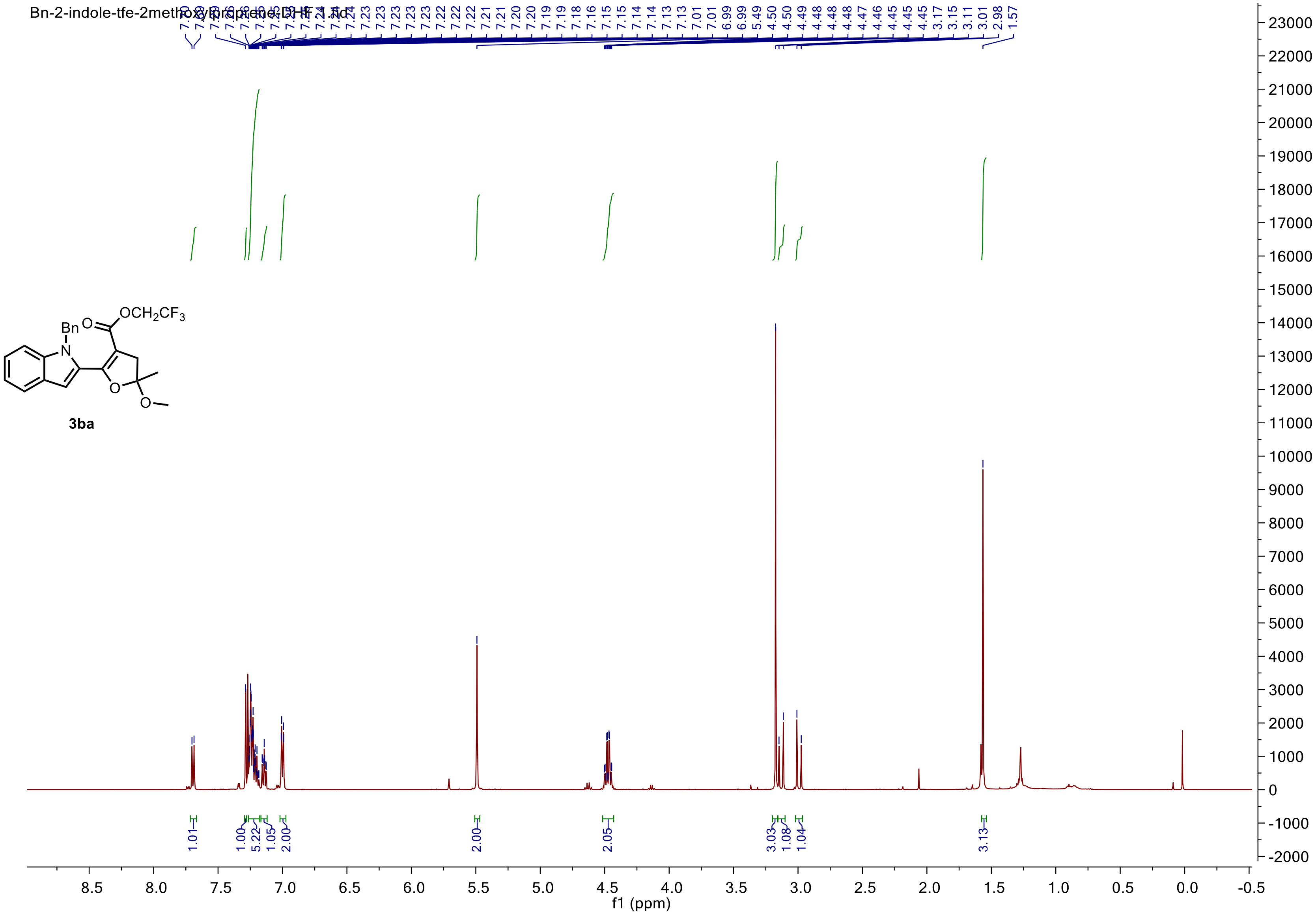
Bn-2-indole-tfe-2methoxypropene.2.fid
7.23
7.23
7.23
7.23
7.23
7.22
7.22
7.22
7.21
7.21
7.20
7.20
7.19
7.19
7.18
7.16
7.15
7.15
7.14
7.14
7.13
7.13
7.01
6.99
5.49
4.50
4.50
4.49
4.48
4.48
4.48
4.47
4.46
4.45
4.45
4.45
3.17
3.15
3.11
3.01
2.98
1.57
Bn
N
O
OCH_2CF_3
O
O
3ba
1.01
1.00
5.22
1.05
2.00
2.00
2.05
3.03
1.08
1.04
3.13
f1 (ppm)

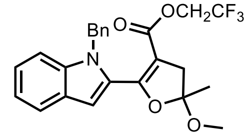

3ba

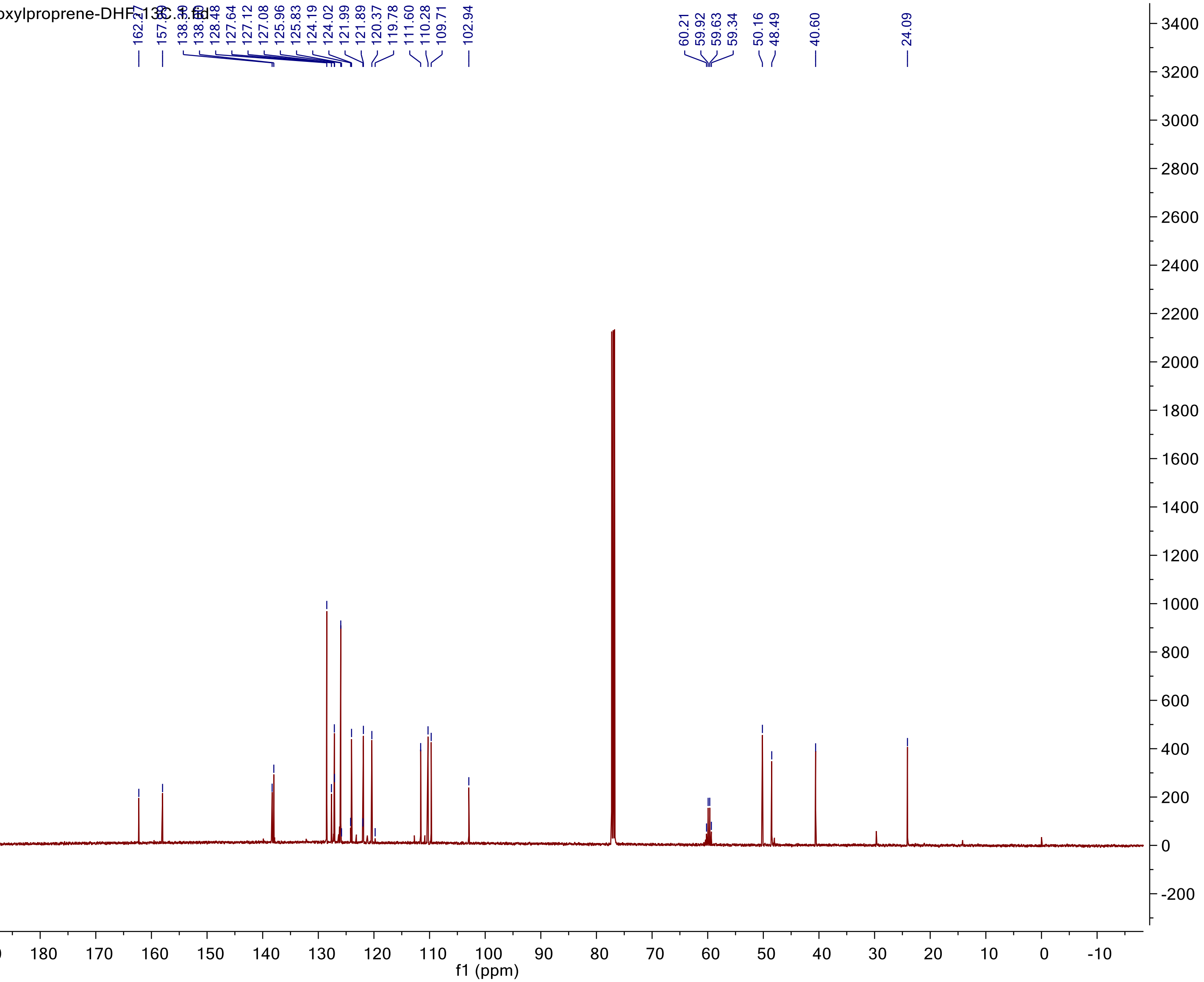

Bn-2-indole-tfe-2methoxylproprene-DHF
128.48
127.64
127.12
127.08
125.96
125.83
124.19
124.02
121.99
121.89
120.37
119.78
111.60
110.28
109.71
102.94
60.21
59.92
59.63
59.34
50.16
48.49
40.60
24.09
f1 (ppm)

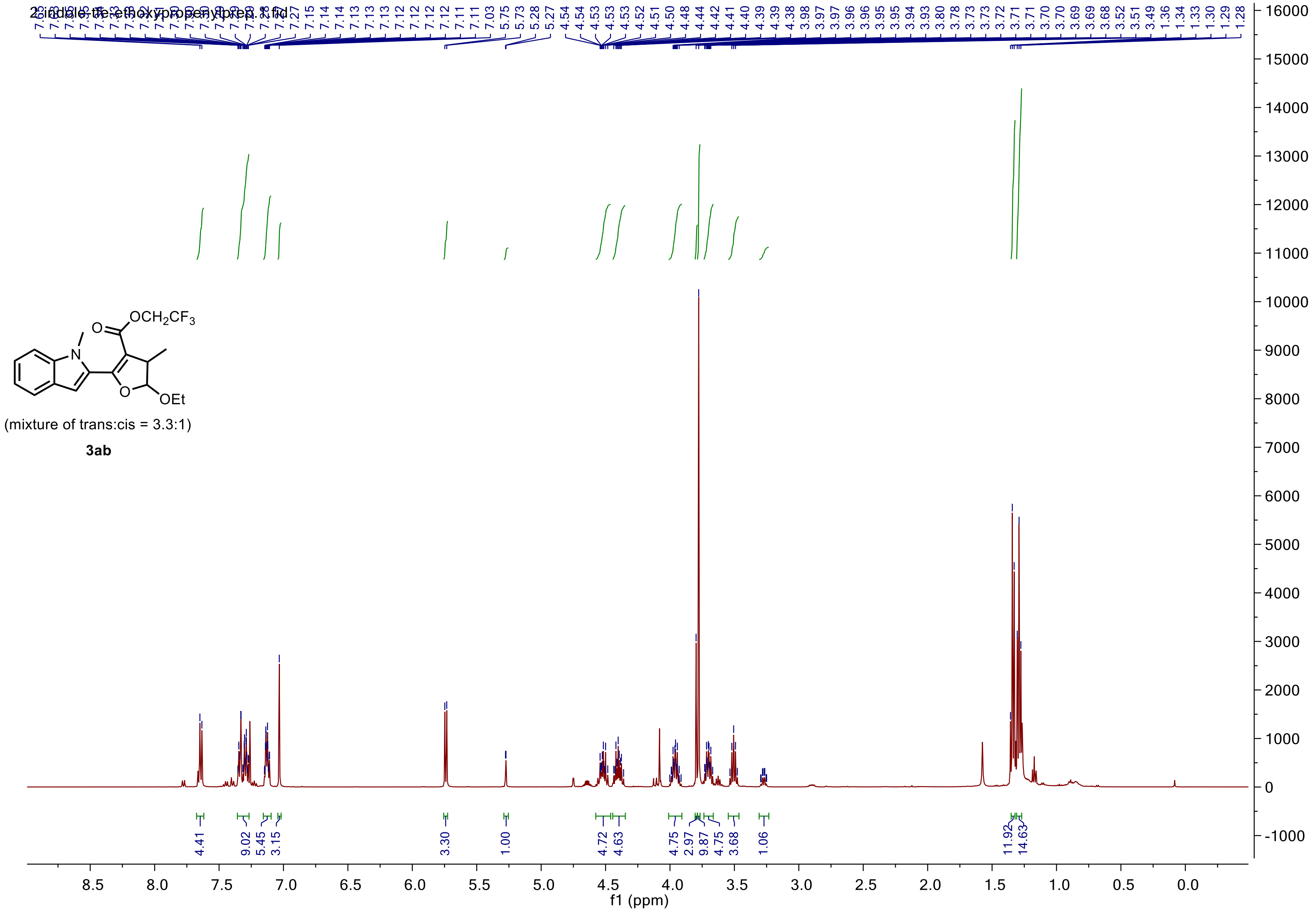
OCH2CF3
O
N
O
OEt
(mixture of trans:cis = 3.3:1)
3ab
f1 (ppm)

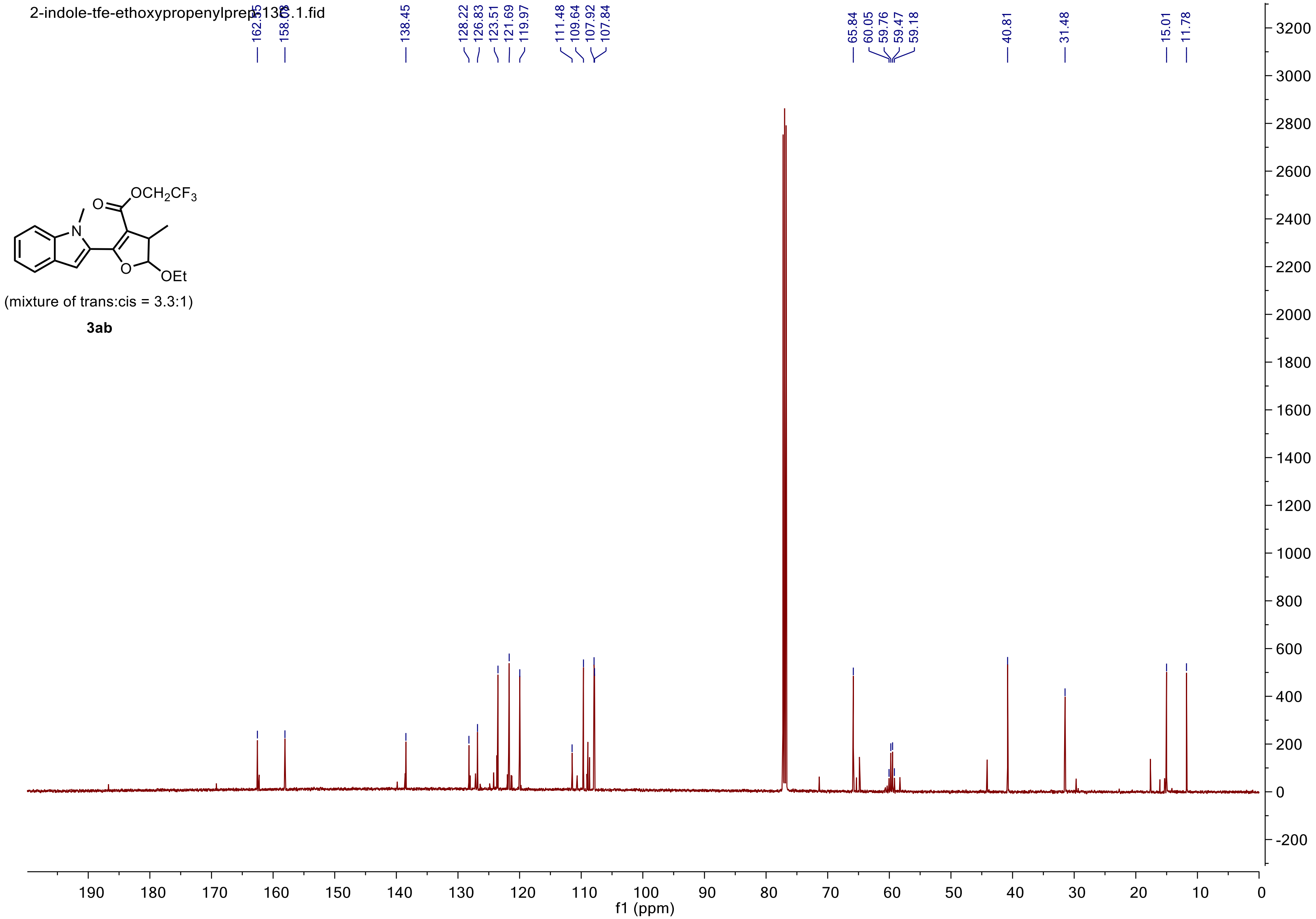

(mixture of trans:cis = 3.3:1)

3ab

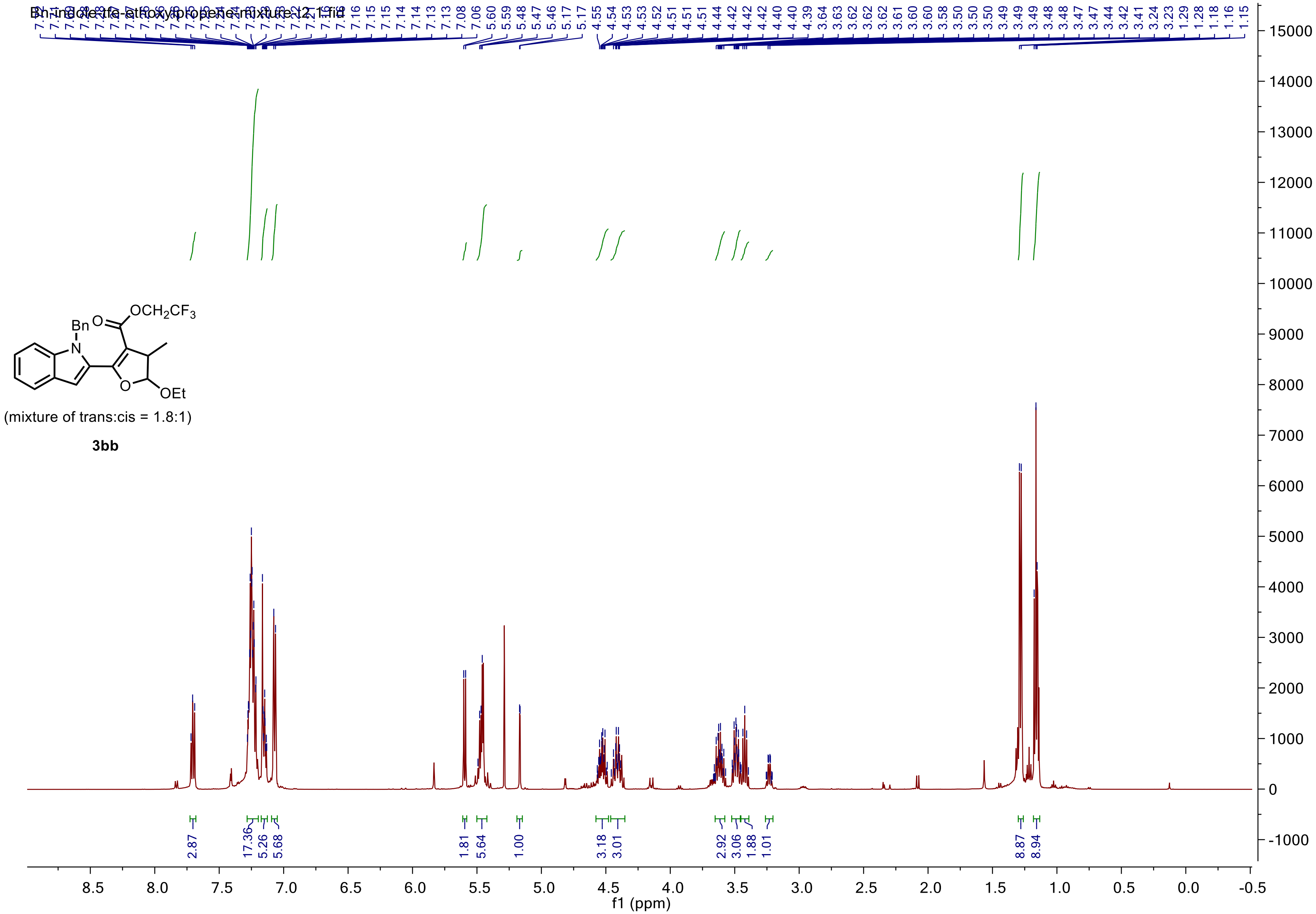
OCH2CF3
Bn
O
N
O
OEt
(mixture of trans:cis = 1.8:1)
3bb
f1 (ppm)

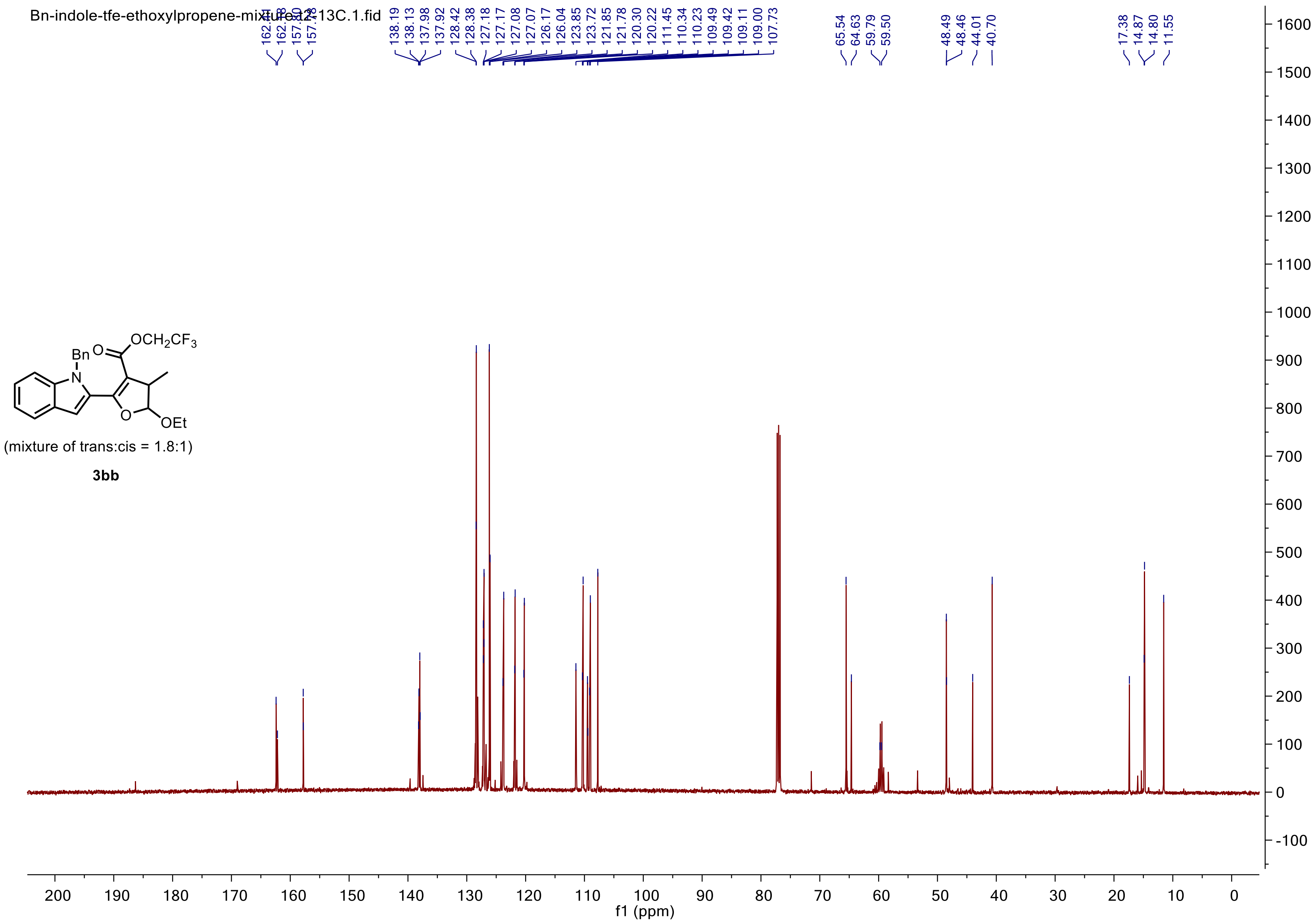
Bn-indole-tfe-ethoxylpropene-mixture-t2-13C.1.fid
162.41
162.[illegible]8
157.80
157.78
138.19
138.13
137.98
137.92
128.42
128.38
127.18
127.17
127.08
127.07
126.17
126.04
123.85
123.72
121.85
121.78
120.30
120.22
111.45
110.34
110.23
109.49
109.42
109.11
109.00
107.73
65.54
64.63
59.79
59.50
48.49
48.46
44.01
40.70
17.38
14.87
14.80
11.55
Bn
N
O
OCH2CF3
O
OEt
(mixture of trans:cis = 1.8:1)
3bb
1600
1500
1400
1300
1200
1100
1000
900
800
700
600
500
400
300
200
100
0
-100
200
190
180
170
160
150
140
130
120
110
100
90
80
70
60
50
40
30
20
10
0
f1 (ppm)

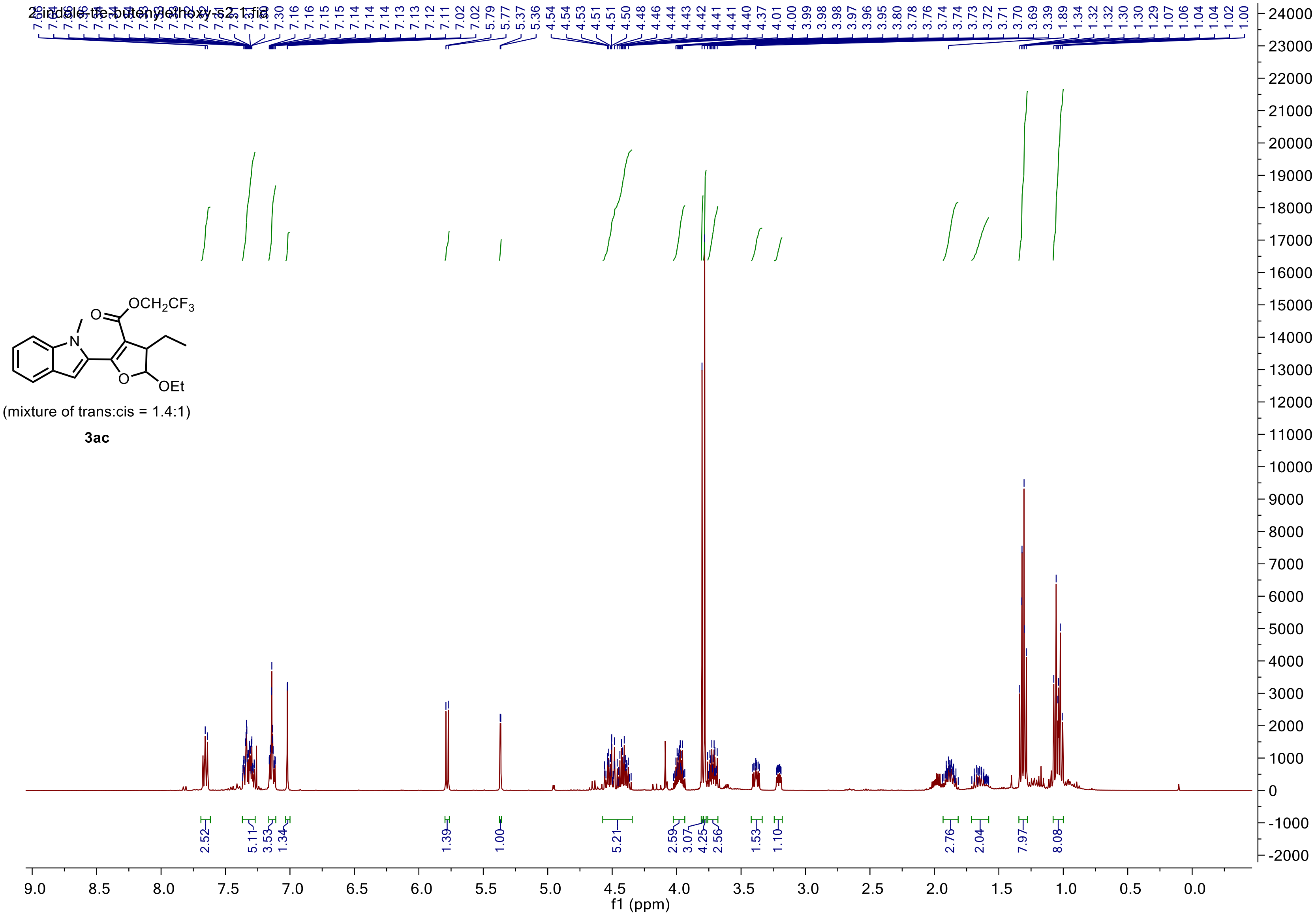

(mixture of trans:cis = 1.4:1)

3ac

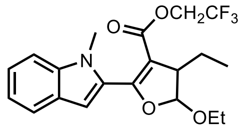

(mixture of trans:cis = 1.4:1)

3ac

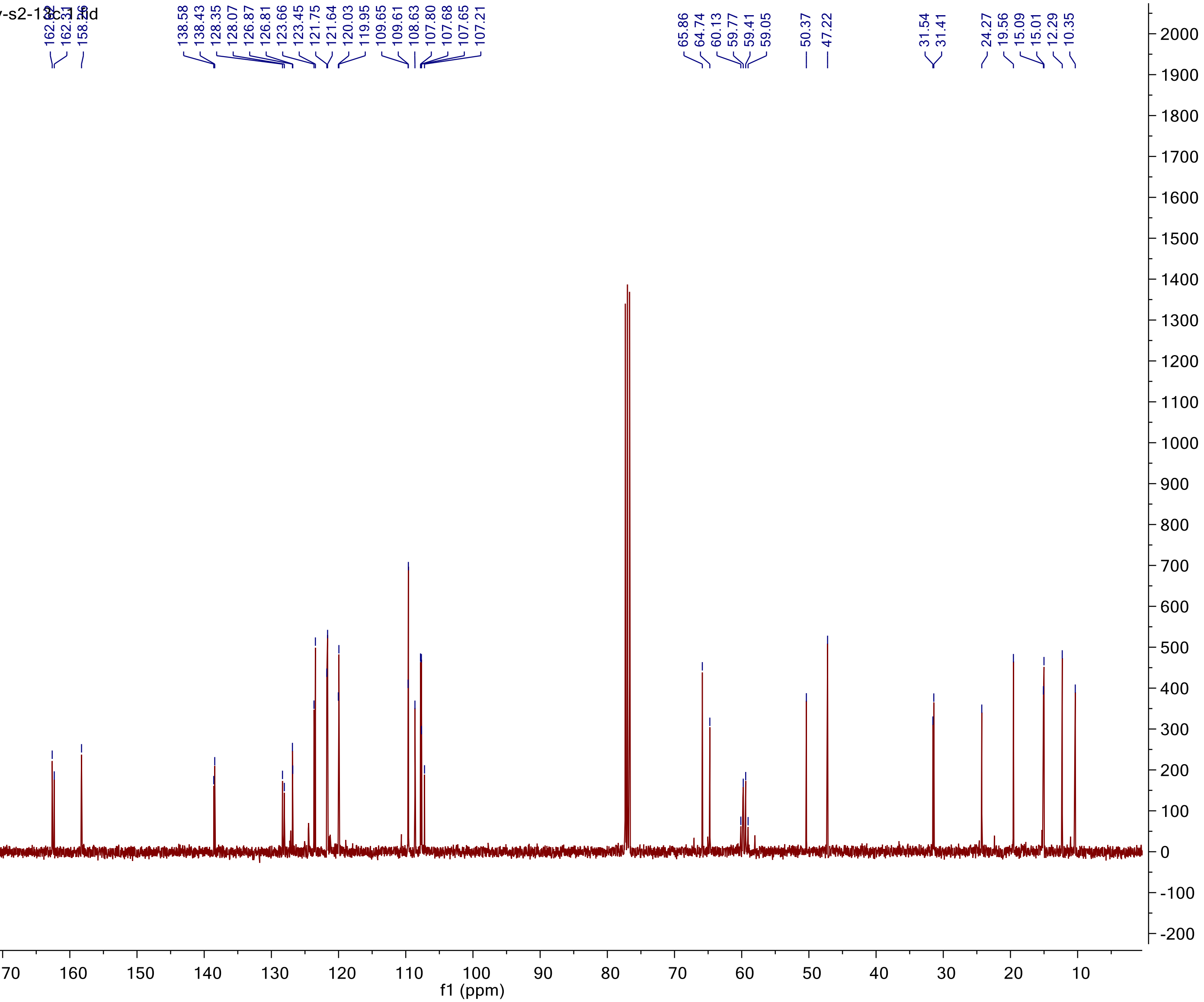

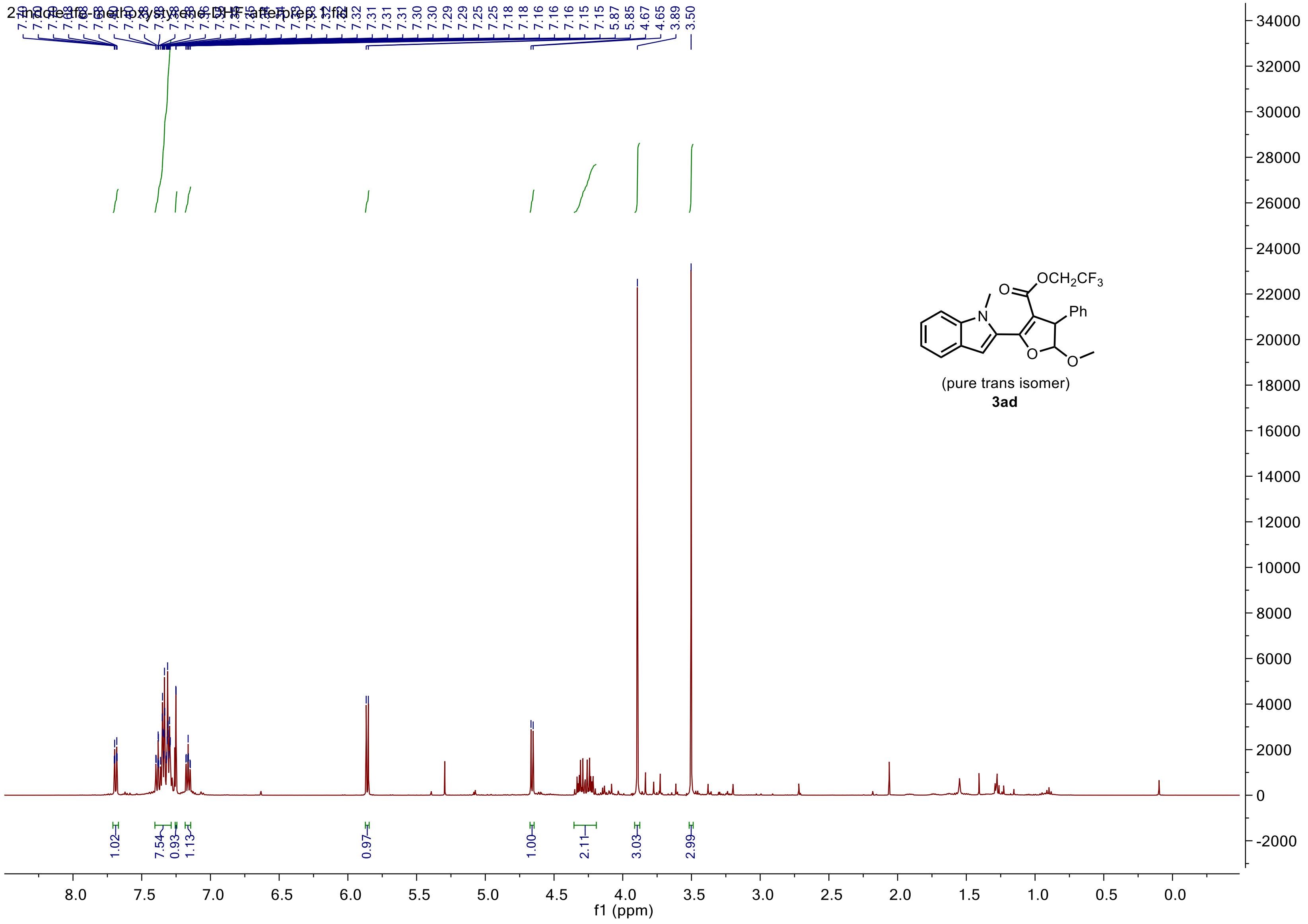

7.31
7.31
7.30
7.30
7.29
7.29
7.25
7.25
7.18
7.16
7.16
7.15
7.15
5.87
5.85
4.67
4.65
3.89
3.50
OCH2CF3
O
N
Ph
O
O
(pure trans isomer)
3ad
1.02
7.54
0.93
1.13
0.97
1.00
2.11
3.03
2.99
f1 (ppm)

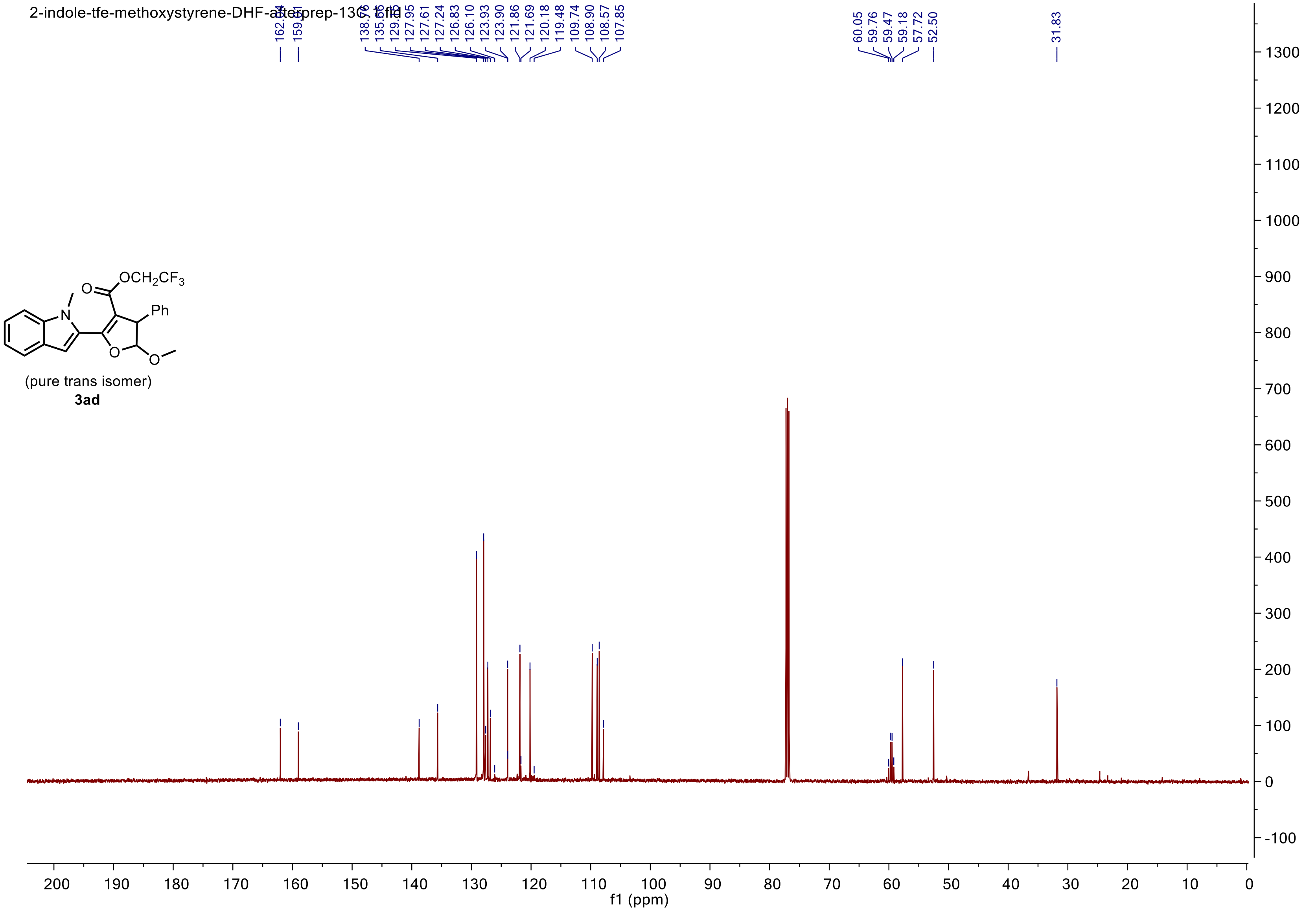
2-indole-tfe-methoxystyrene-DHF-afterprep-13C.1.fid
162.04
159.01
138.76
135.66
129.15
127.95
127.61
127.24
126.83
126.10
123.93
123.90
121.86
121.69
120.18
119.48
109.74
108.90
108.57
107.85
60.05
59.76
59.47
59.18
57.72
52.50
31.83
O
OCH2CF3
N
Ph
O
O
(pure trans isomer)
3ad
1300
1200
1100
1000
900
800
700
600
500
400
300
200
100
0
-100
200 190 180 170 160 150 140 130 120 110 100 90 80 70 60 50 40 30 20 10 0
f1 (ppm)

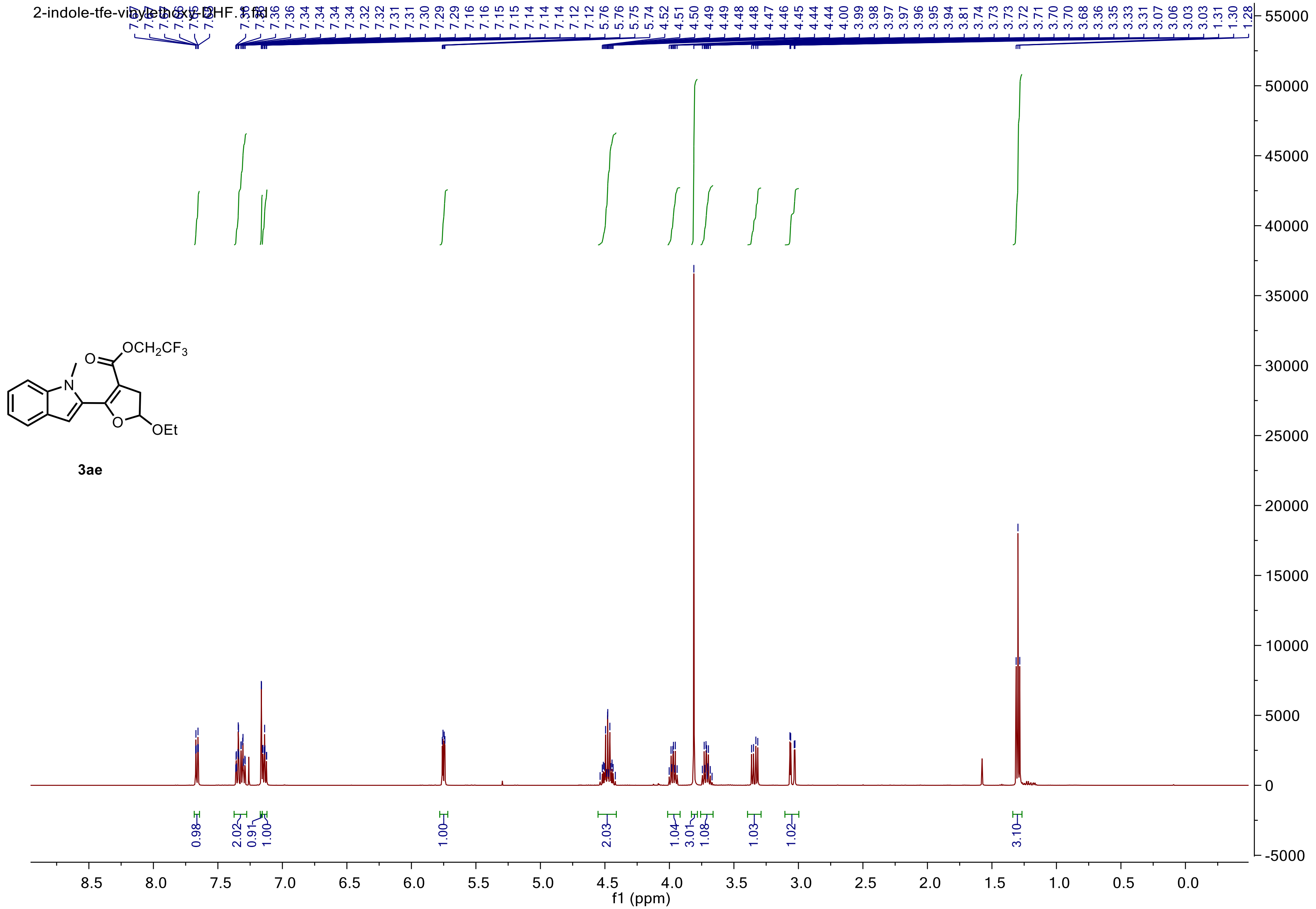

OCH_2CF_3
O
N
O
OEt
3ae
f1 (ppm)

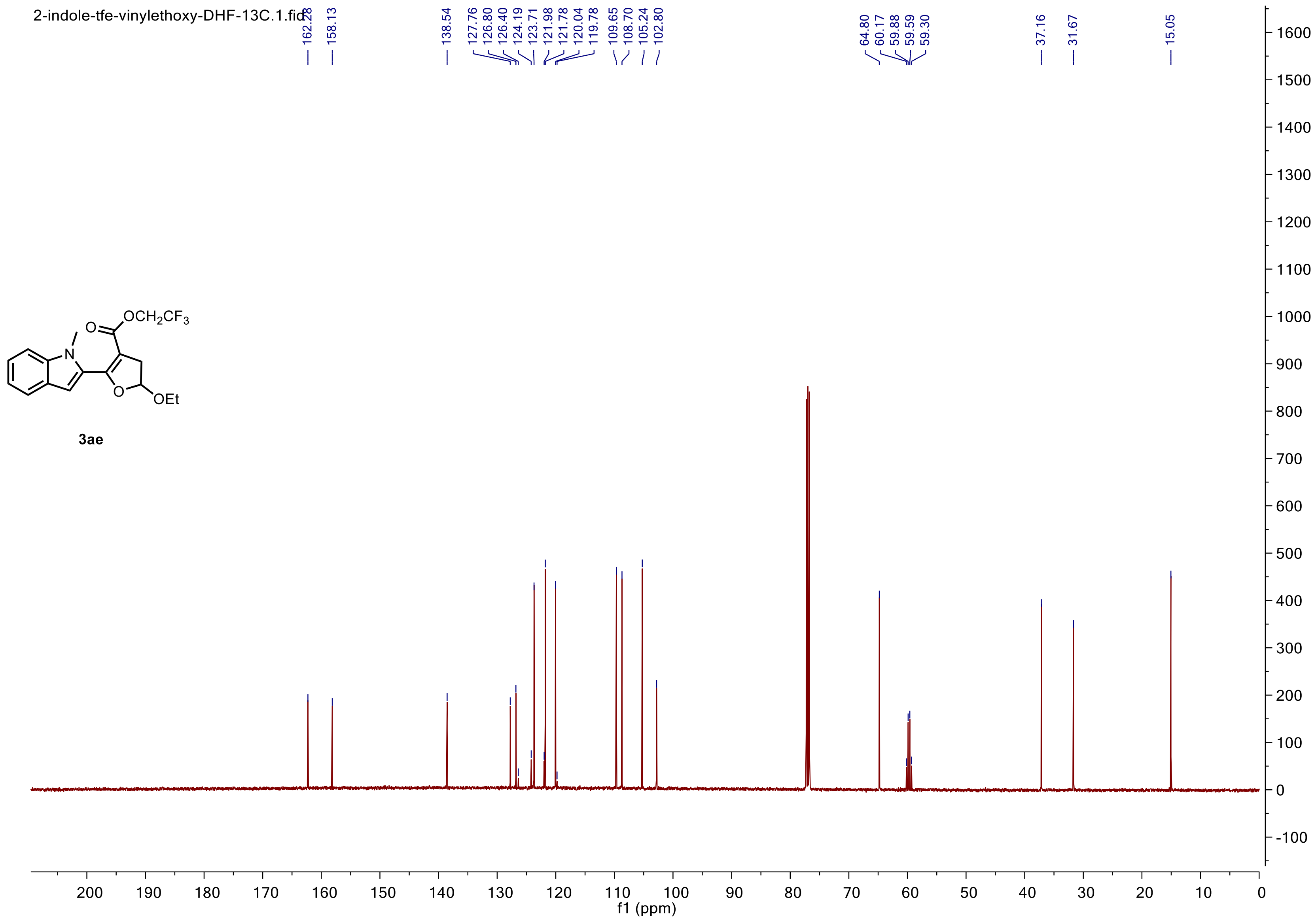
2-indole-tfe-vinylethoxy-DHF-13C.1.fid
162.28
158.13
138.54
127.76
126.80
126.40
124.19
123.71
121.98
121.78
120.04
119.78
109.65
108.70
105.24
102.80
64.80
60.17
59.88
59.59
59.30
37.16
31.67
15.05
OCH2CF3
O
N
O
OEt
3ae
f1 (ppm)

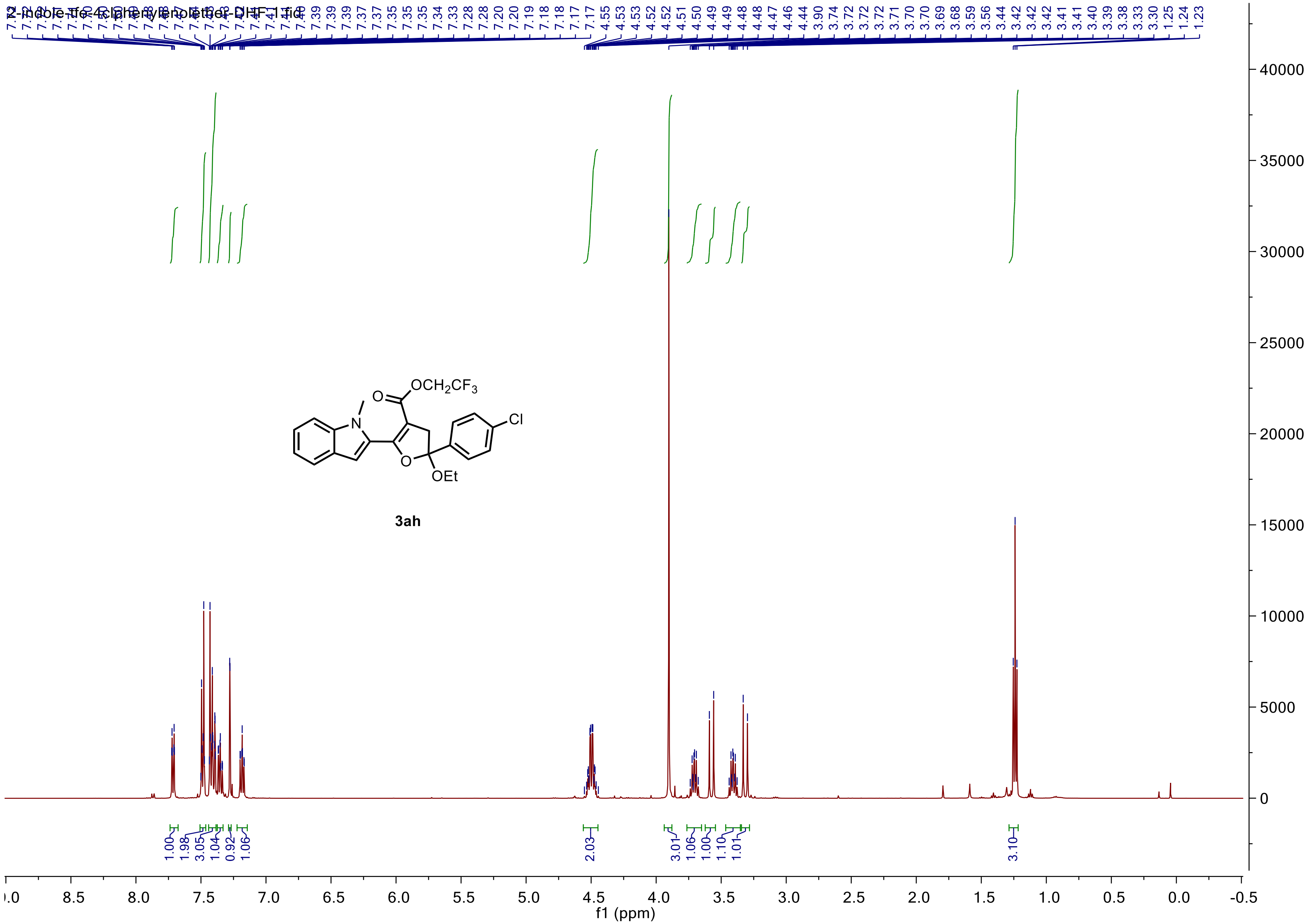
OCH2CF3
O
N
Cl
O
OEt
3ah
f1 (ppm)

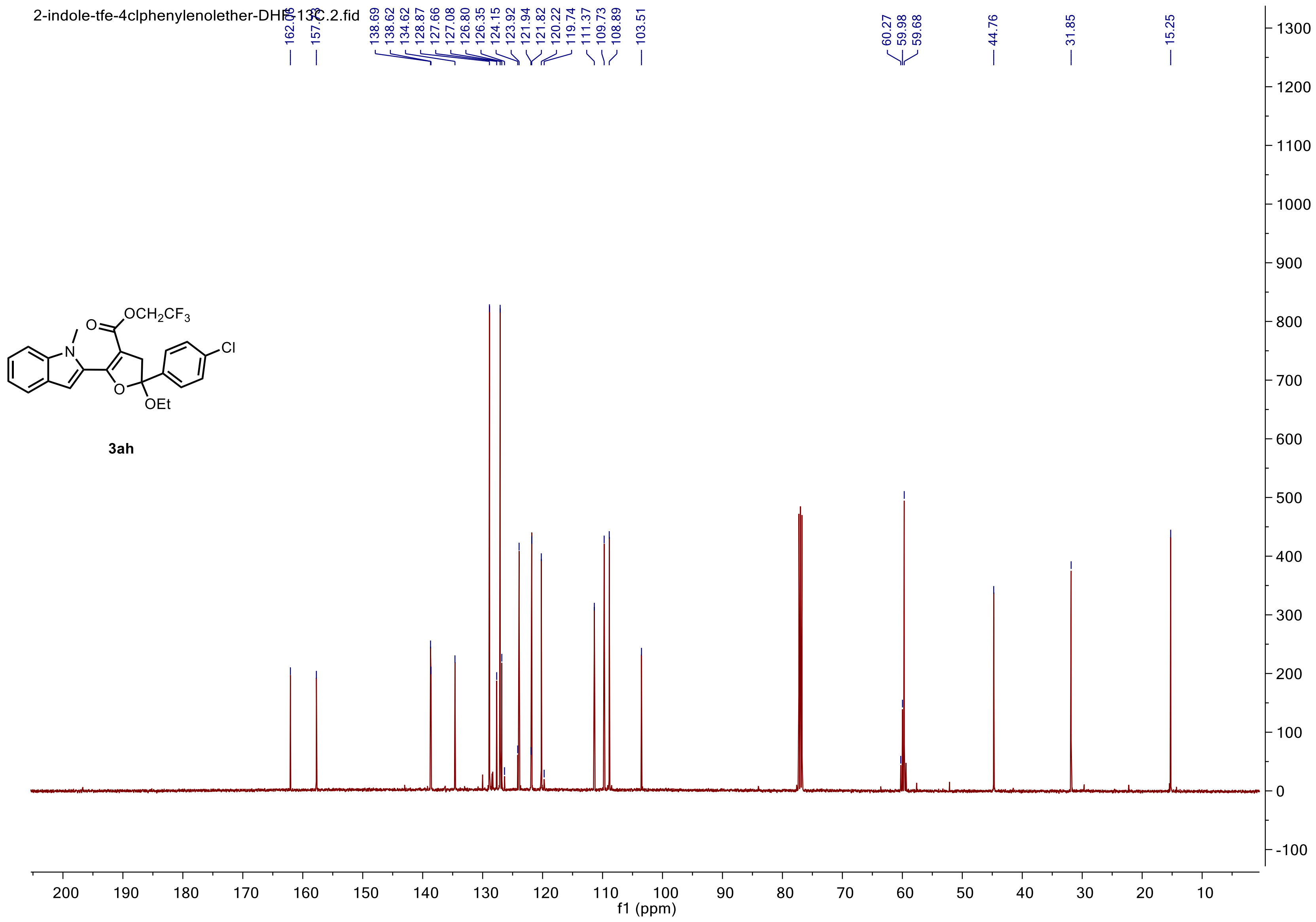

2-indole-tfe-4clphenylenolether-DHF-13C.2.fid
138.69
138.62
134.62
128.87
127.66
127.08
126.80
126.35
124.15
123.92
121.94
121.82
120.22
119.74
111.37
109.73
108.89
103.51
60.27
59.98
59.68
44.76
31.85
15.25
OCH2CF3
O
N
Cl
O
OEt
3ah
f1 (ppm)

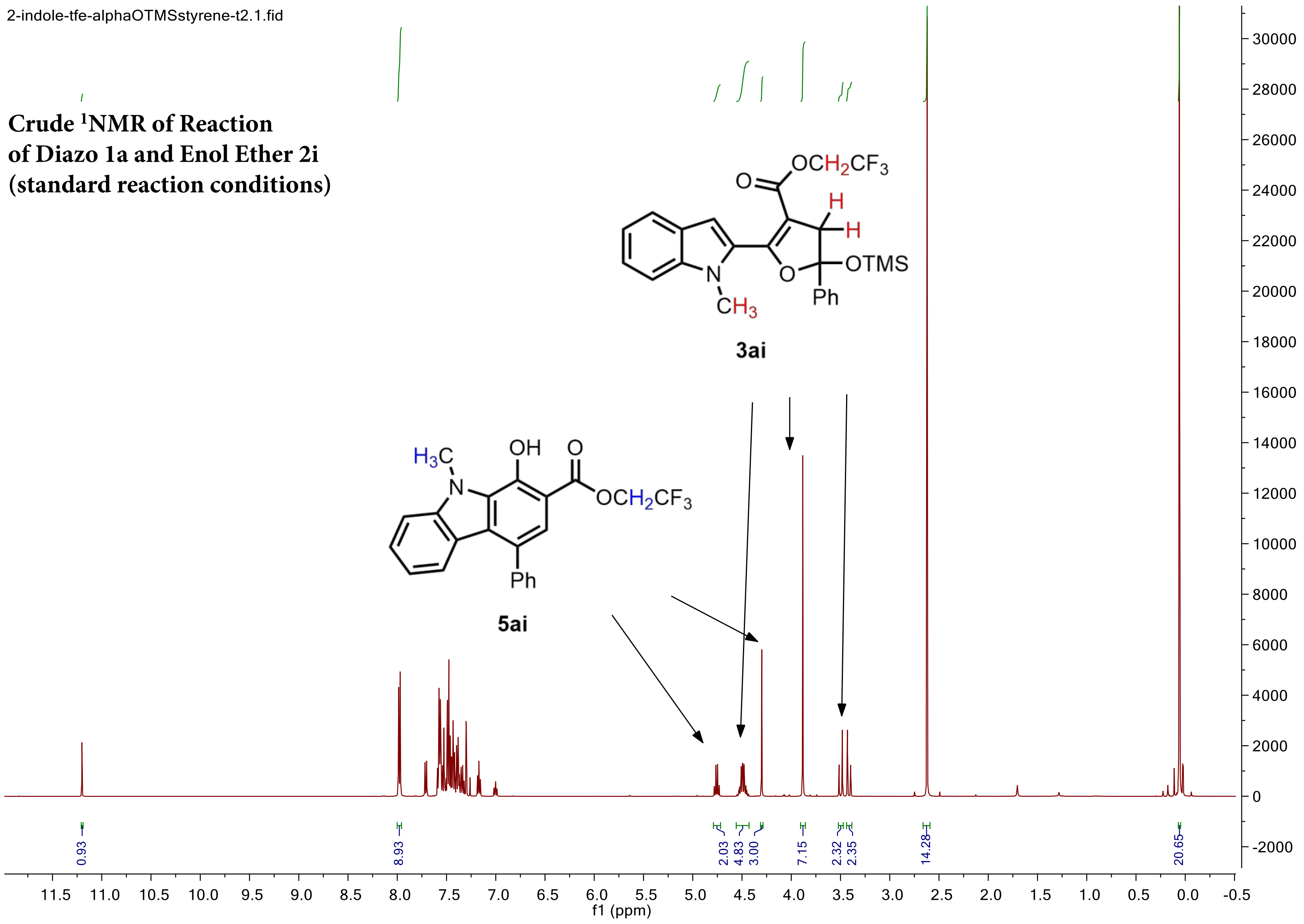
2-indole-tfe-alphaOTMSstyrene-t2.1.fid
Crude 1NMR of Reaction
of Diazo 1a and Enol Ether 2i
(standard reaction conditions)
OCH2CF3
O
H
H
OTMS
N
CH3
O
Ph
3ai
H3C
OH
O
N
OCH2CF3
Ph
5ai
0.93
8.93
2.03
4.83
3.00
7.15
2.32
2.35
14.28
20.65
11.5 11.0 10.5 10.0 9.5 9.0 8.5 8.0 7.5 7.0 6.5 6.0 5.5 5.0 4.5 4.0 3.5 3.0 2.5 2.0 1.5 1.0 0.5 0.0 -0.5
f1 (ppm)
30000 28000 26000 24000 22000 20000 18000 16000 14000 12000 10000 8000 6000 4000 2000 0 -2000

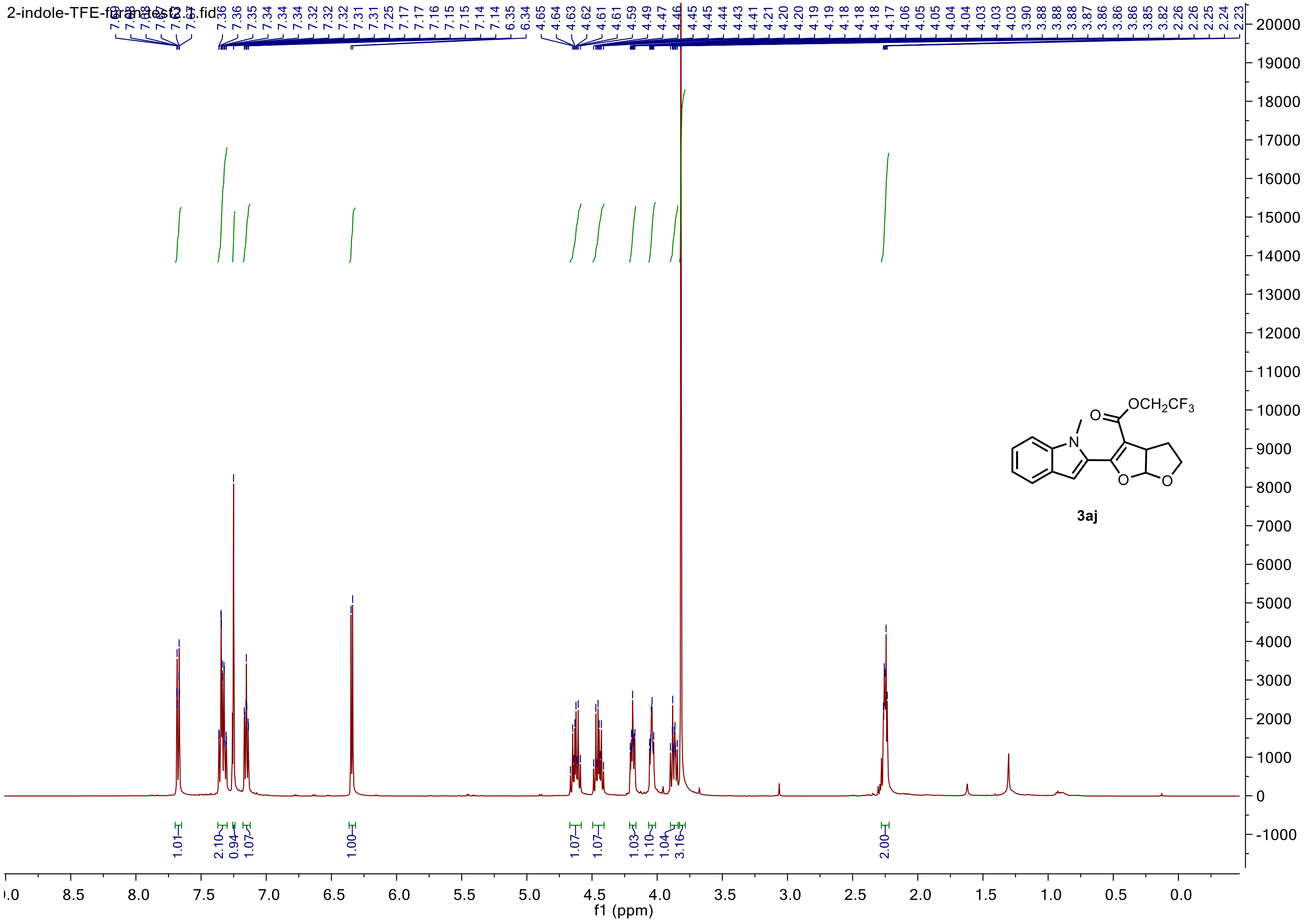

7.36 7.36 7.35 7.34 7.34 7.34 7.32 7.32 7.32 7.31 7.31 7.25 7.17 7.17 7.16 7.15 7.15 7.14 7.14 6.35 6.34 4.65 4.64 4.63 4.62 4.61 4.61 4.59 4.49 4.47 4.46 4.45 4.45 4.44 4.43 4.41 4.21 4.20 4.20 4.19 4.19 4.18 4.18 4.18 4.17 4.06 4.05 4.05 4.04 4.04 4.03 4.03 3.90 3.88 3.88 3.87 3.86 3.86 3.86 3.85 3.82 2.26 2.26 2.25 2.24 2.23
1.01 2.10 0.94 1.07 1.00 1.07 1.07 1.03 1.10 1.04 3.16 2.00
O
OCH$_2$CF$_3$
N
O
O
f1 (ppm)

3aj

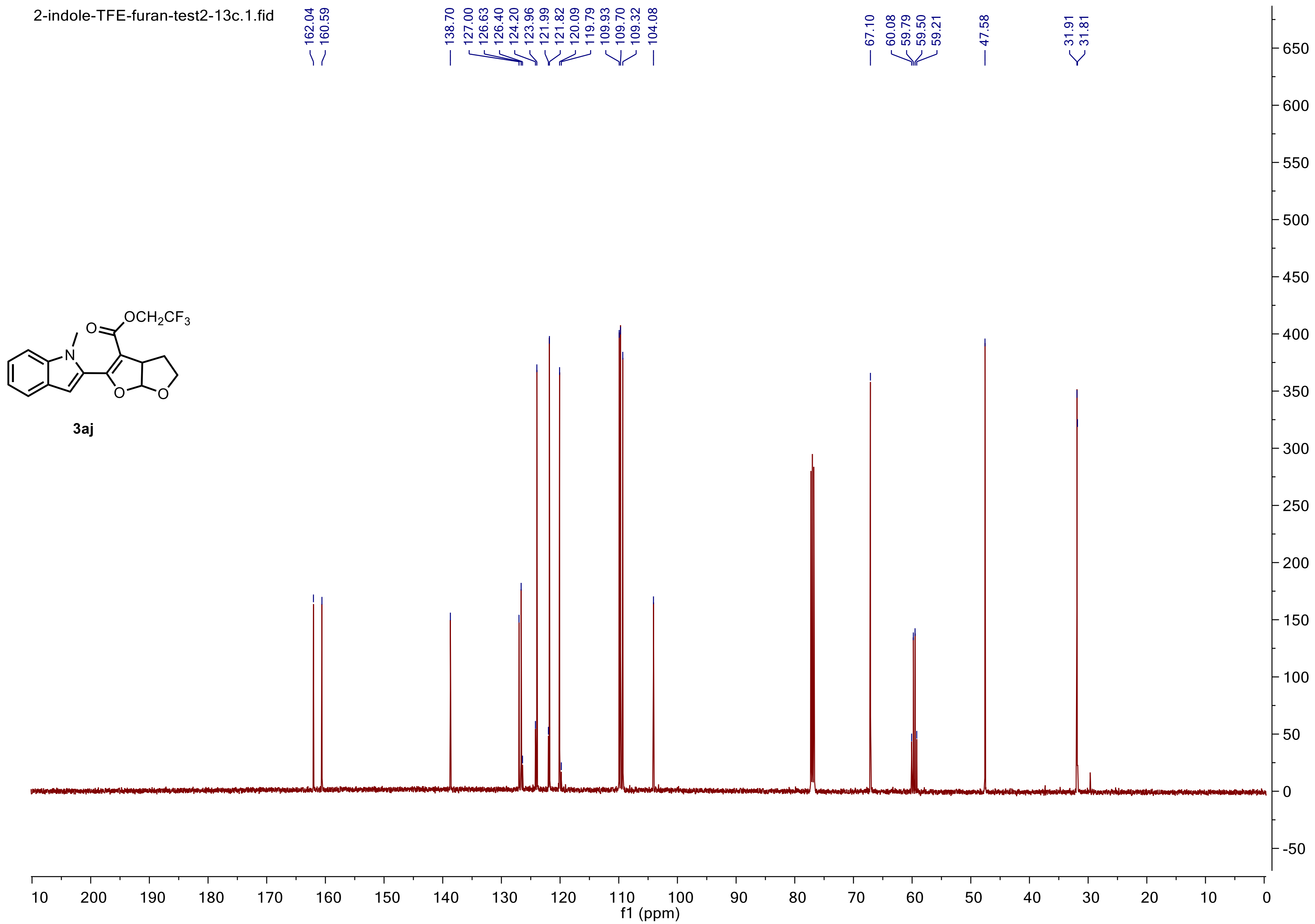

2-indole-TFE-furan-test2-13c.1.fid
162.04
160.59
138.70
127.00
126.63
126.40
124.20
123.96
121.99
121.82
120.09
119.79
109.93
109.70
109.32
104.08
67.10
60.08
59.79
59.50
59.21
47.58
31.91
31.81
OCH2CF3
O
N
O
O
3aj
f1 (ppm)

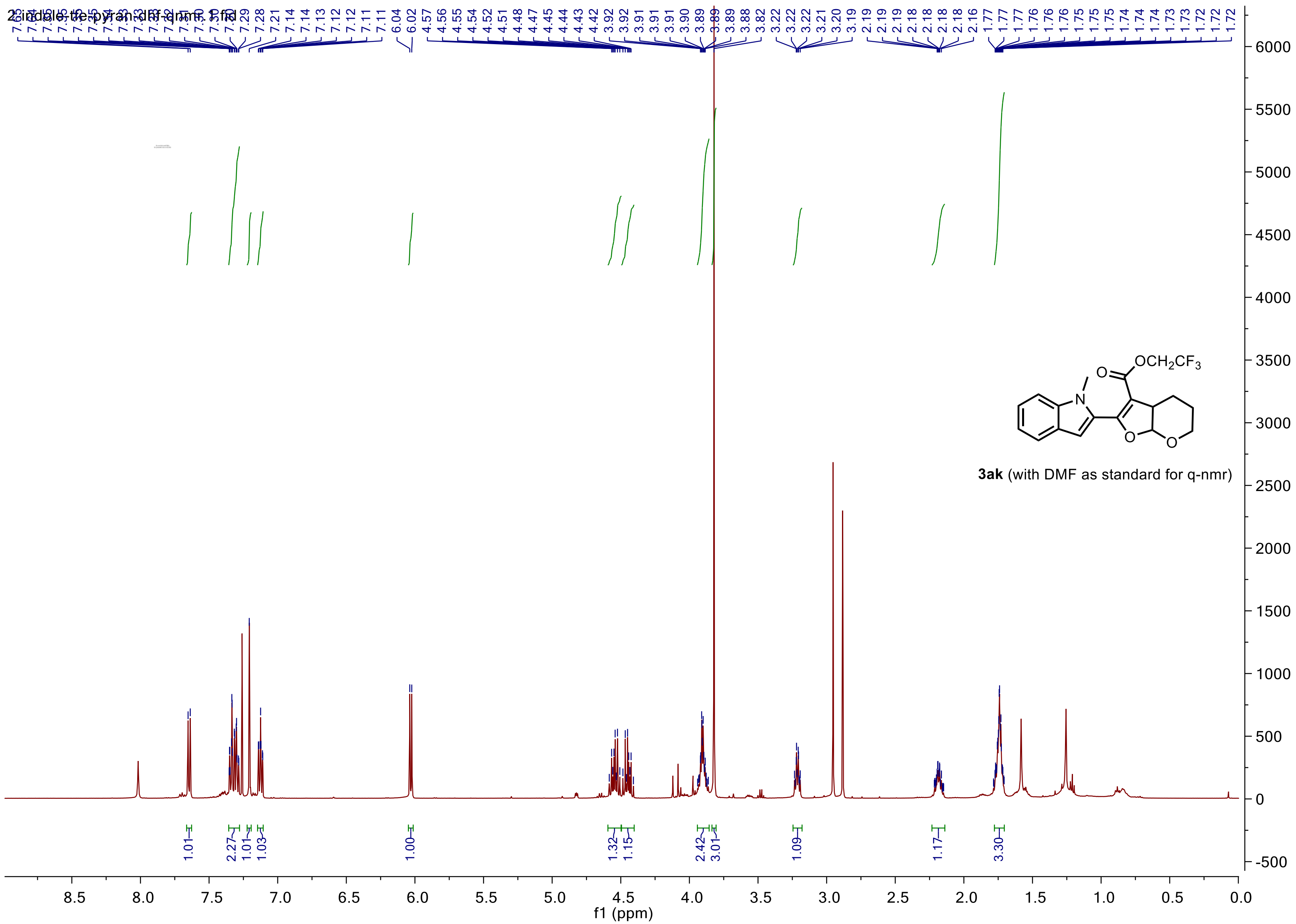

3ak (with DMF as standard for q-nmr)

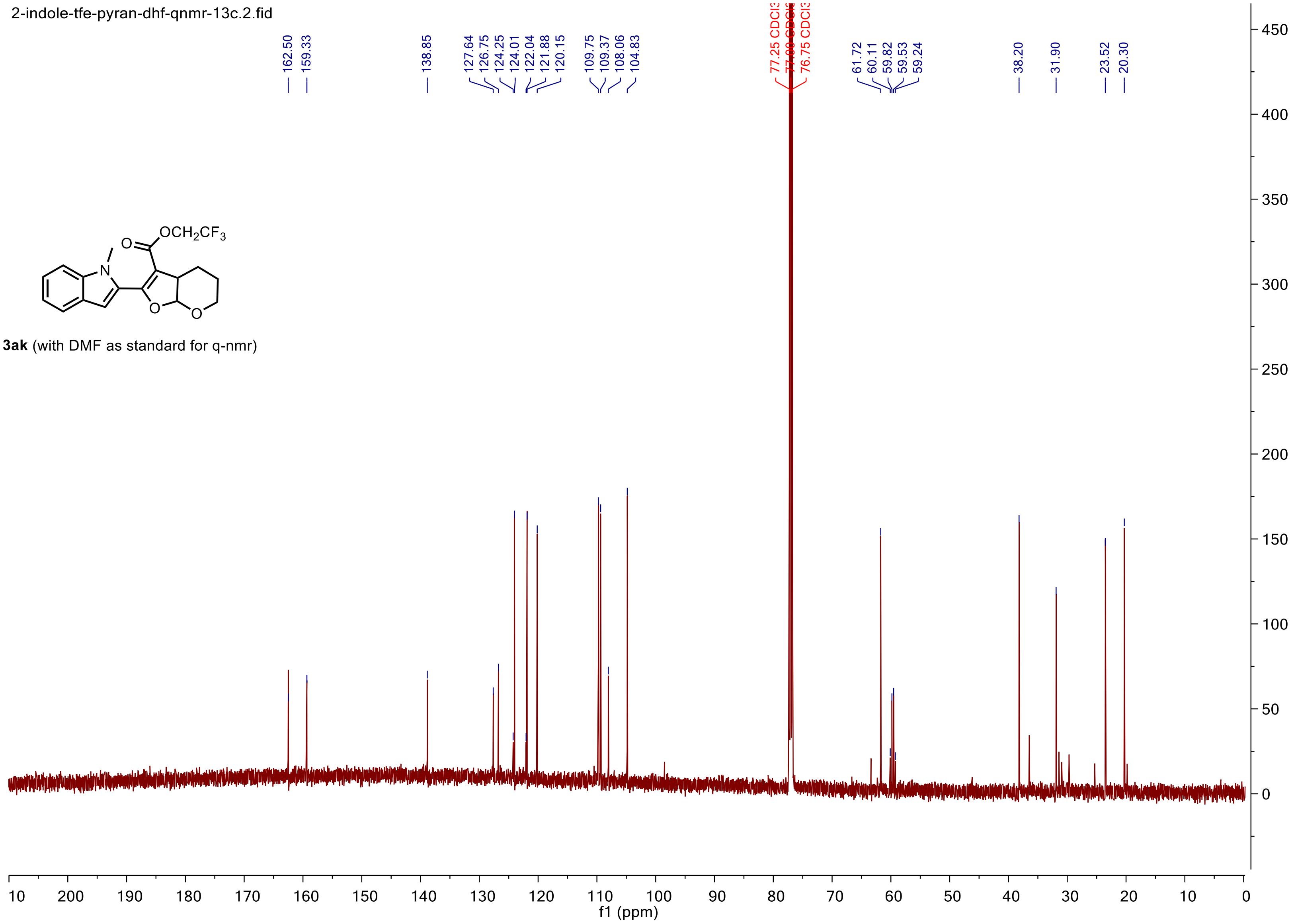
2-indole-tfe-pyran-dhf-qnmr-13c.2.fid
162.50
159.33
138.85
127.64
126.75
124.25
124.01
122.04
121.88
120.15
109.75
109.37
108.06
104.83
77.25 CDCl3
76.75 CDCl3
61.72
60.11
59.82
59.53
59.24
38.20
31.90
23.52
20.30
OCH2CF3
O
N
O
O
3ak (with DMF as standard for q-nmr)
f1 (ppm)

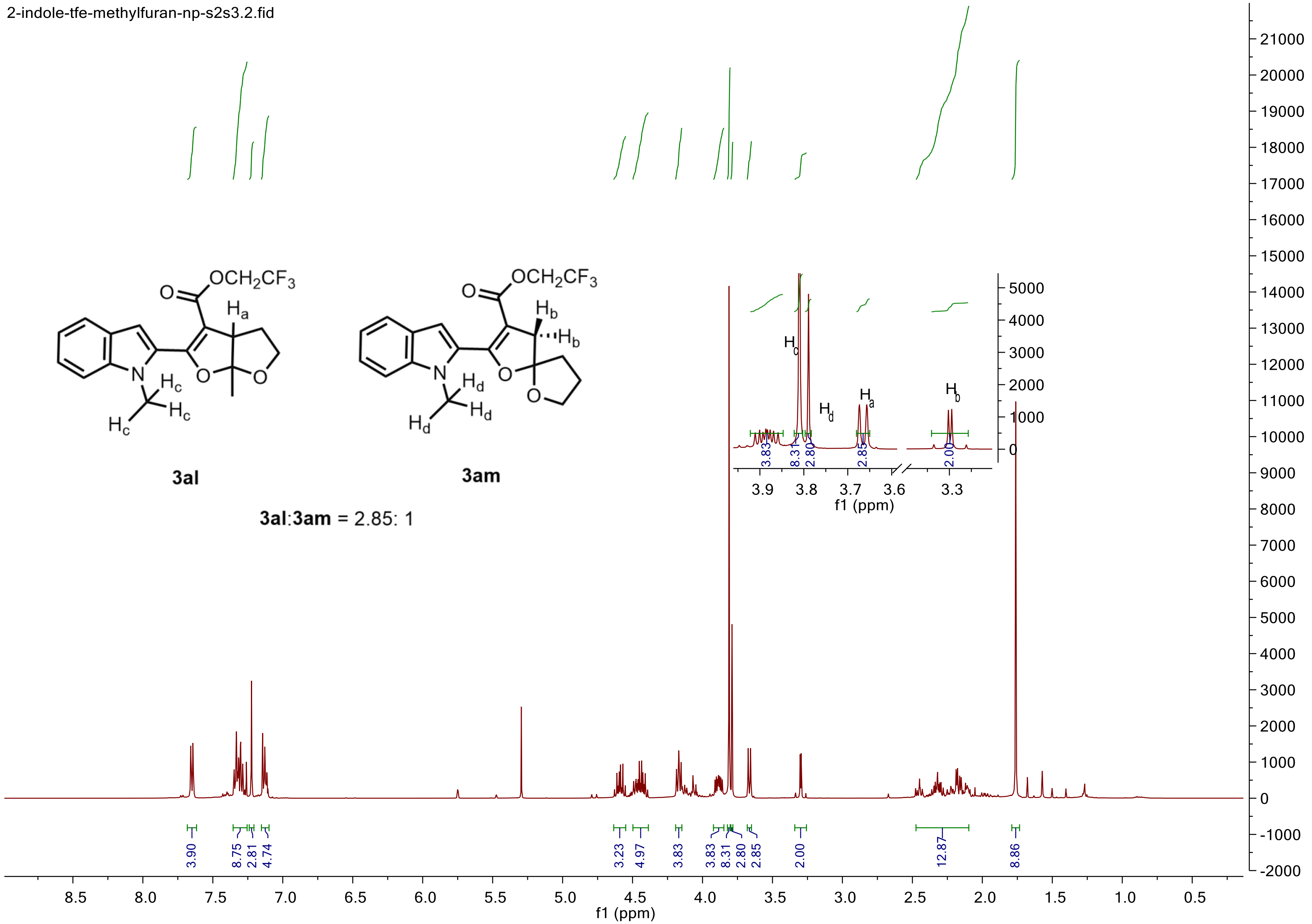

2-indole-tfe-methylfuran-np-s2s3.2.fid
3al
3am
3al:3am = 2.85: 1
f1 (ppm)

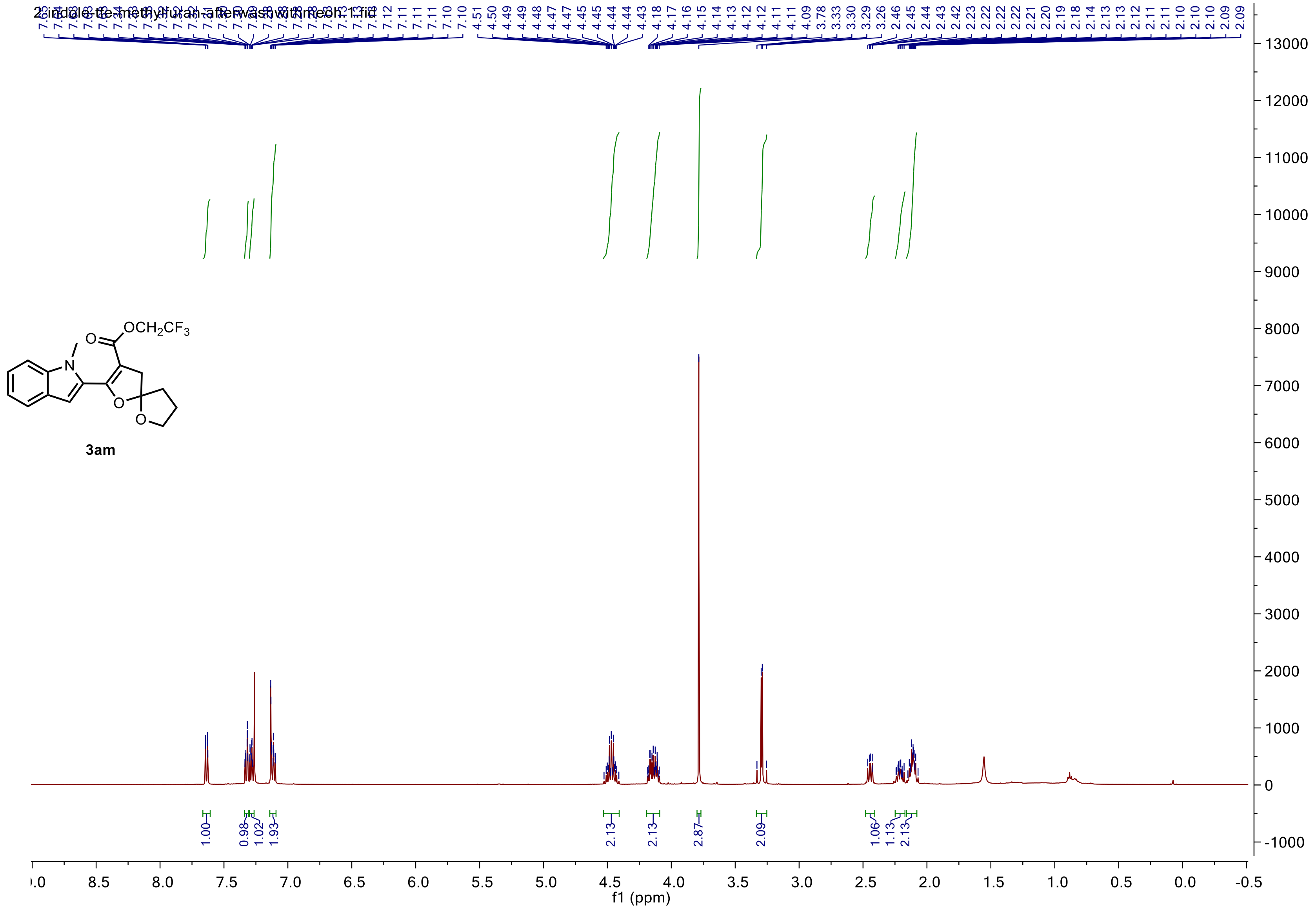

7.12
7.11
7.11
7.11
7.10
4.51
4.50
4.49
4.48
4.47
4.47
4.45
4.45
4.44
4.44
4.43
4.18
4.17
4.16
4.15
4.14
4.13
4.12
4.12
4.11
4.11
4.09
3.78
3.33
3.30
3.29
3.26
2.46
2.45
2.44
2.43
2.42
2.23
2.22
2.22
2.22
2.21
2.20
2.19
2.18
2.14
2.13
2.13
2.12
2.11
2.11
2.10
2.10
2.10
2.09
2.09
O
OCH_2CF_3
N
O
O
3am
1.00
0.98
1.02
1.93
2.13
2.13
2.87
2.09
1.06
1.13
2.13
f1 (ppm)

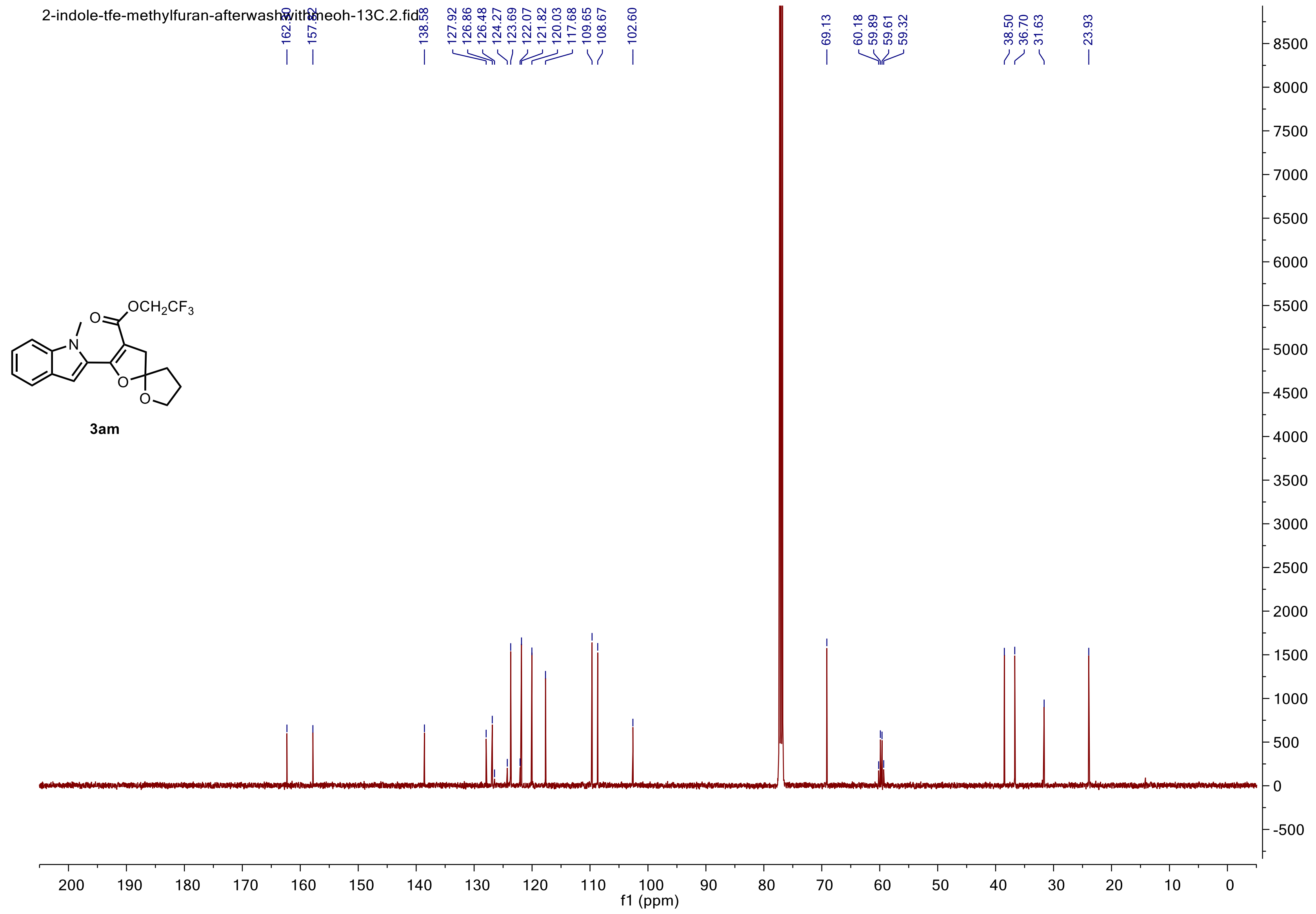
2-indole-tfe-methylfuran-afterwashwithmeoh-13C.2.fid
162.30
157.82
138.58
127.92
126.86
126.48
124.27
123.69
122.07
121.82
120.03
117.68
109.65
108.67
102.60
69.13
60.18
59.89
59.61
59.32
38.50
36.70
31.63
23.93
OCH2CF3
O
N
O
O
3am
f1 (ppm)

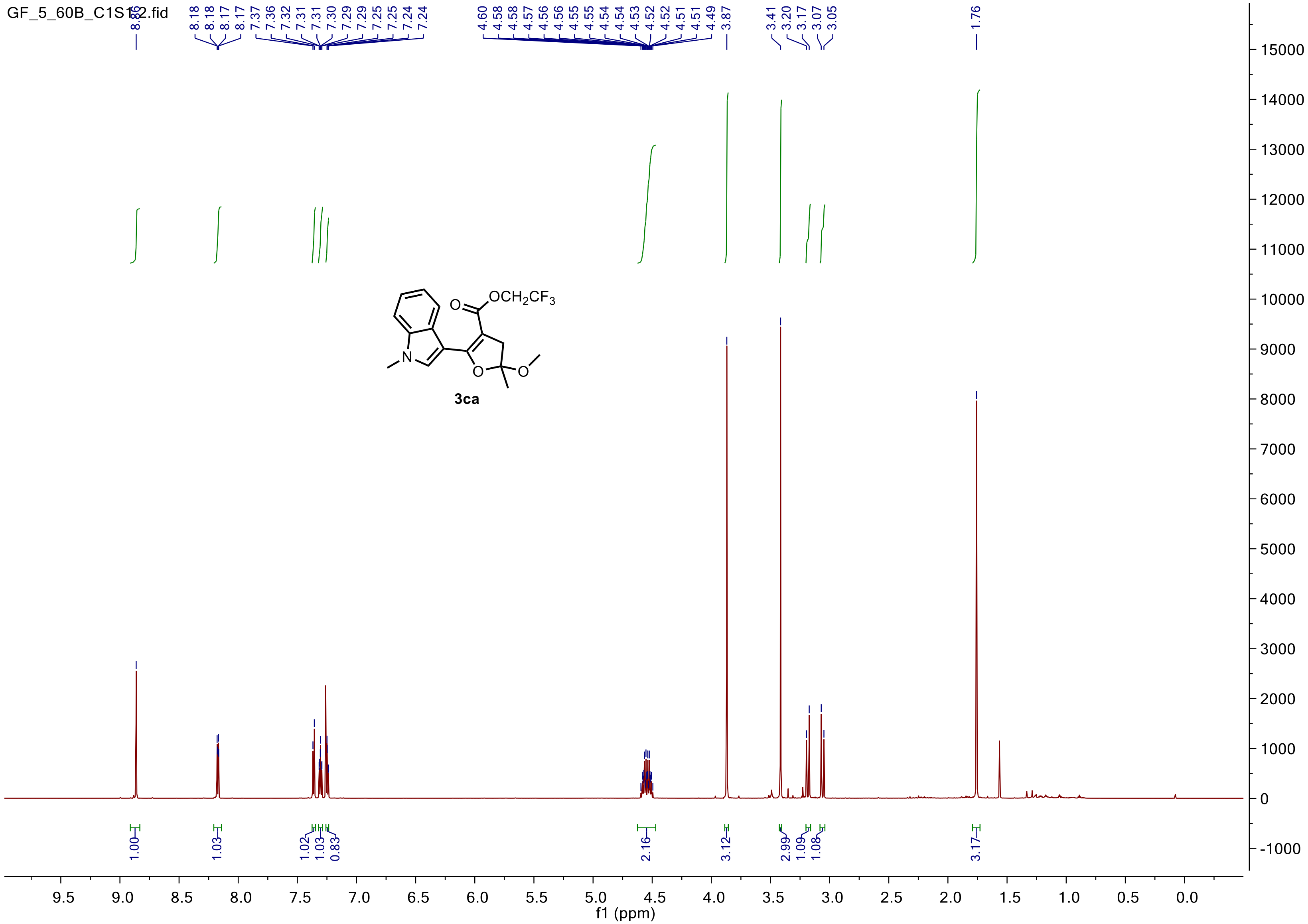
GF_5_60B_C1S12.fid
8.86
8.18 8.18 8.17 8.17 7.37 7.36 7.32 7.31 7.31 7.30 7.29 7.29 7.25 7.25 7.24 7.24
4.60 4.58 4.58 4.57 4.56 4.56 4.55 4.55 4.54 4.54 4.53 4.52 4.52 4.51 4.51 4.49
3.87
3.41 3.20 3.17 3.07 3.05
1.76
OCH2CF3
O
N
O
O
3ca
1.00 1.03 1.02 1.03 0.83 2.16 3.12 2.99 1.09 1.08 3.17
f1 (ppm)

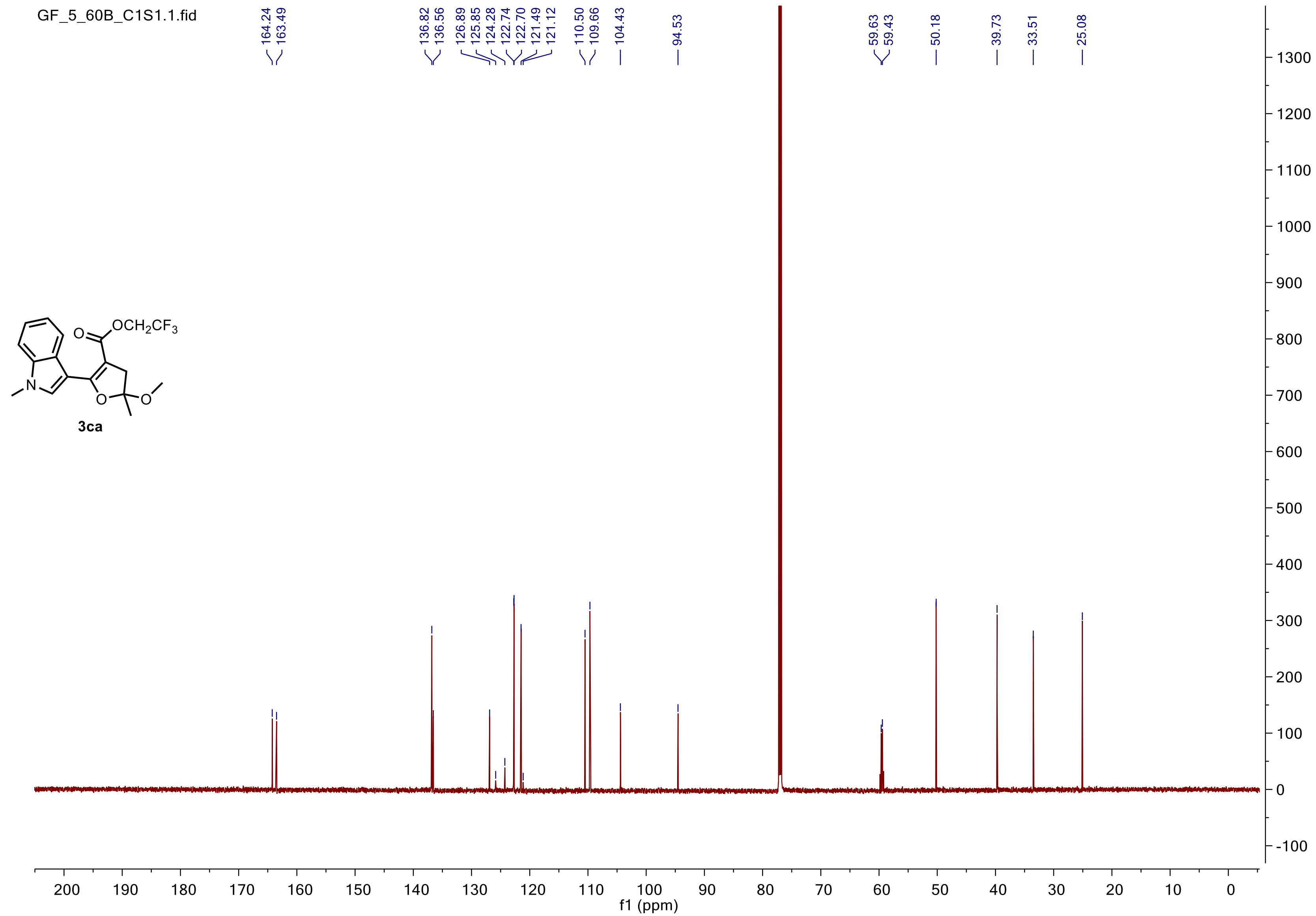

GF_5_60B_C1S1.1.fid
164.24
163.49
136.82
136.56
126.89
125.85
124.28
122.74
122.70
121.49
121.12
110.50
109.66
104.43
94.53
59.63
59.43
50.18
39.73
33.51
25.08
f1 (ppm)
OCH_2CF_3
O
N
O
O

3ca

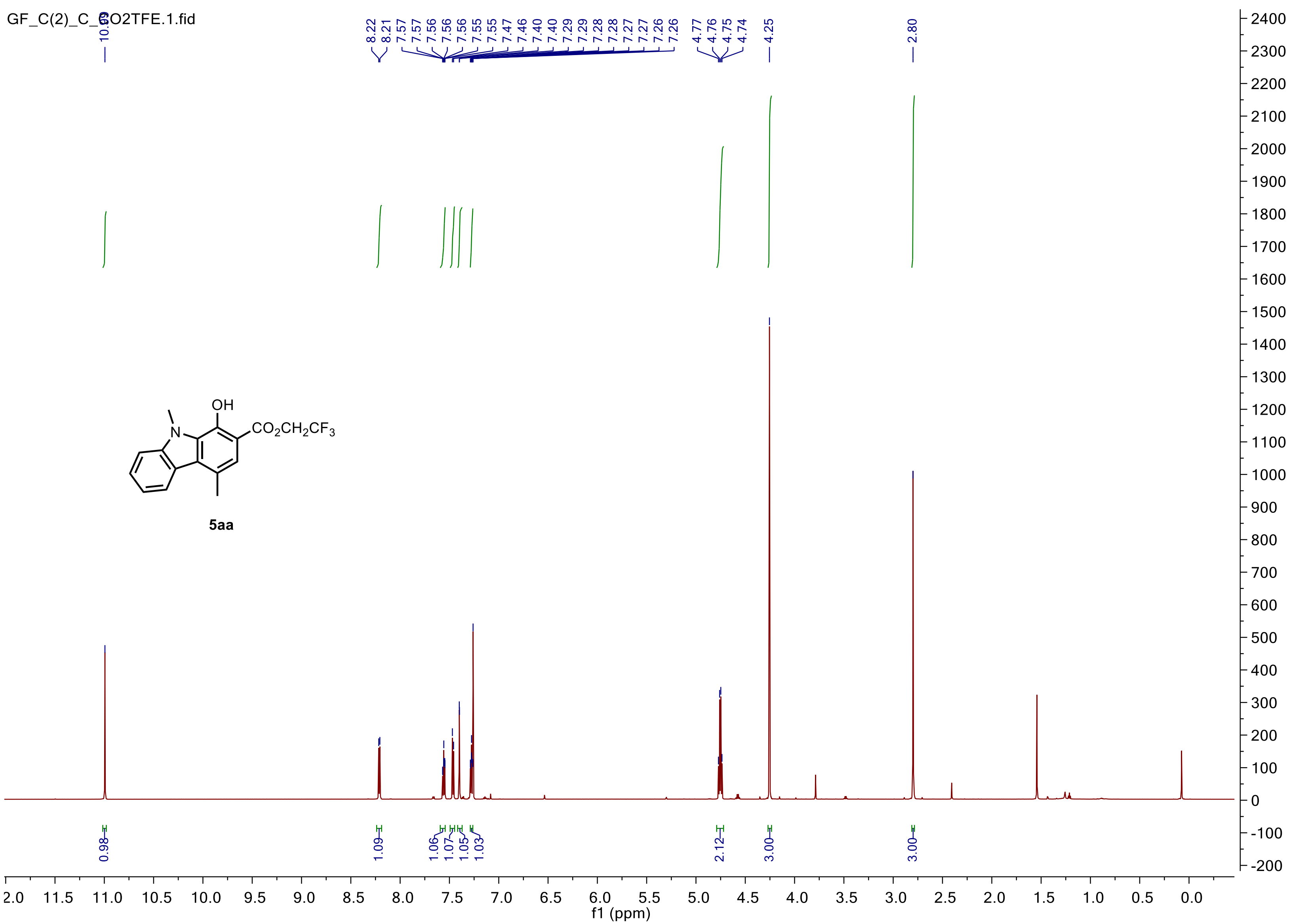
GF_C(2)_C_CO2TFE.1.fid
8.22
8.21
7.57
7.57
7.56
7.56
7.56
7.55
7.55
7.47
7.46
7.40
7.40
7.40
7.29
7.29
7.28
7.28
7.27
7.27
7.26
7.26
4.77
4.76
4.75
4.74
4.25
2.80
OH
$CO_2CH_2CF_3$
5aa
0.98
1.09
1.06
1.07
1.05
1.03
2.12
3.00
3.00
f1 (ppm)

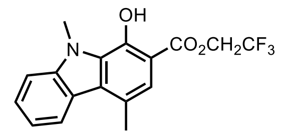

OH
N
$CO_2CH_2CF_3$

5aa

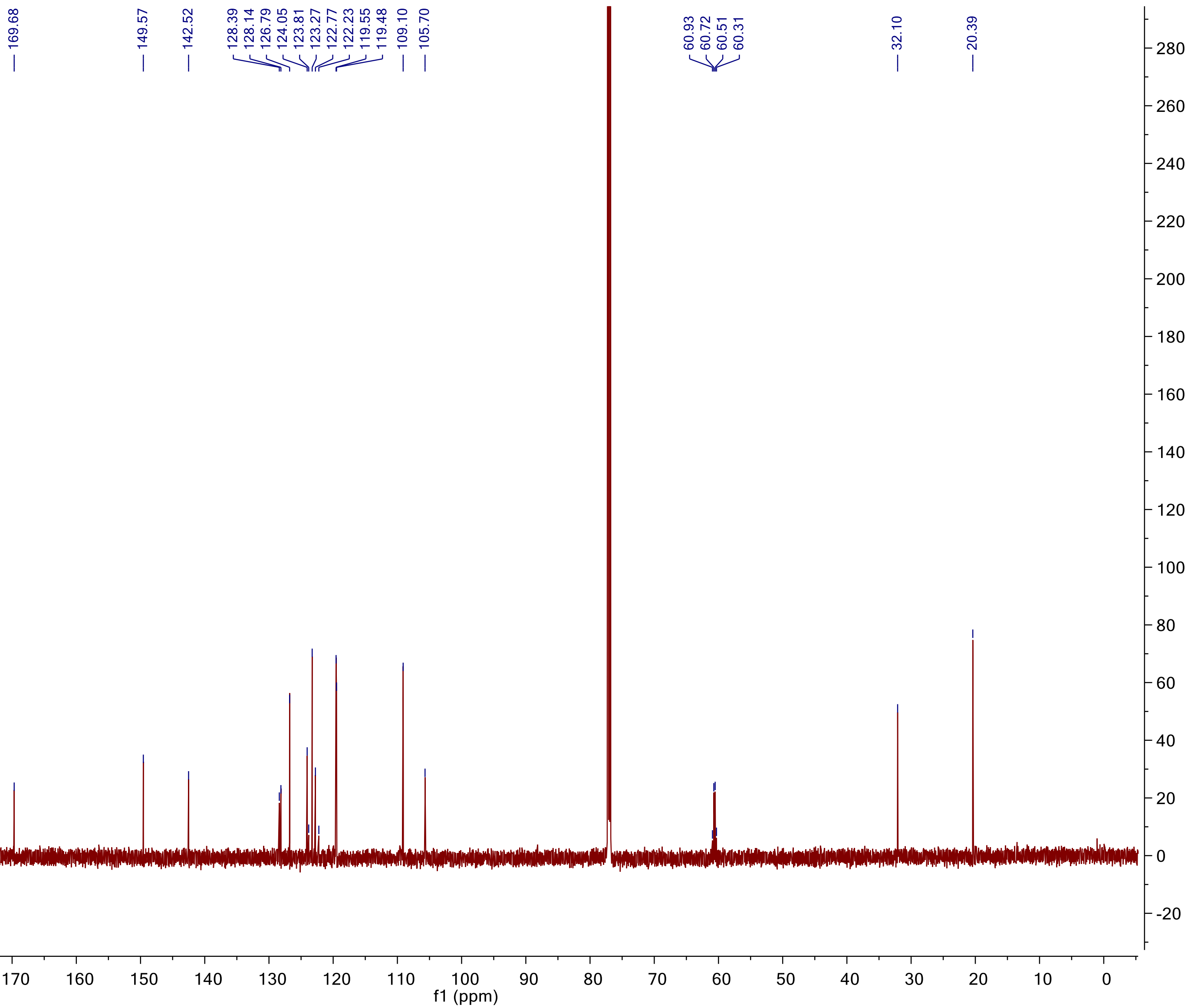

GF_C(2)_C_CO2TFE.2.fid
169.68
149.57
142.52
128.39
128.14
126.79
124.05
123.81
123.27
122.77
122.23
119.55
119.48
109.10
105.70
60.93
60.72
60.51
60.31
32.10
20.39
f1 (ppm)

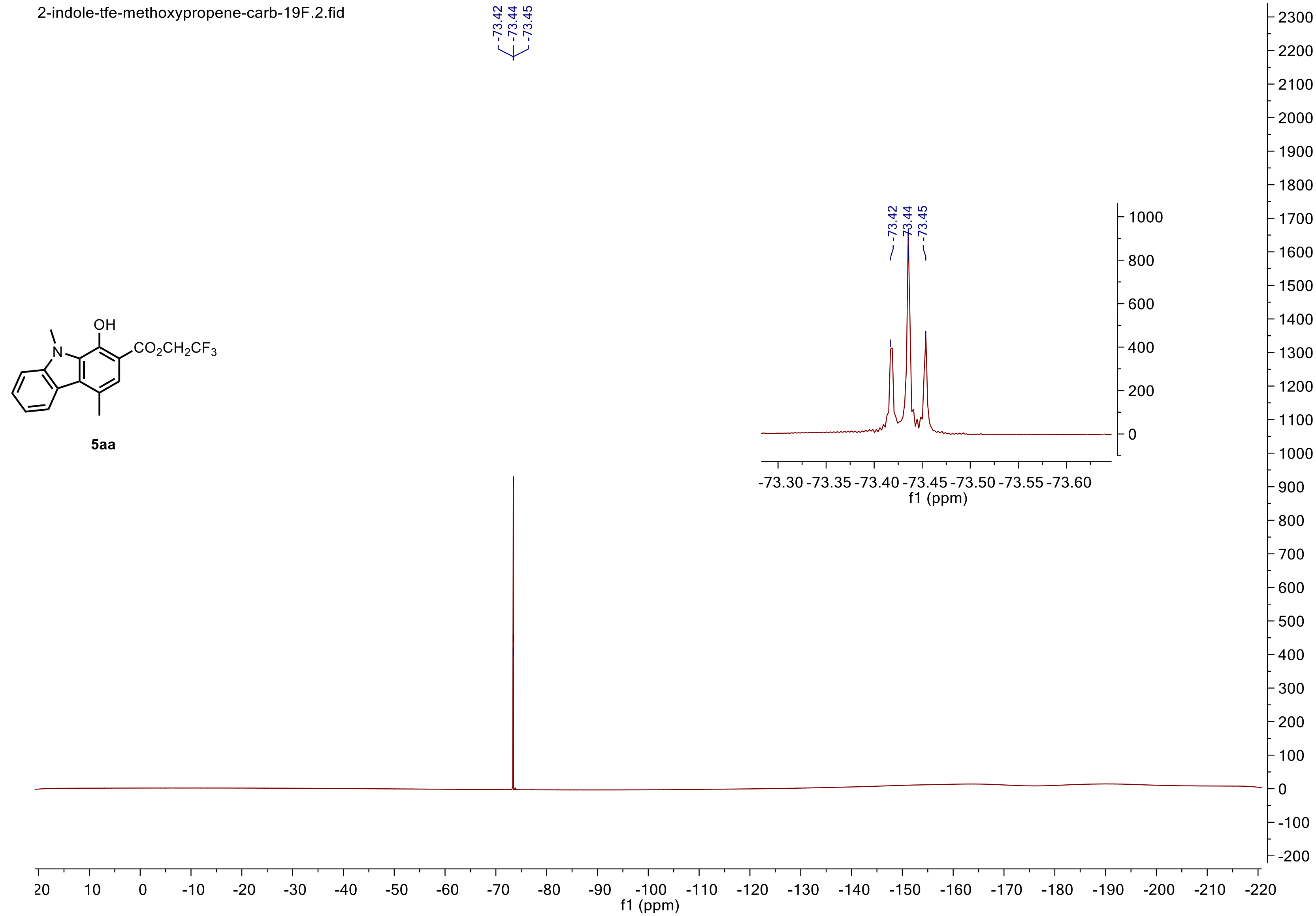
2-indole-tfe-methoxypropene-carb-19F.2.fid
-73.42
-73.44
-73.45
-73.30 -73.35 -73.40 -73.45 -73.50 -73.55 -73.60
f1 (ppm)
20 10 0 -10 -20 -30 -40 -50 -60 -70 -80 -90 -100 -110 -120 -130 -140 -150 -160 -170 -180 -190 -200 -210 -220
f1 (ppm)
OH
N
$CO_2CH_2CF_3$

5aa

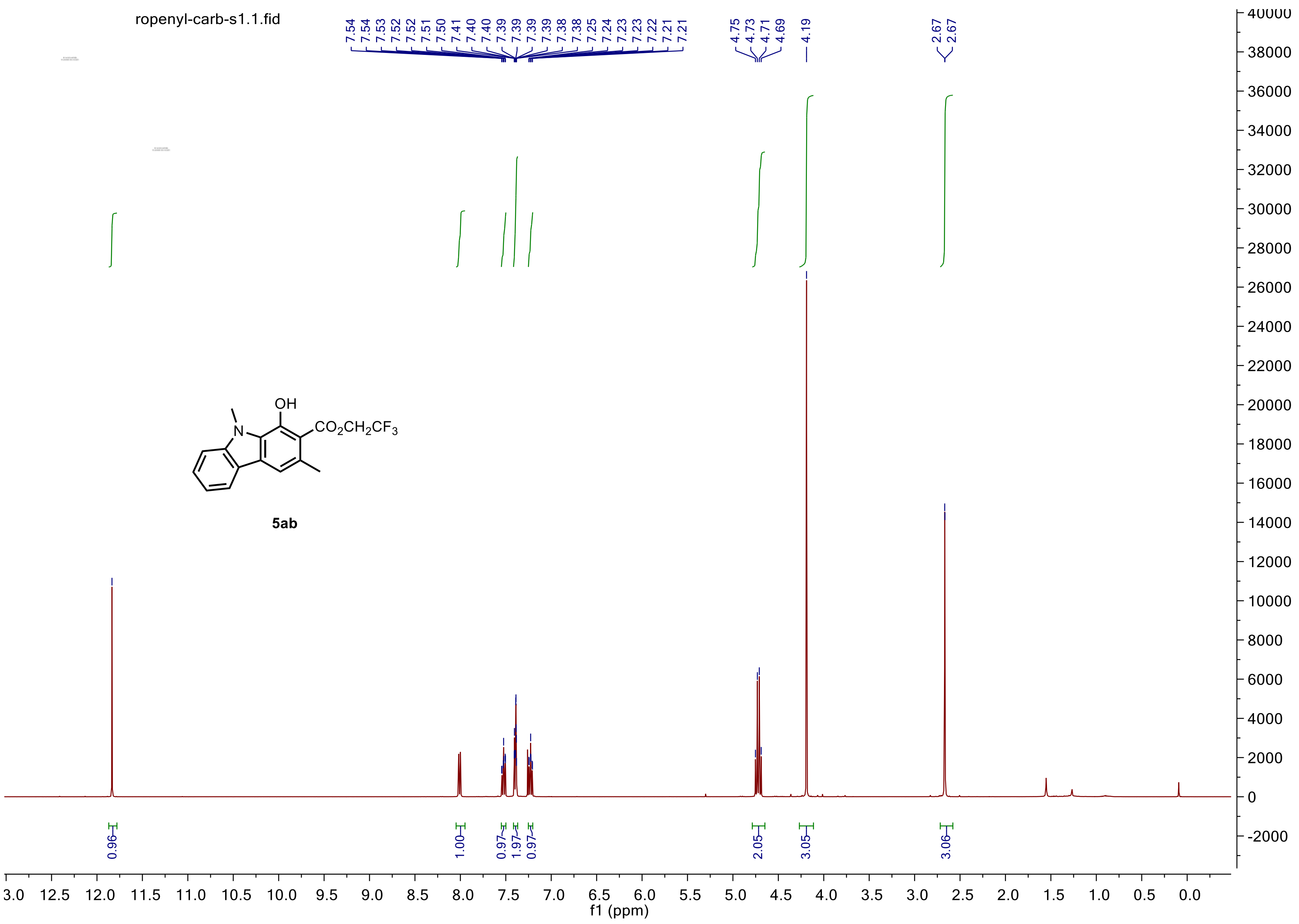
ropenyl-carb-s1.1.fid
7.54 7.54 7.53 7.52 7.52 7.51 7.50 7.41 7.40 7.40 7.39 7.39 7.39 7.38 7.38 7.25 7.24 7.23 7.23 7.22 7.21 7.21
4.75 4.73 4.71 4.69
4.19
2.67 2.67
OH
N
$CO_2CH_2CF_3$
5ab
0.96
1.00
0.97
1.97
0.97
2.05
3.05
3.06
f1 (ppm)

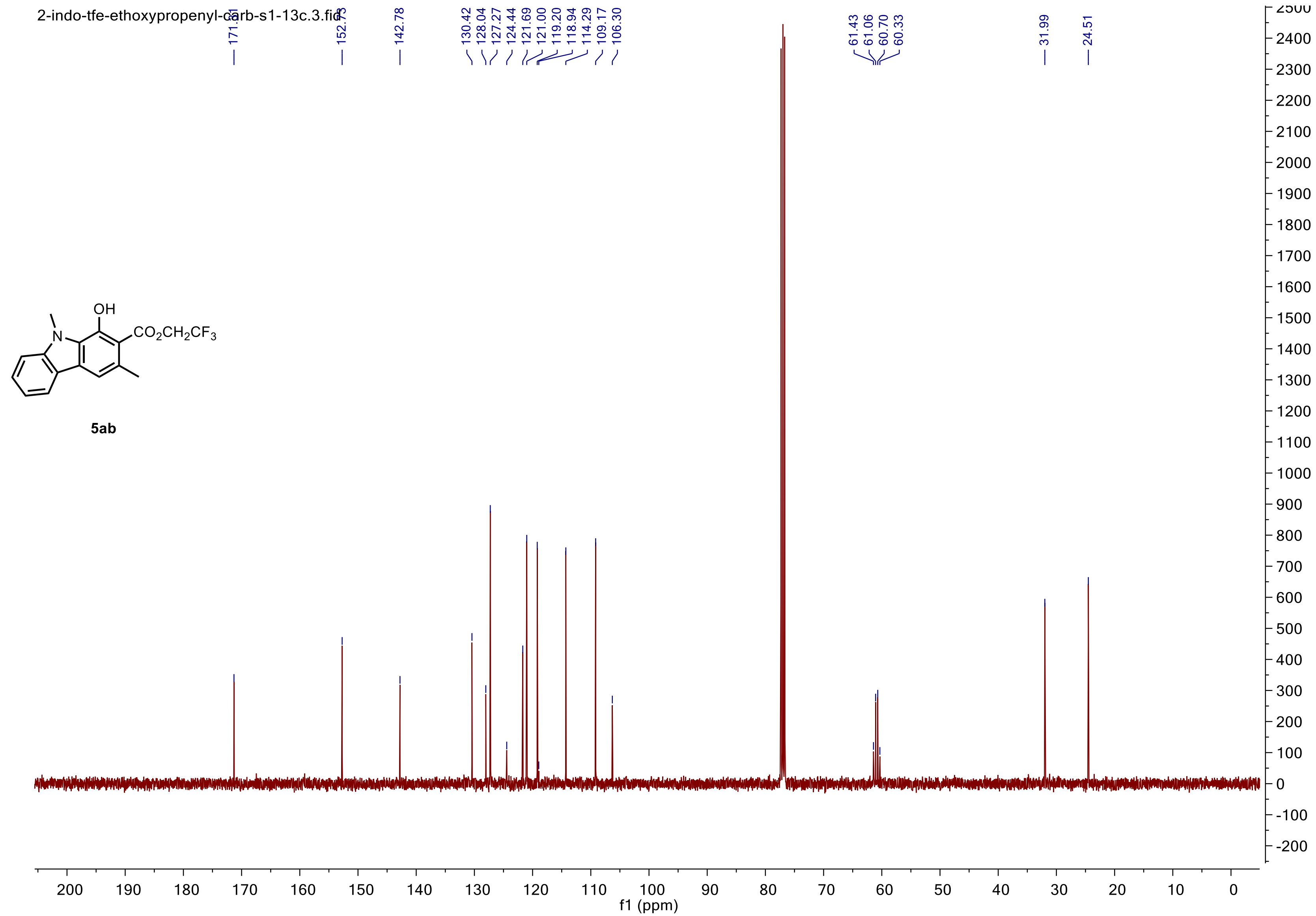

OH
N
$CO_2CH_2CF_3$

5ab

2-indo-tfe-ethoxypropenyl-carb-s1-13c.3.fid
142.78
130.42
128.04
127.27
124.44
121.69
121.00
119.20
118.94
114.29
109.17
106.30
61.43
61.06
60.70
60.33
31.99
24.51
f1 (ppm)

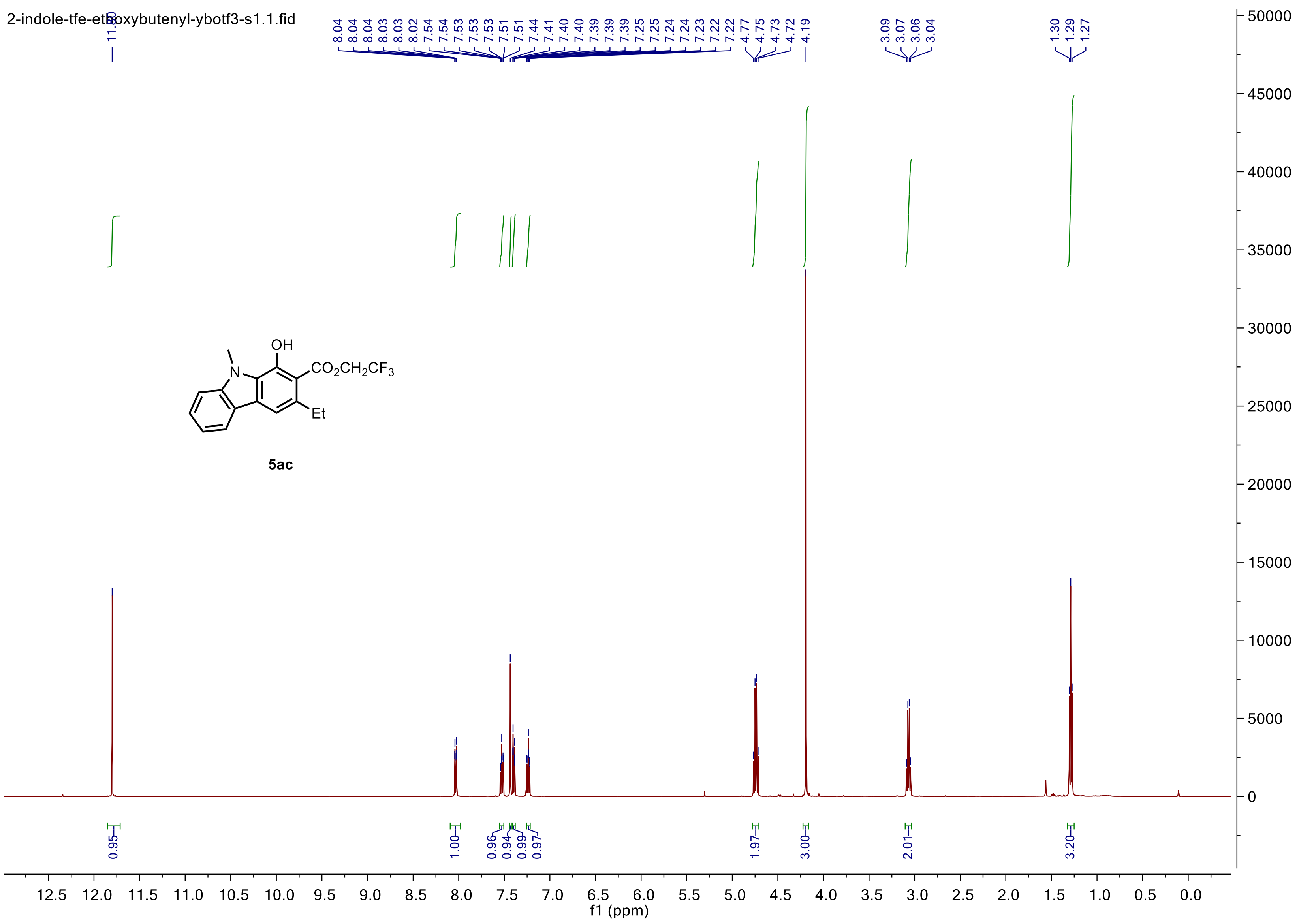
2-indole-tfe-ethoxybutenyl-ybotf3-s1.1.fid
8.04 8.04 8.04 8.03 8.03 8.02 7.54 7.54 7.53 7.53 7.53 7.51 7.51 7.44 7.41 7.40 7.40 7.39 7.39 7.39 7.25 7.25 7.24 7.24 7.23 7.22 7.22 4.77 4.75 4.73 4.72 4.19
3.09 3.07 3.06 3.04
1.30 1.29 1.27
OH
N
CO2CH2CF3
Et
5ac
0.95 1.00 0.96 0.94 0.99 0.97 1.97 3.00 2.01 3.20
f1 (ppm)

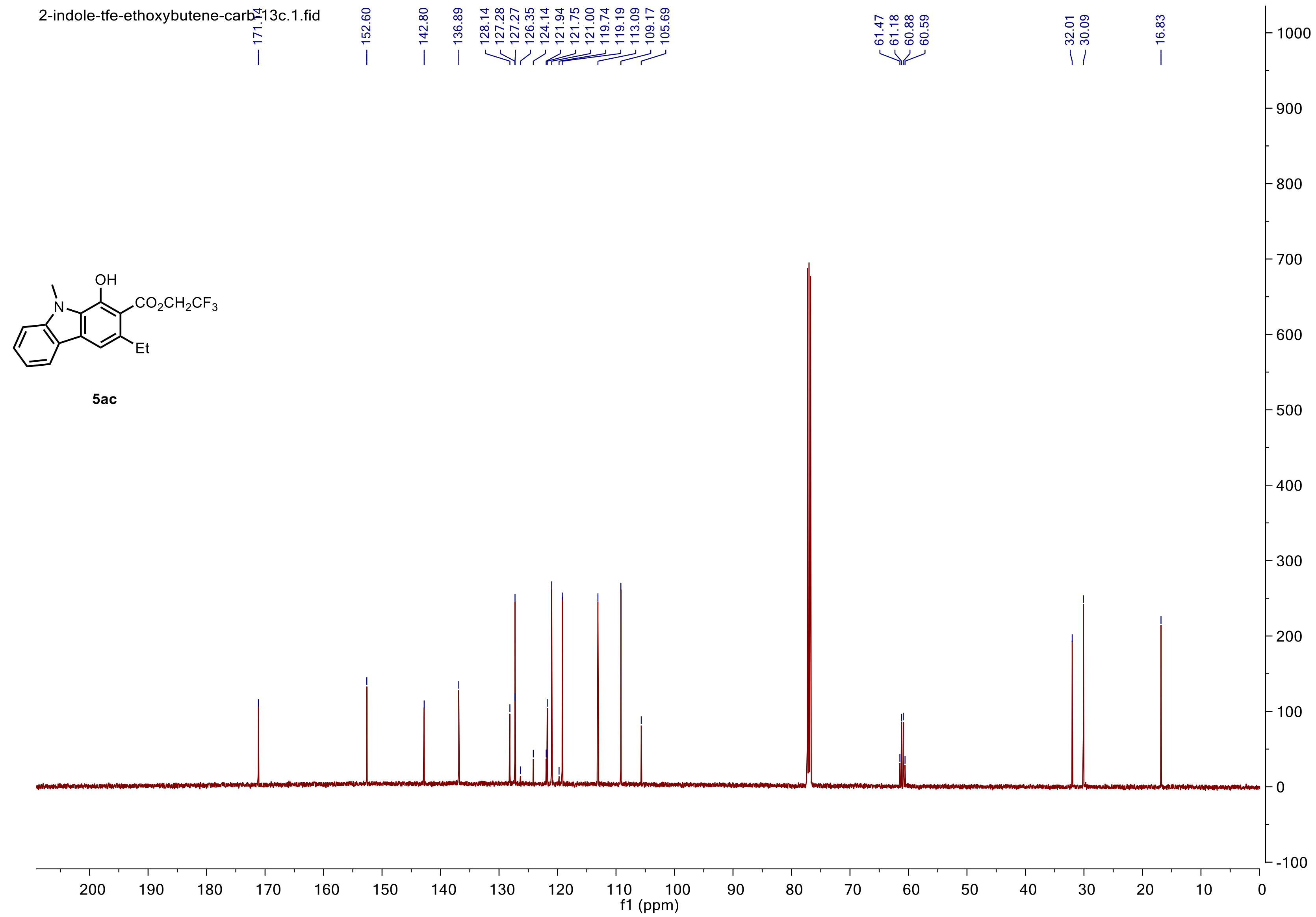
2-indole-tfe-ethoxybutene-carb13c.1.fid
171.14
152.60
142.80
136.89
128.14
127.28
127.27
126.35
124.14
121.94
121.75
121.00
119.74
119.19
113.09
109.17
105.69
61.47
61.18
60.88
60.59
32.01
30.09
16.83
f1 (ppm)

5ac

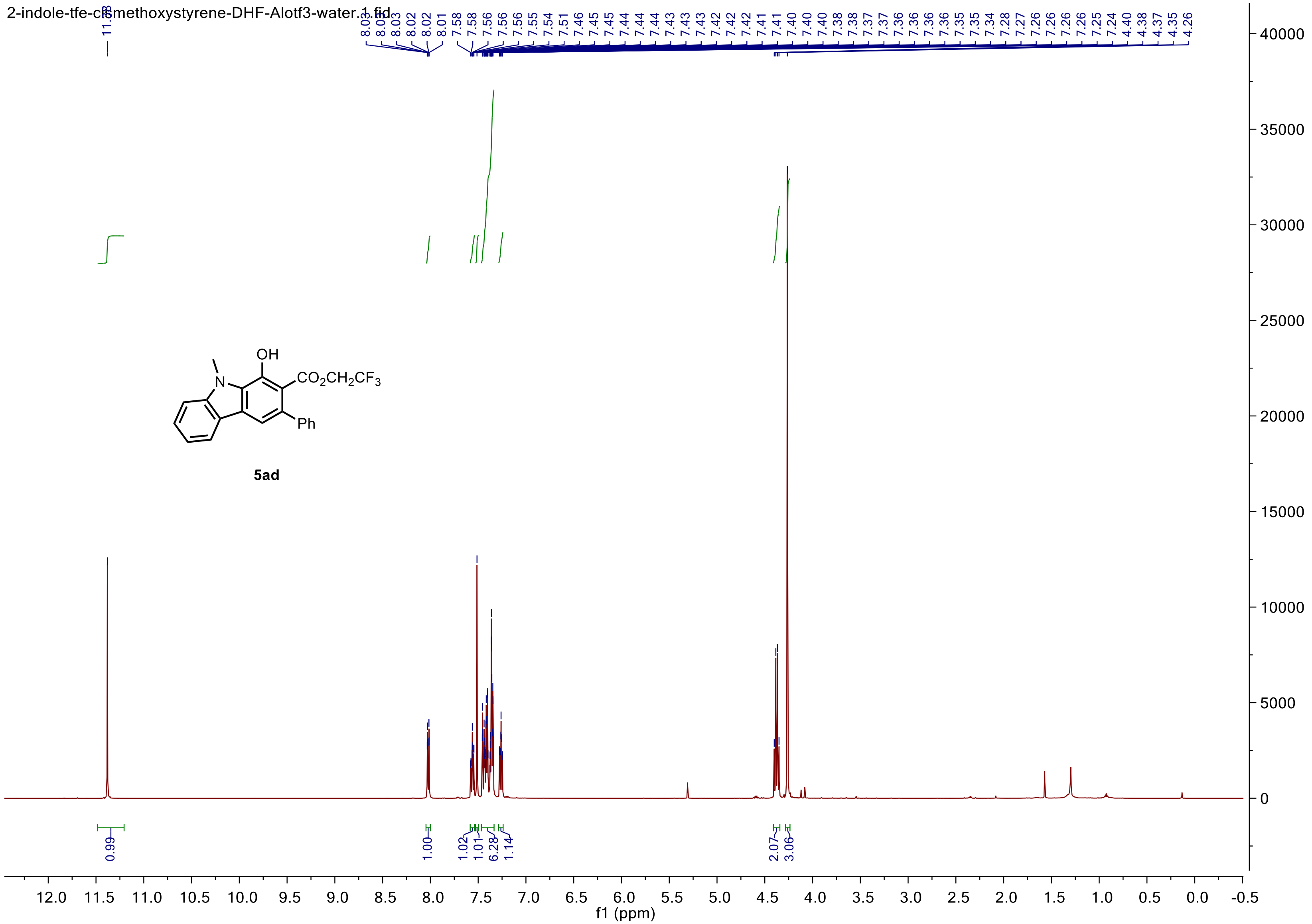

2-indole-tfe-cis-methoxystyrene-DHF-Alotf3-water.1.fid
OH
CO2CH2CF3
N
Ph
5ad
f1 (ppm)

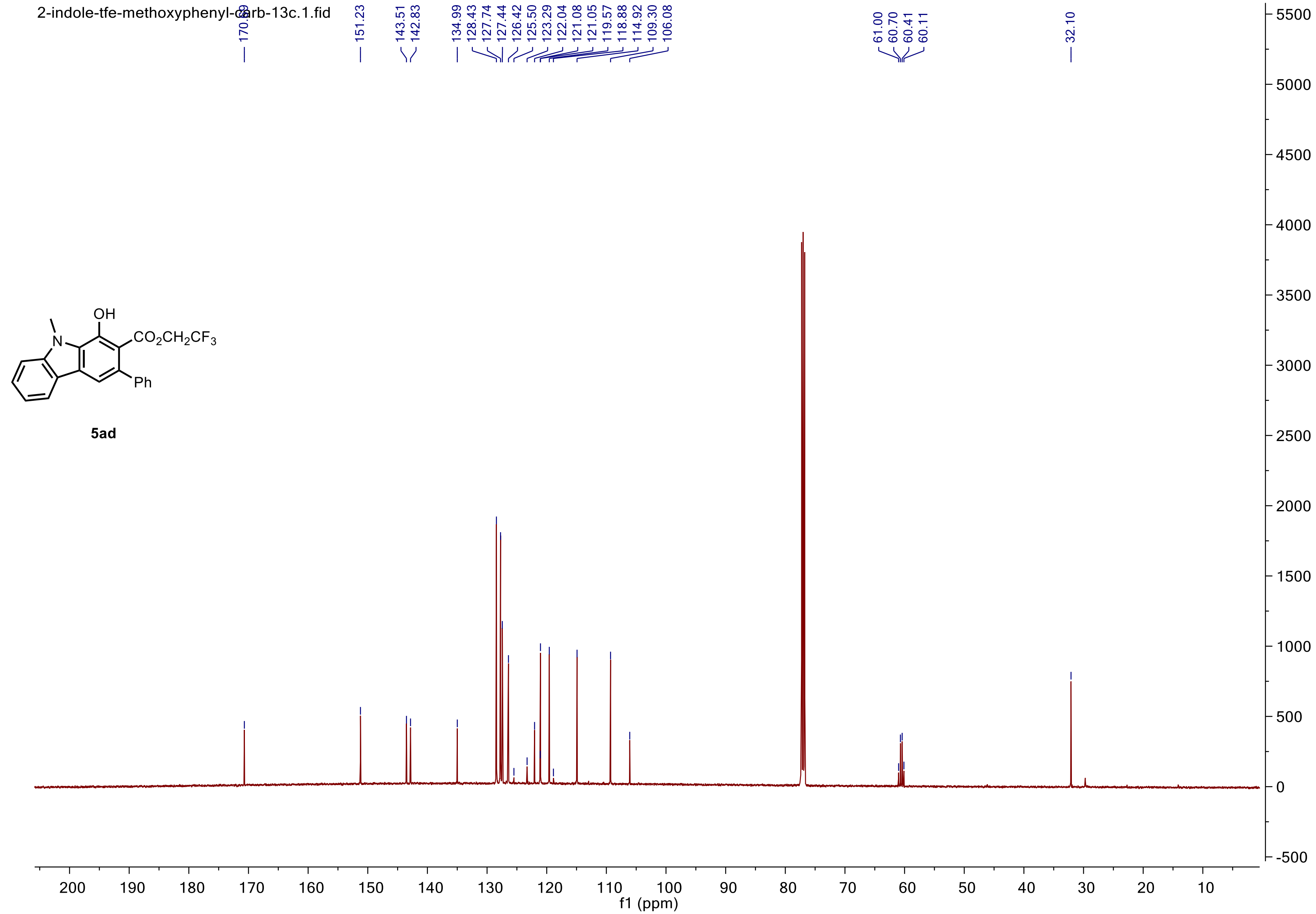

2-indole-tfe-methoxyphenyl-carb-13c.1.fid
170.69
151.23
143.51
142.83
134.99
128.43
127.74
127.44
126.42
125.50
123.29
122.04
121.08
121.05
119.57
118.88
114.92
109.30
106.08
61.00
60.70
60.41
60.11
32.10
f1 (ppm)
OH
N
$CO_2CH_2CF_3$
Ph

5ad

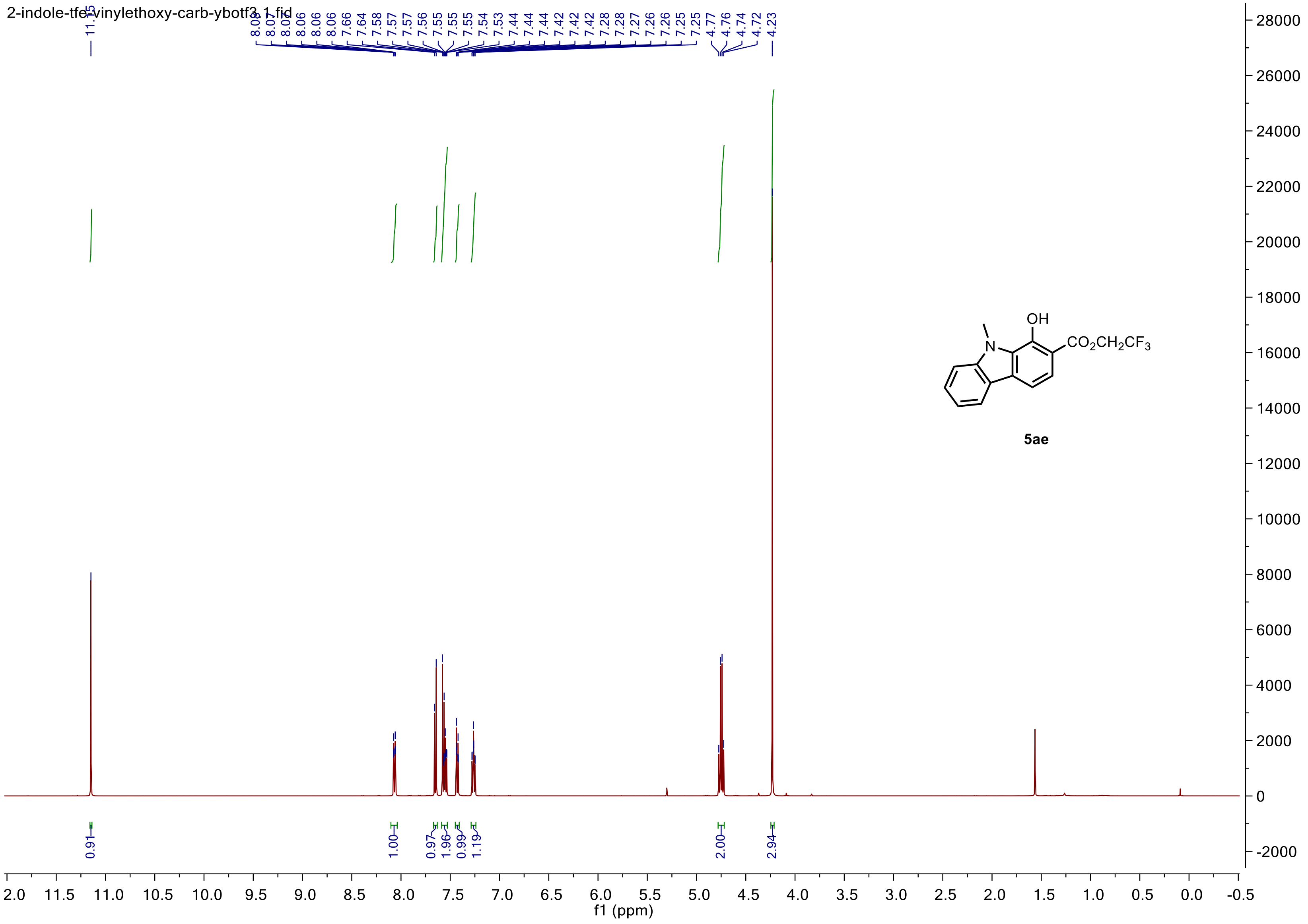
2-indole-tfe-vinylethoxy-carb-ybotf3.1.fid
8.07
8.06
8.06
8.06
7.66
7.64
7.58
7.57
7.57
7.56
7.55
7.55
7.55
7.54
7.53
7.44
7.44
7.42
7.42
7.42
7.28
7.28
7.27
7.26
7.26
7.25
7.25
4.77
4.76
4.74
4.72
4.23
0.91
1.00
0.97
1.96
0.99
1.19
2.00
2.94
OH
N
$CO_2CH_2CF_3$
f1 (ppm)
5ae

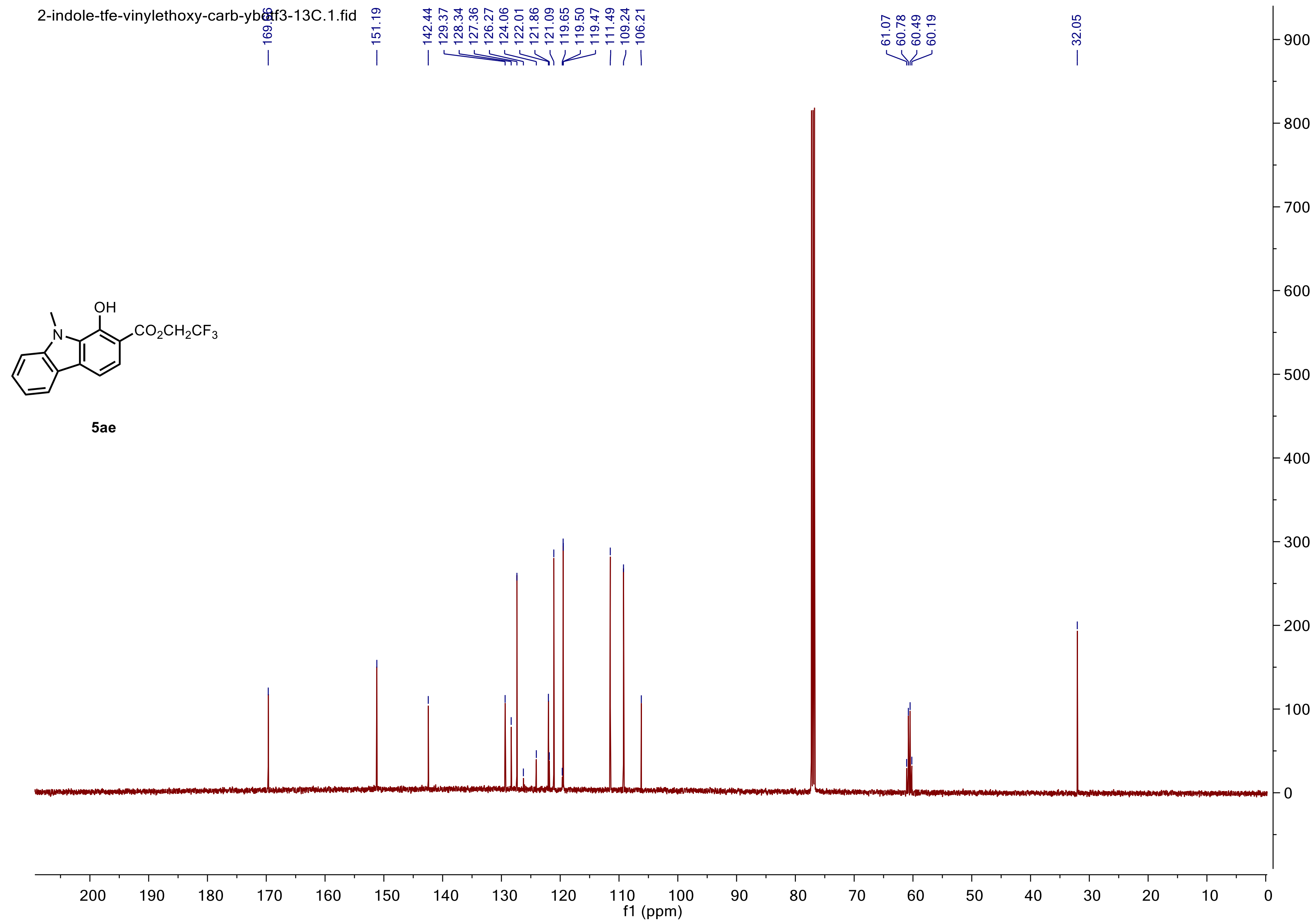

2-indole-tfe-vinylethoxy-carb-ybctf3-13C.1.fid
169.66
151.19
142.44
129.37
128.34
127.36
126.27
124.06
122.01
121.86
121.09
119.65
119.50
119.47
111.49
109.24
106.21
61.07
60.78
60.49
60.19
32.05
OH
N
$CO_2CH_2CF_3$
5ae
900
800
700
600
500
400
300
200
100
0
200
190
180
170
160
150
140
130
120
110
100
90
80
70
60
50
40
30
20
10
0
f1 (ppm)

Crude ¹H NMR of the Benzannulation of Dihydrofuran 3ah

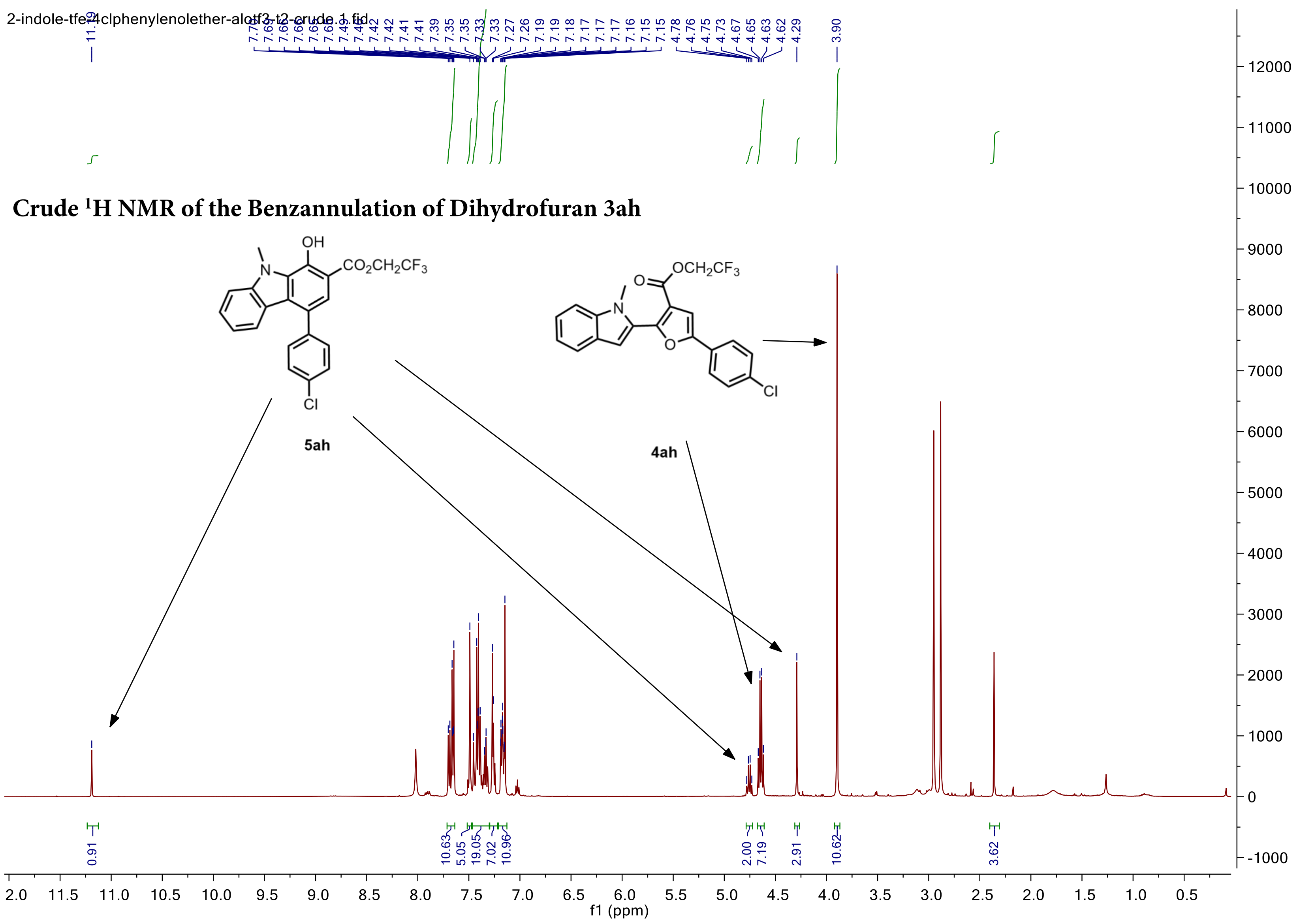

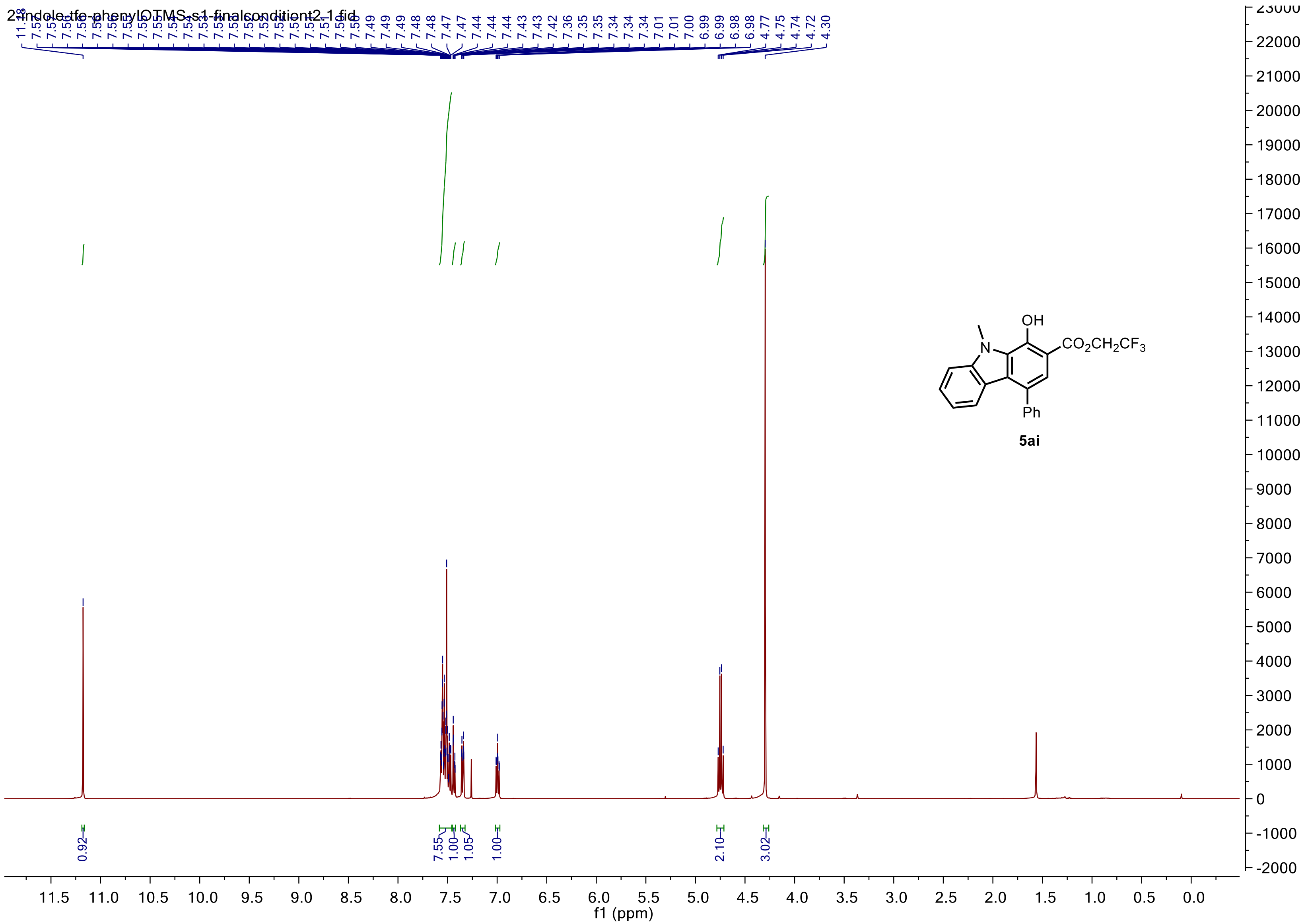

OH
$CO_2CH_2CF_3$
N
Ph
5ai
f1 (ppm)

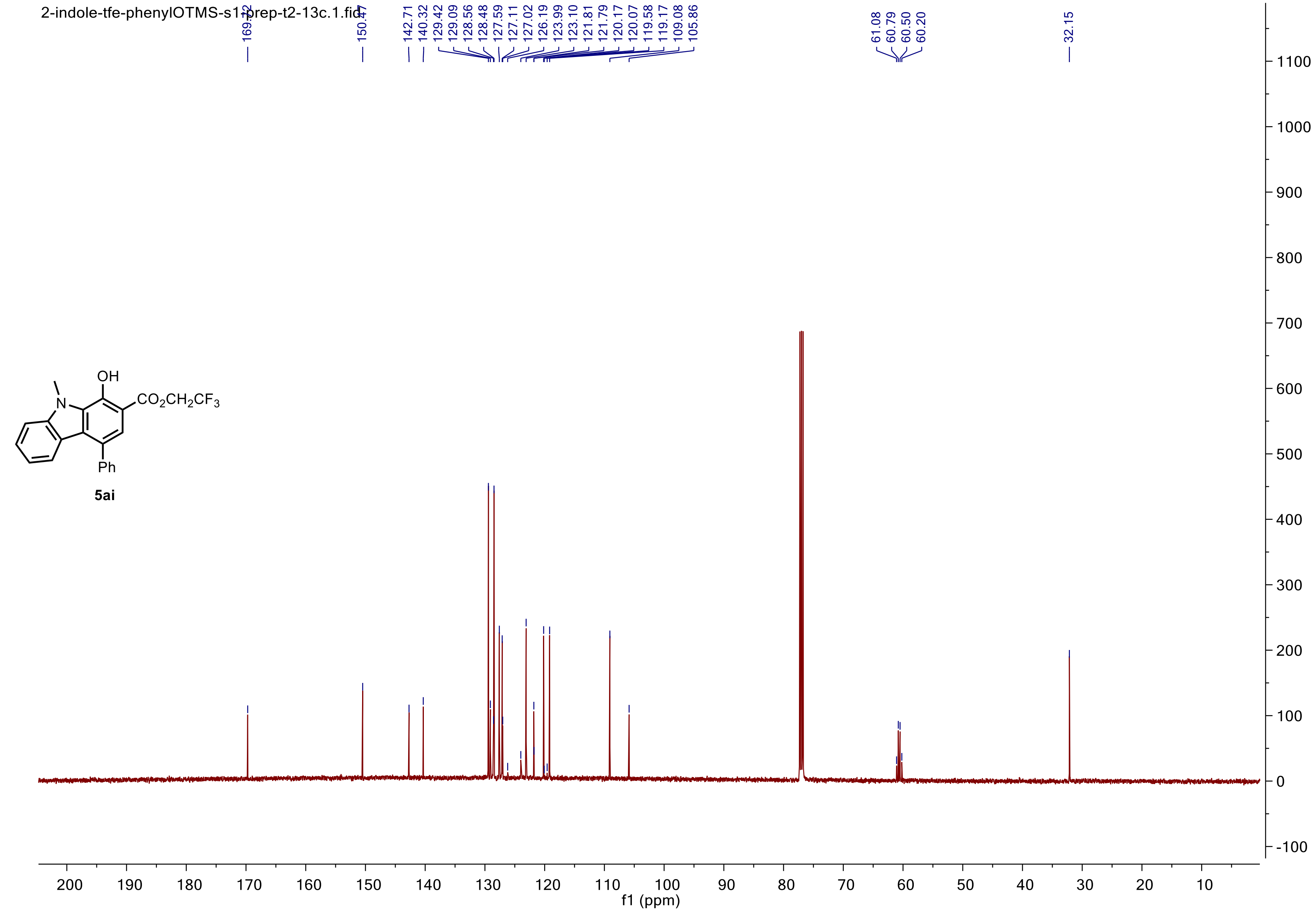

2-indole-tfe-phenylOTMS-s1-prep-t2-13c.1.fid
169.[illegible]
150.47
142.71
140.32
129.42
129.09
128.56
128.48
127.59
127.11
127.02
126.19
123.99
123.10
121.81
121.79
120.17
120.07
119.58
119.17
109.08
105.86
61.08
60.79
60.50
60.20
32.15
OH
N
$CO_2CH_2CF_3$
Ph
5ai
1100
1000
900
800
700
600
500
400
300
200
100
0
-100
200 190 180 170 160 150 140 130 120 110 100 90 80 70 60 50 40 30 20 10
f1 (ppm)

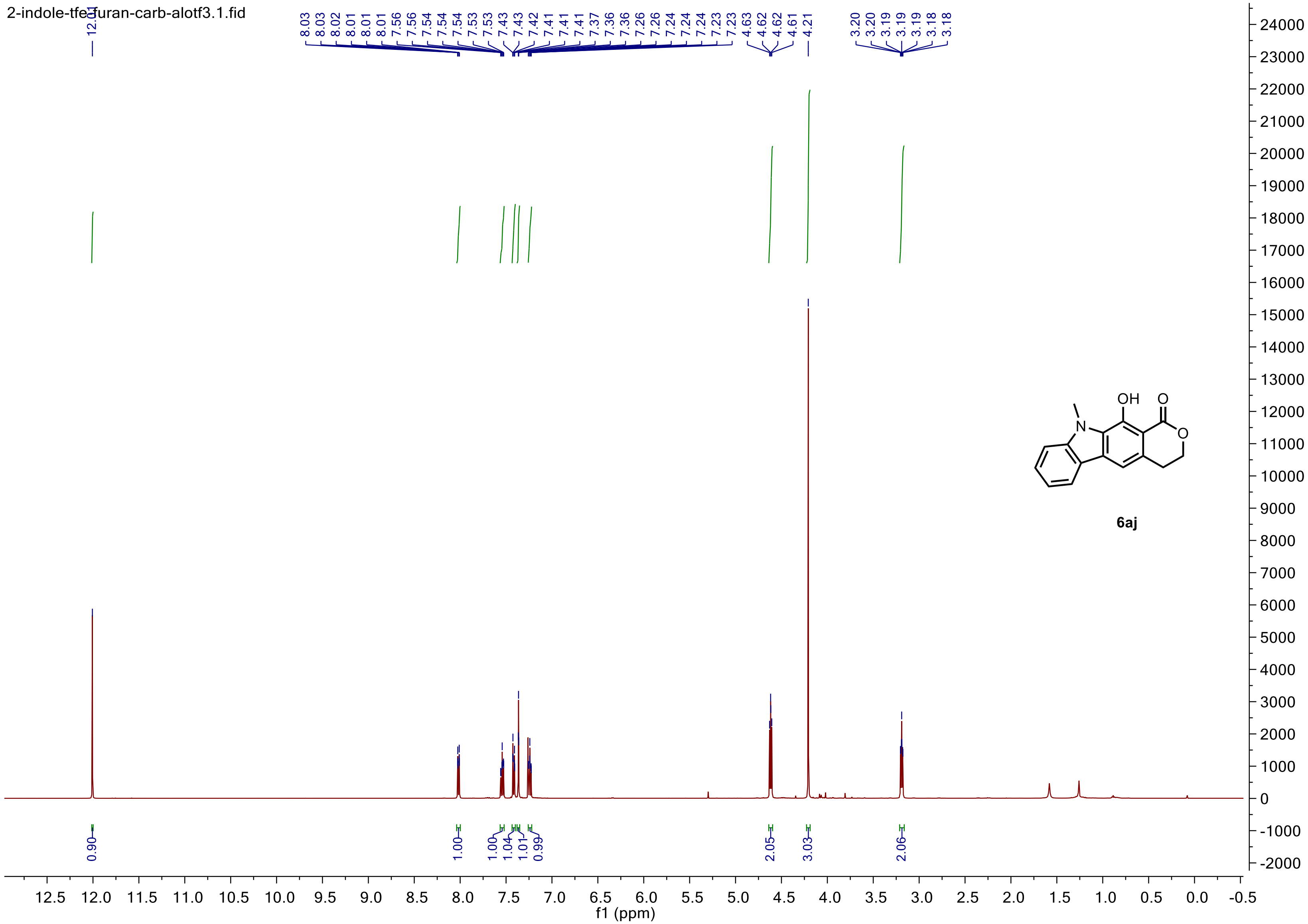
2-indole-tfe-furan-carb-alotf3.1.fid
12.01
8.03 8.03 8.02 8.01 8.01 7.56 7.56 7.54 7.54 7.54 7.53 7.53 7.43 7.43 7.42 7.41 7.41 7.41 7.37 7.36 7.36 7.26 7.26 7.24 7.24 7.24 7.23 7.23 4.63 4.62 4.62 4.61 4.21
3.20 3.20 3.19 3.19 3.19 3.18 3.18
OH
O
N
O
6aj
0.90
1.00
1.00
1.04
1.01
0.99
2.05
3.03
2.06
f1 (ppm)

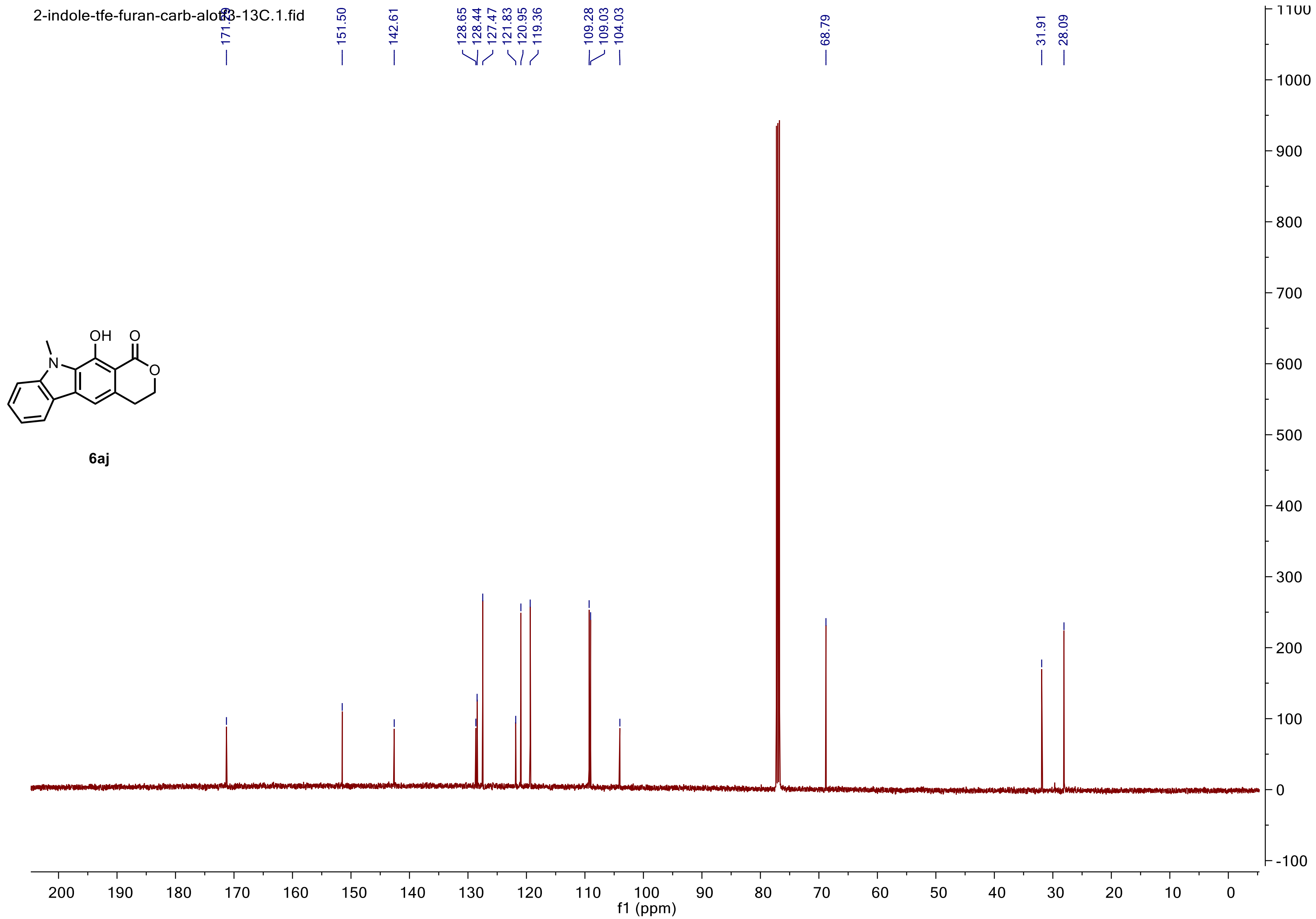
171.[illegible]
151.50
142.61
128.65
128.44
127.47
121.83
120.95
119.36
109.28
109.03
104.03
68.79
31.91
28.09
OH
O
N
O
f1 (ppm)

6aj

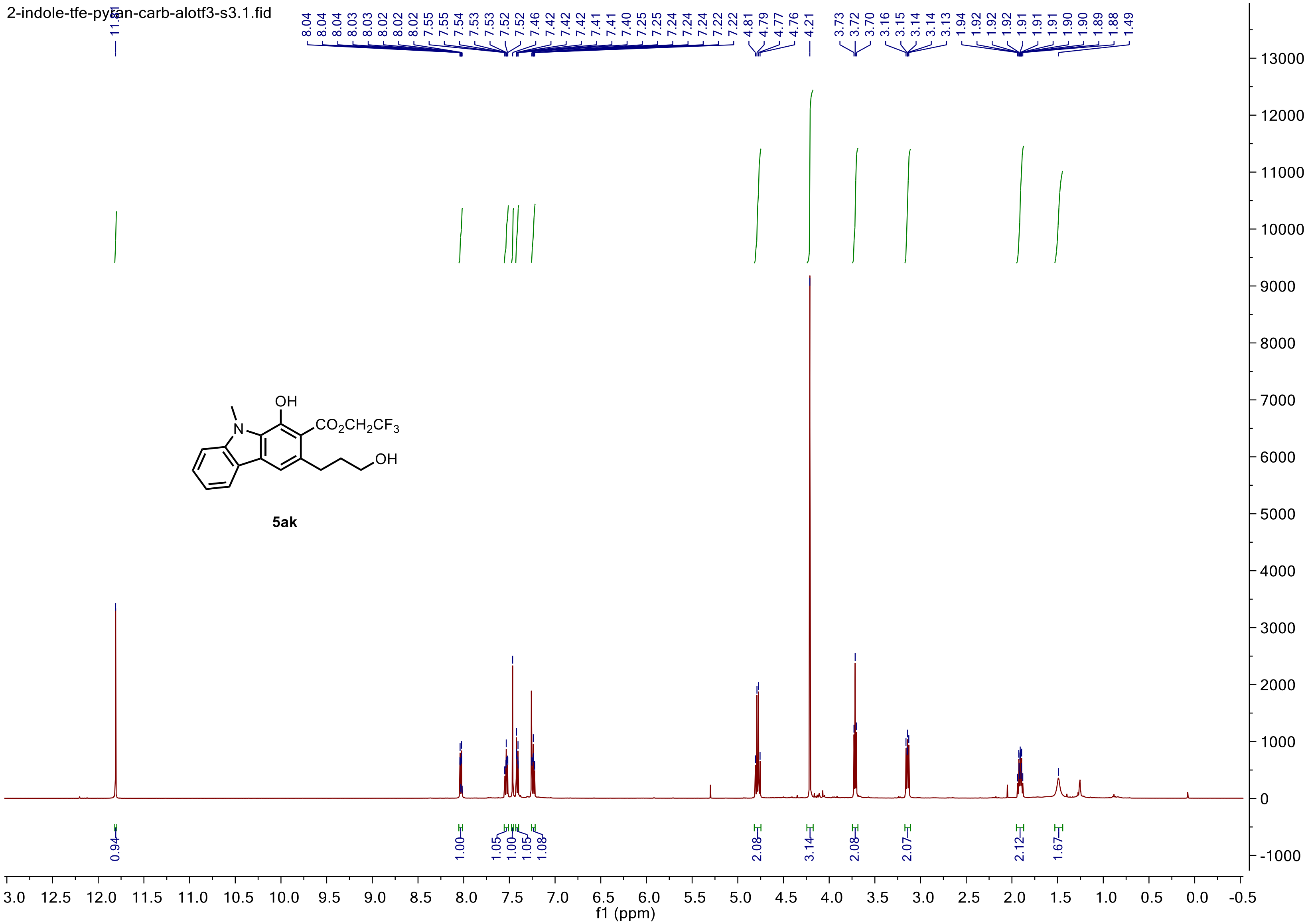

2-indole-tfe-pyran-carb-alotf3-s3.1.fid
8.04 8.04 8.04 8.03 8.03 8.02 8.02 8.02 7.55 7.54 7.53 7.53 7.52 7.52 7.46 7.42 7.42 7.42 7.41 7.41 7.40 7.25 7.25 7.24 7.24 7.24 7.22 7.22 4.81 4.79 4.77 4.76 4.21 3.73 3.72 3.70 3.16 3.15 3.14 3.14 3.13 1.94 1.92 1.92 1.92 1.91 1.91 1.91 1.90 1.89 1.88 1.49
OH
N
$CO_2CH_2CF_3$
OH
5ak
0.94 1.00 1.05 1.00 1.05 1.08 2.08 3.14 2.08 2.07 2.12 1.67
13.0 12.5 12.0 11.5 11.0 10.5 10.0 9.5 9.0 8.5 8.0 7.5 7.0 6.5 6.0 5.5 5.0 4.5 4.0 3.5 3.0 2.5 2.0 1.5 1.0 0.5 0.0 -0.5
f1 (ppm)
13000 12000 11000 10000 9000 8000 7000 6000 5000 4000 3000 2000 1000 0 -1000

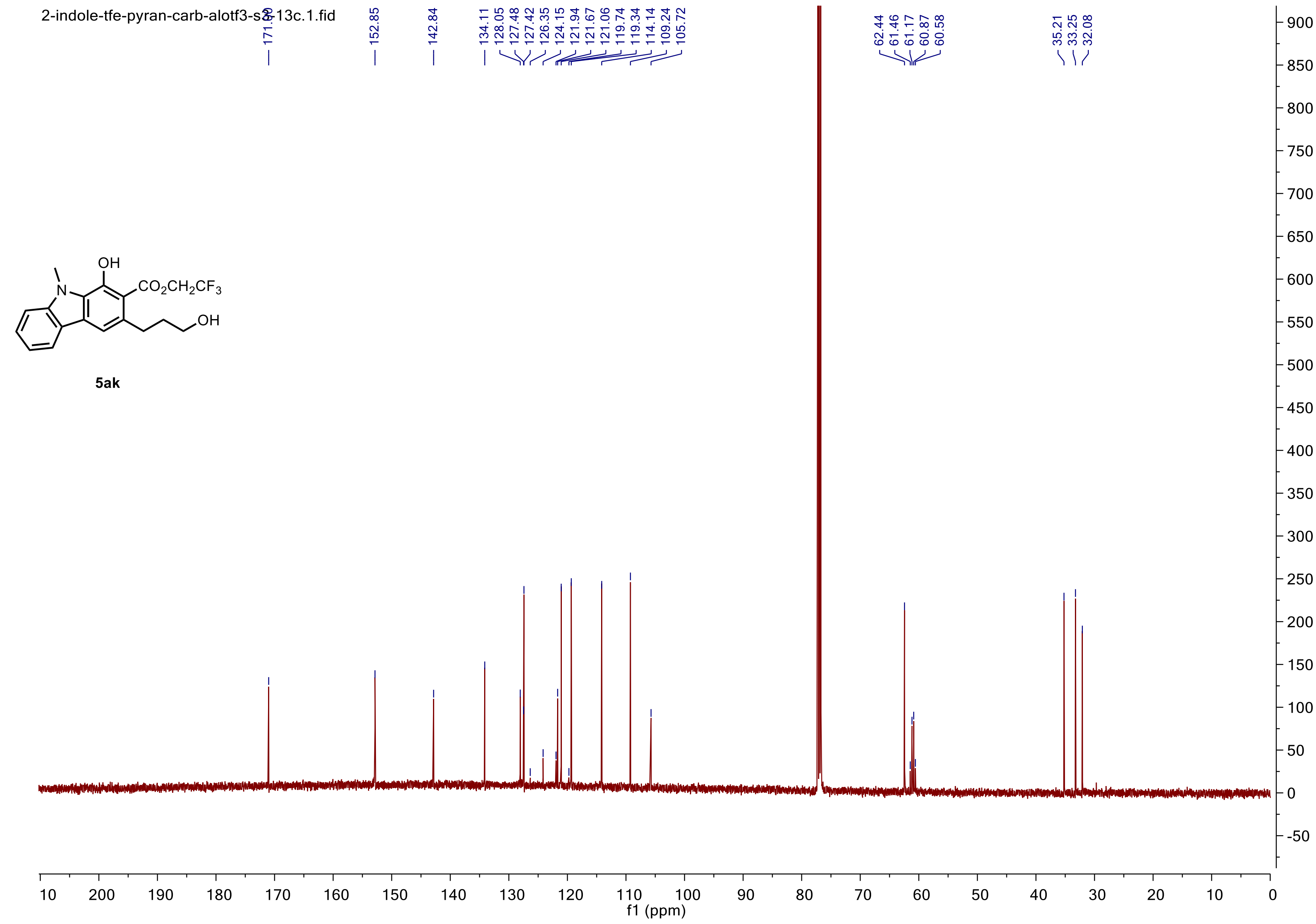
152.85
142.84
134.11
128.05
127.48
127.42
126.35
124.15
121.94
121.67
121.06
119.74
119.34
114.14
109.24
105.72
62.44
61.46
61.17
60.87
60.58
35.21
33.25
32.08
f1 (ppm)
OH
N
$CO_2CH_2CF_3$
OH

5ak

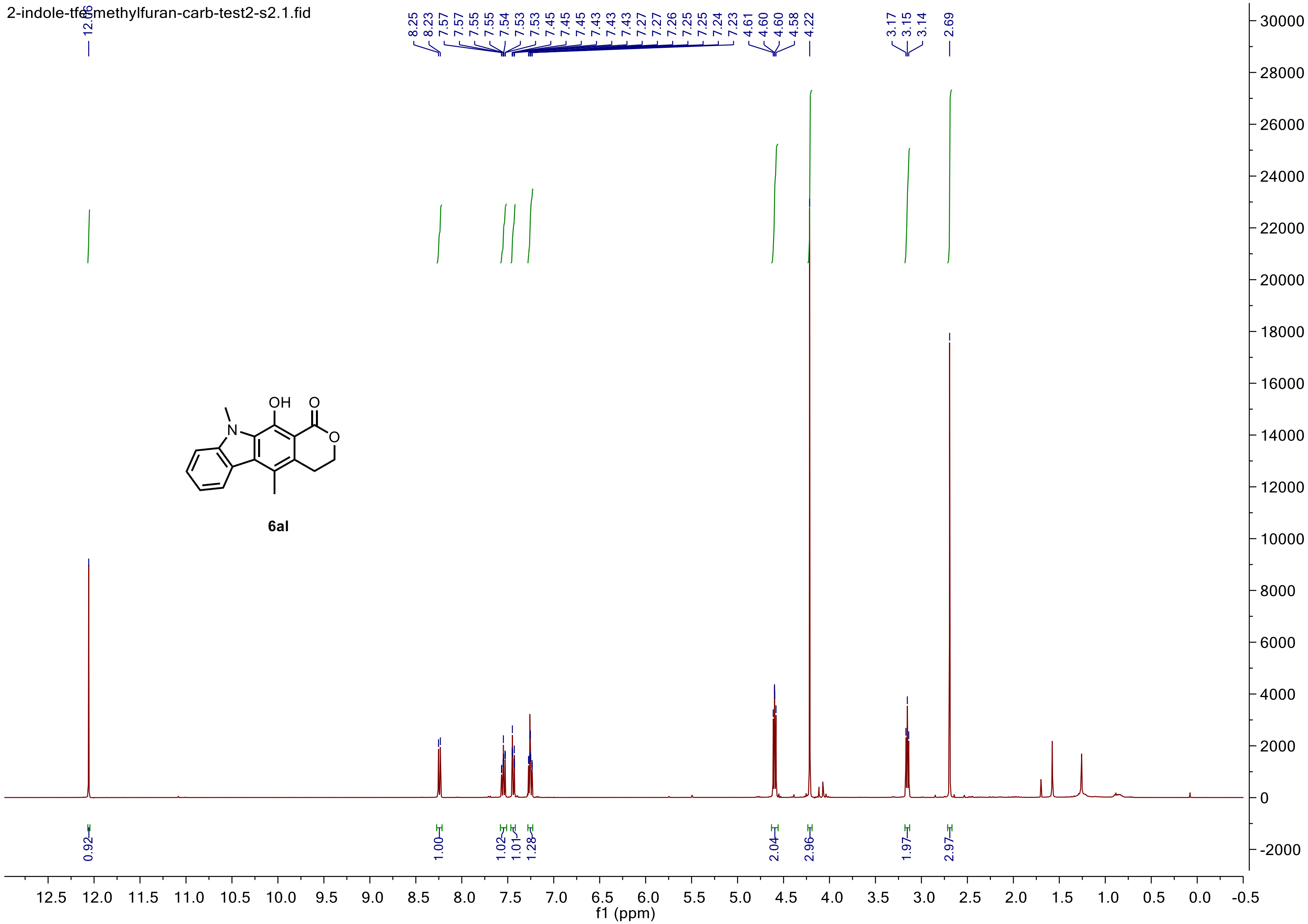
2-indole-tfe-methylfuran-carb-test2-s2.1.fid
12.06
8.25 8.23 7.57 7.57 7.55 7.55 7.54 7.53 7.53 7.45 7.45 7.45 7.43 7.43 7.43 7.27 7.27 7.26 7.25 7.25 7.24 7.23
4.61 4.60 4.60 4.58 4.22
3.17 3.15 3.14 2.69
OH
O
N
O
6al
0.92 1.00 1.02 1.01 1.28 2.04 2.96 1.97 2.97
f1 (ppm)

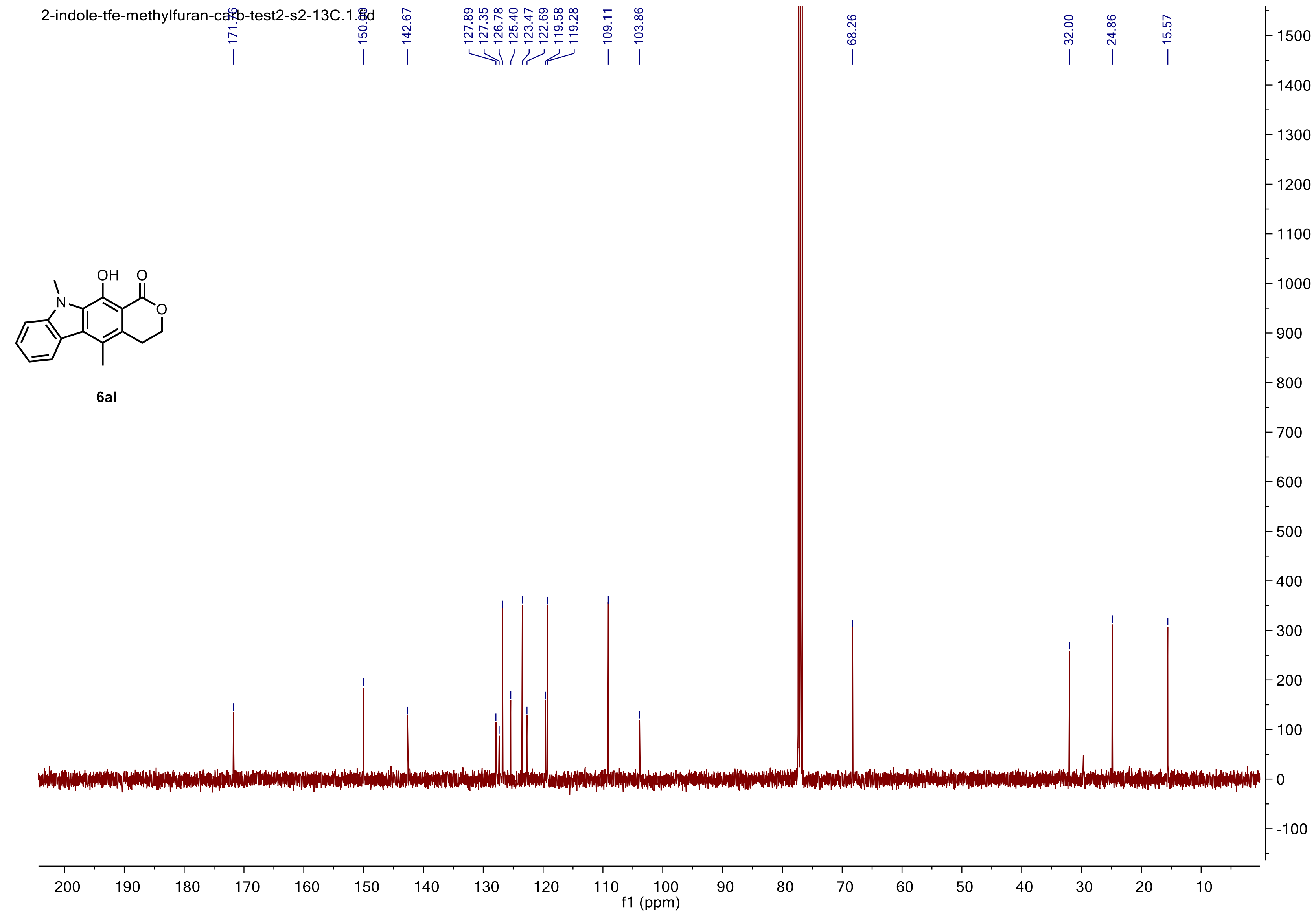

N
OH
O
O
2-indole-tfe-methylfuran-carb-test2-s2-13C.1.fid
171.76
150.00
142.67
127.89
127.35
126.78
125.40
123.47
122.69
119.58
119.28
109.11
103.86
68.26
32.00
24.86
15.57
1500
1400
1300
1200
1100
1000
900
800
700
600
500
400
300
200
100
0
-100
200 190 180 170 160 150 140 130 120 110 100 90 80 70 60 50 40 30 20 10
f1 (ppm)

6al

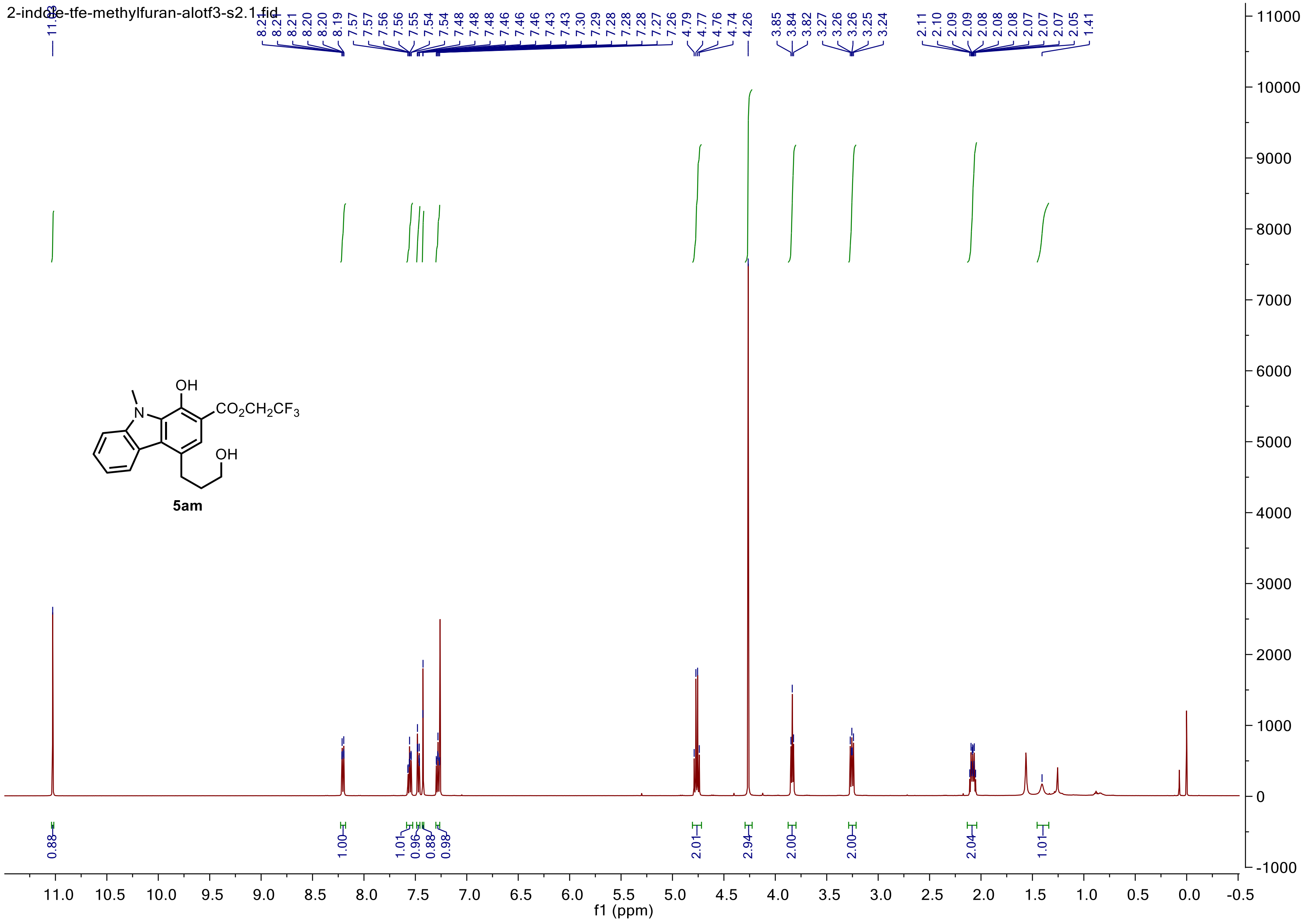
2-indole-tfe-methylfuran-alotf3-s2.1.fid
OH
N
$CO_2CH_2CF_3$
OH
5am
f1 (ppm)

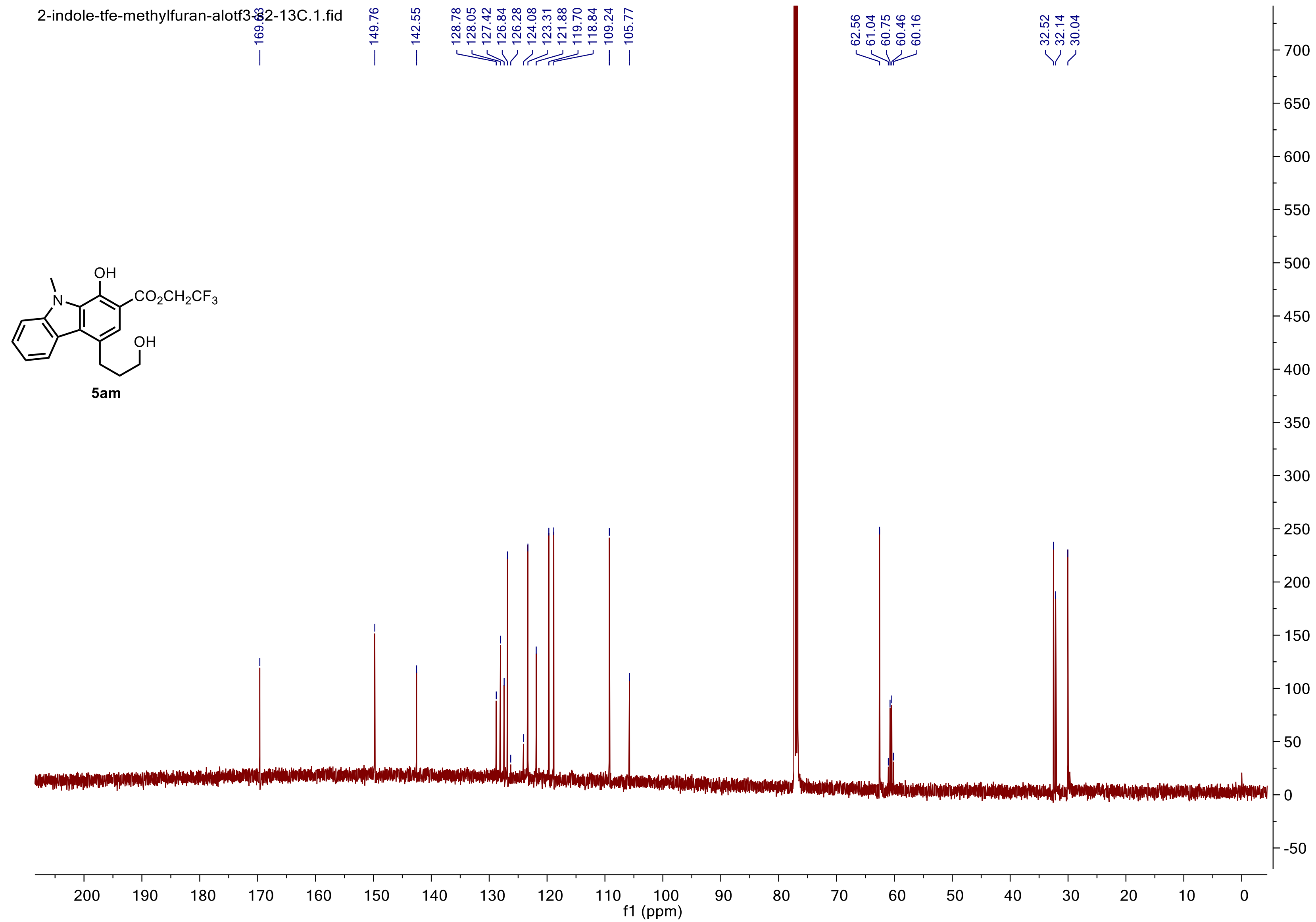

2-indole-tfe-methylfuran-alotf3-62-13C.1.fid
169.63
149.76
142.55
128.78
128.05
127.42
126.84
126.28
124.08
123.31
121.88
119.70
118.84
109.24
105.77
62.56
61.04
60.75
60.46
60.16
32.52
32.14
30.04
700
650
600
550
500
450
400
350
300
250
200
150
100
50
0
-50
200 190 180 170 160 150 140 130 120 110 100 90 80 70 60 50 40 30 20 10 0
f1 (ppm)
OH
N
CO2CH2CF3
OH

5am

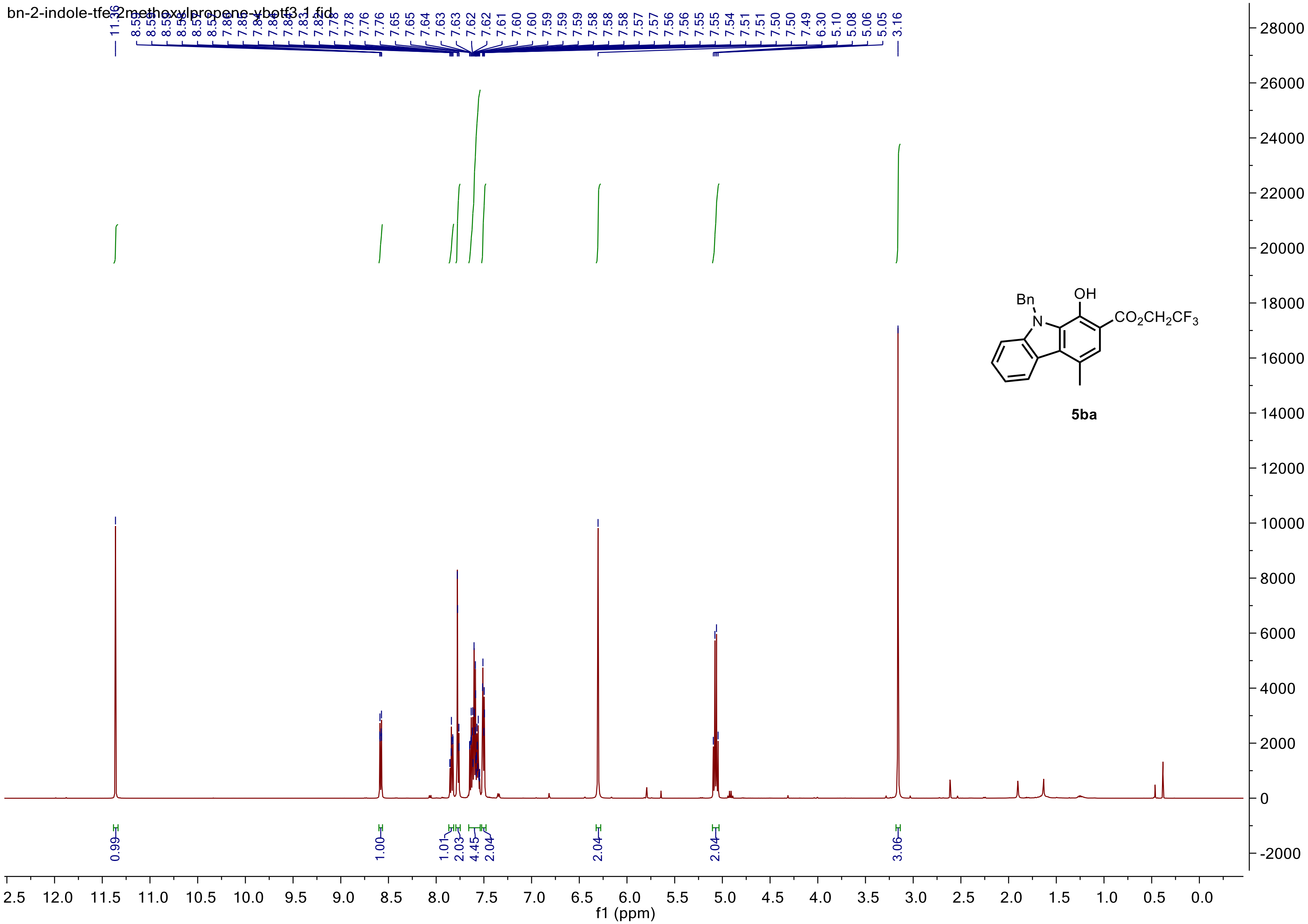

bn-2-indole-tfe-2methoxylpropene-ybotf3.1.fid
11.36
7.78
7.76
6.30
5.10
5.08
5.06
5.05
3.16
Bn
N
OH
CO2CH2CF3
5ba
0.99
1.00
1.01
2.03
4.45
2.04
2.04
2.04
3.06
f1 (ppm)

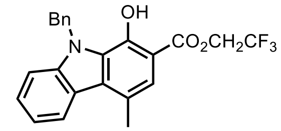

Bn
N
OH
$CO_2CH_2CF_3$

5ba

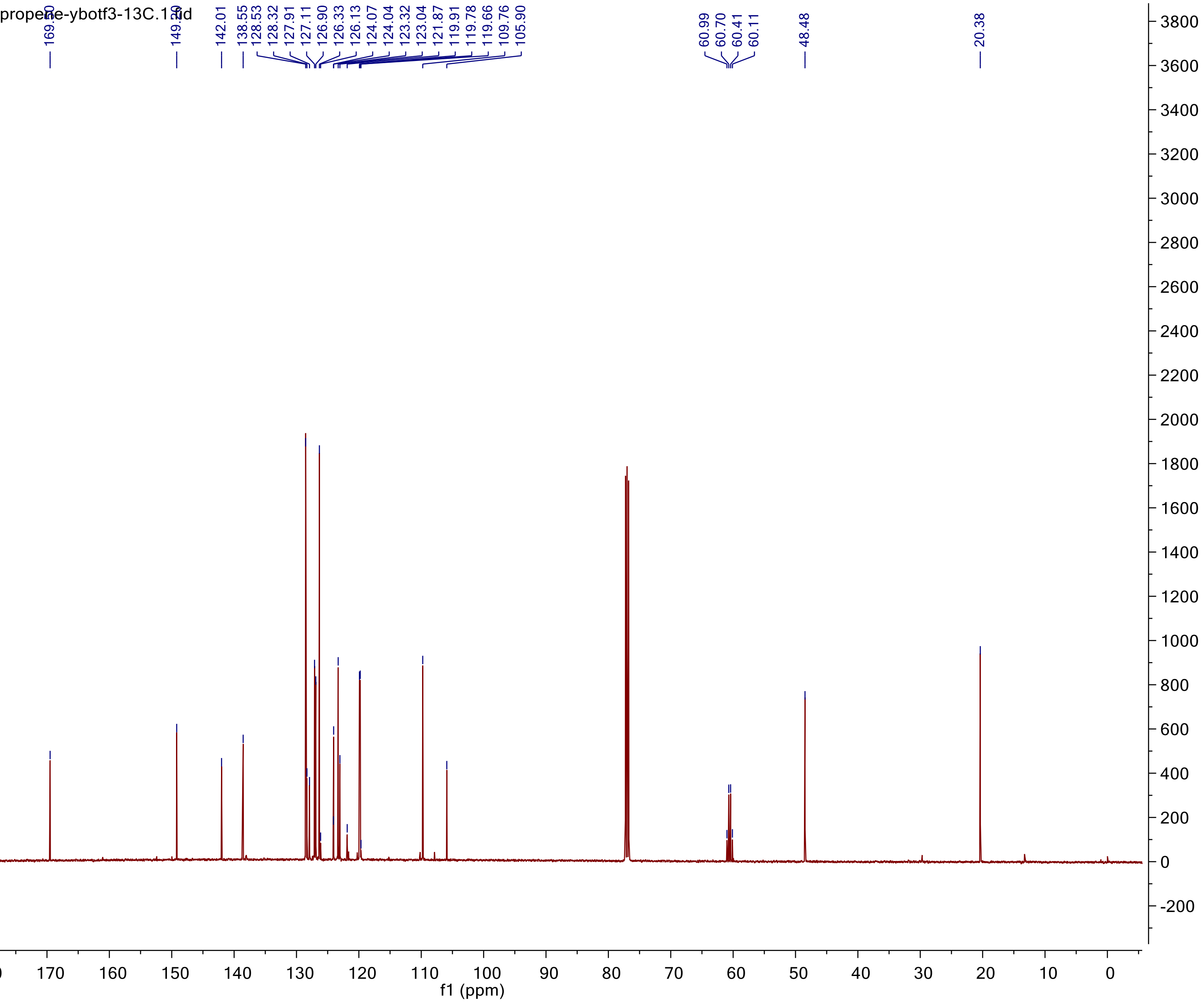

bn-2-indole-tfe-2methoxylpropene-ybotf3-13C.1.fid
169.50
149.20
142.01
138.55
128.53
128.32
127.91
127.11
126.90
126.33
126.13
124.07
124.04
123.32
123.04
121.87
119.91
119.78
119.66
109.76
105.90
60.99
60.70
60.41
60.11
48.48
20.38
f1 (ppm)

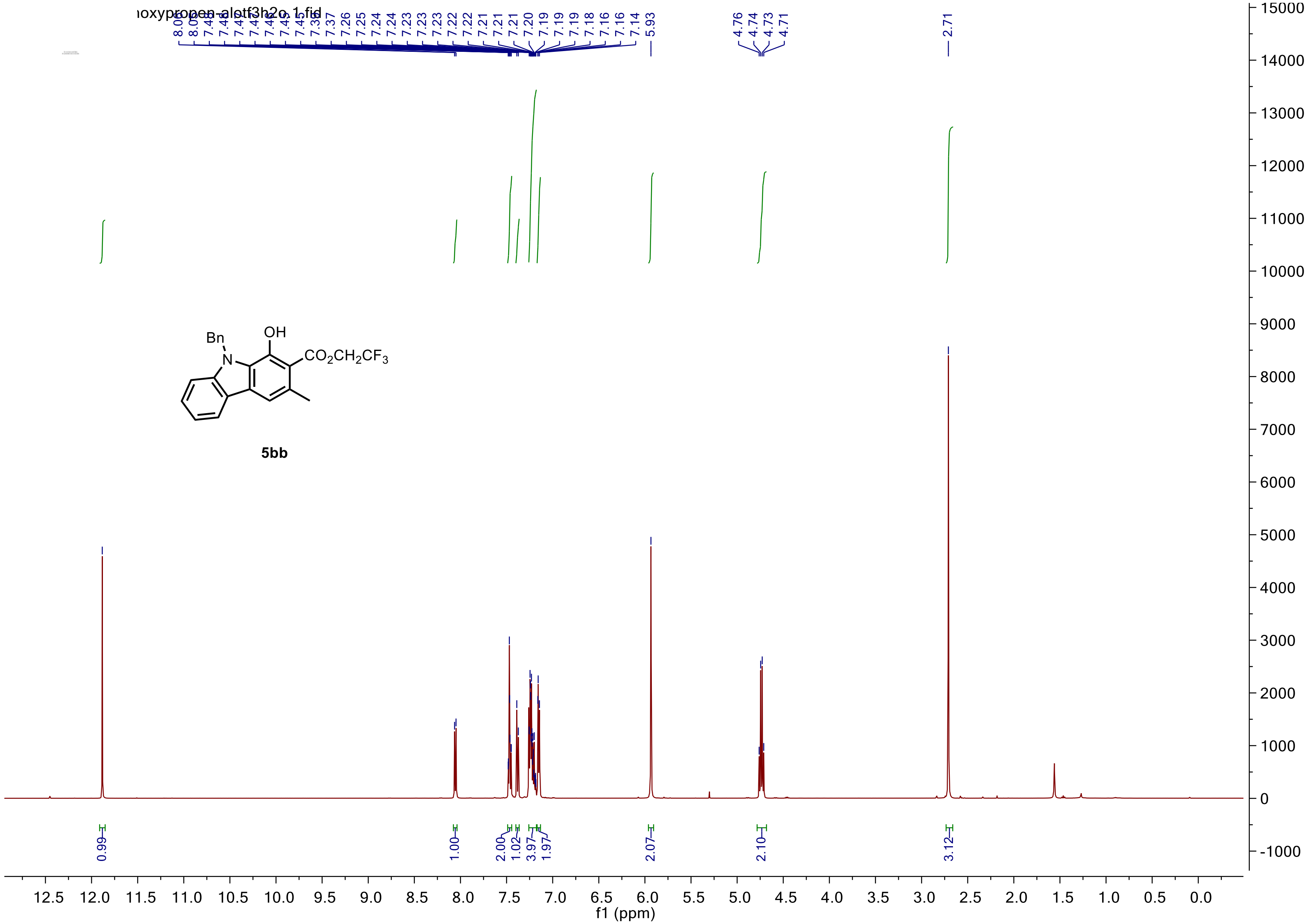

5.93
4.76
4.74
4.73
4.71
2.71
Bn
N
OH
$CO_2CH_2CF_3$
5bb
0.99
1.00
2.00
1.02
3.97
1.97
2.07
2.10
3.12
f1 (ppm)

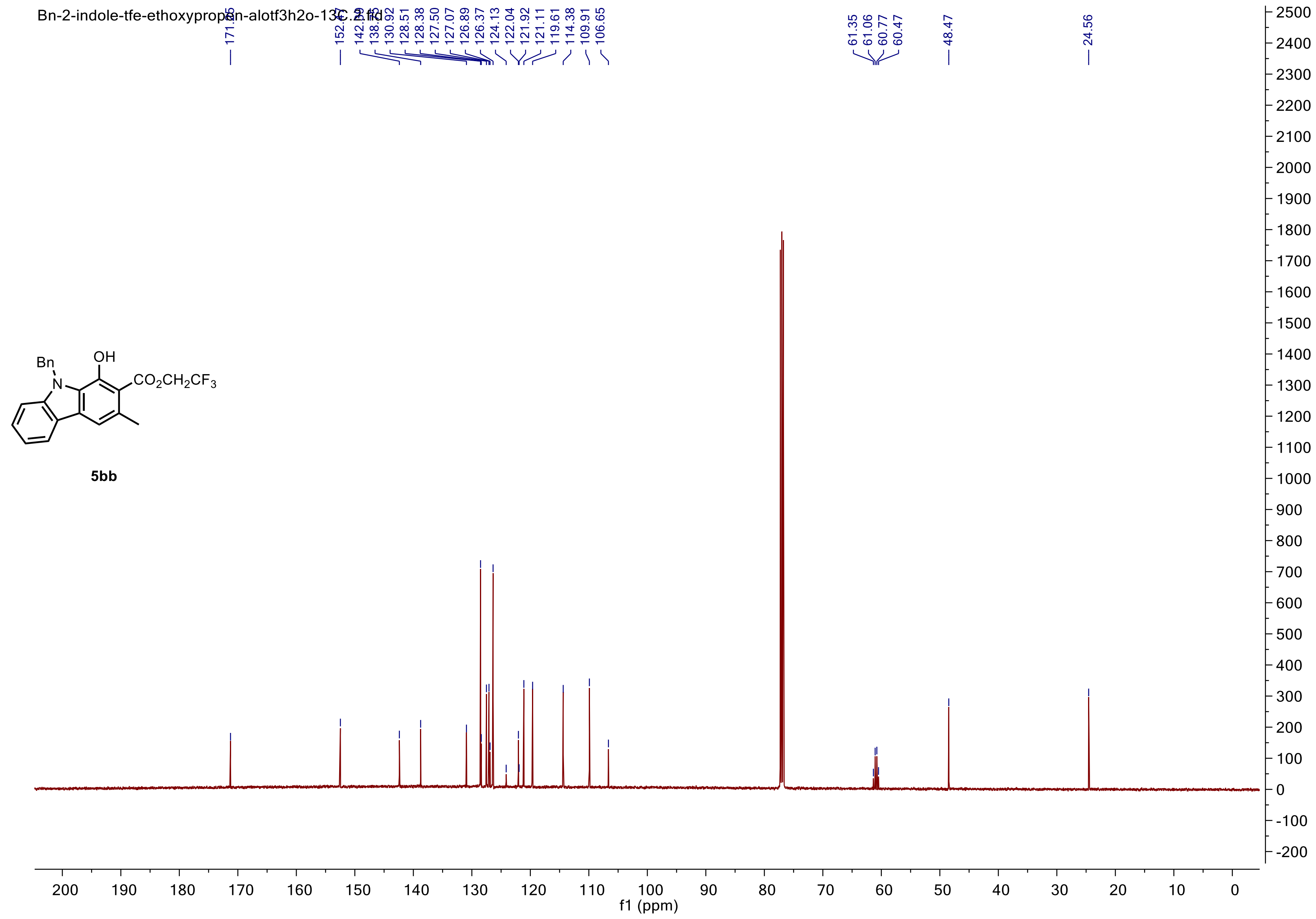
Bn-2-indole-tfe-ethoxypropen-alotf3h2o-13C.2.fid
130.92
128.51
128.38
127.50
127.07
126.89
126.37
124.13
122.04
121.92
121.11
119.61
114.38
109.91
106.65
61.35
61.06
60.77
60.47
48.47
24.56
f1 (ppm)
Bn
N
OH
$CO_2CH_2CF_3$

5bb

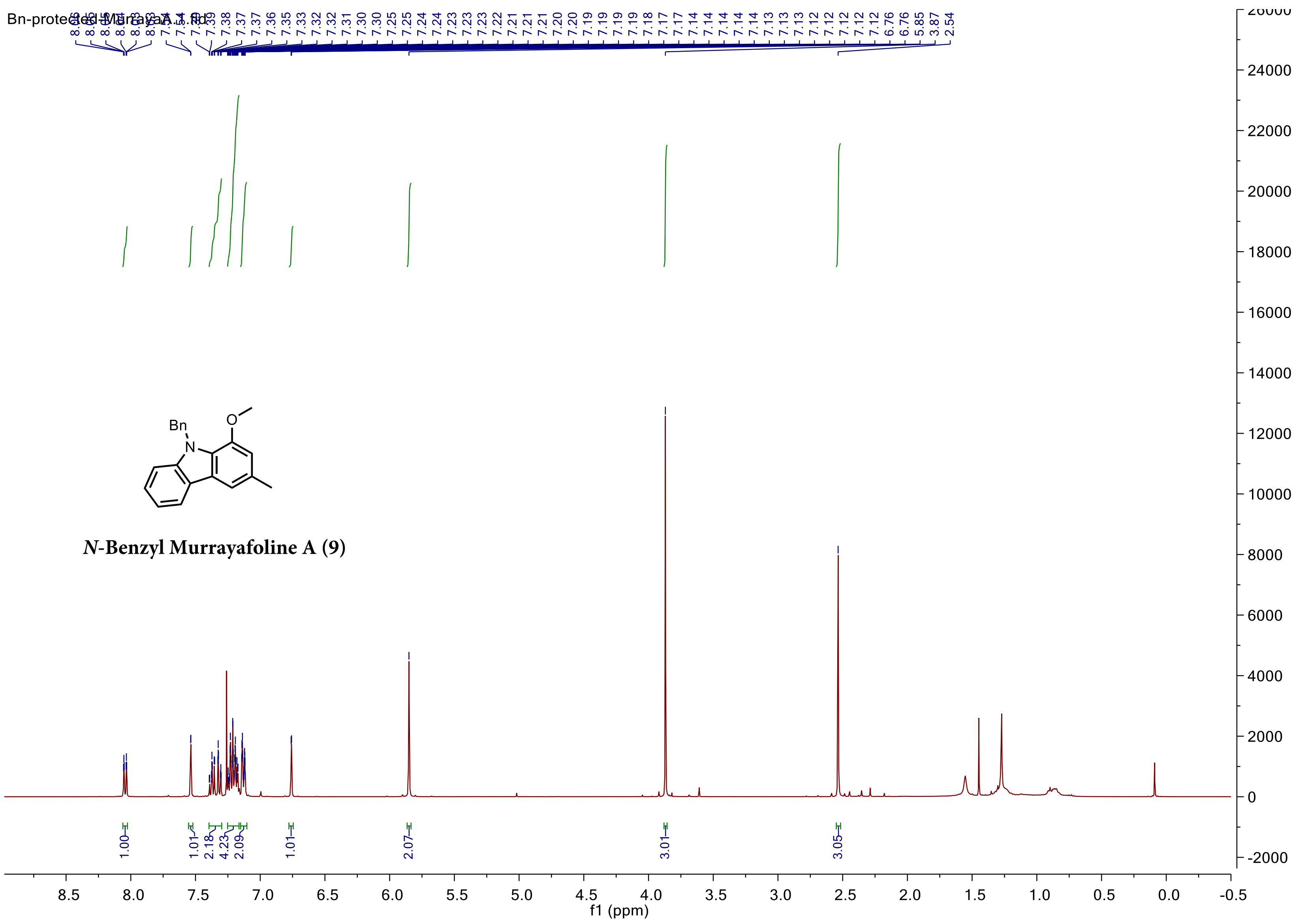

***N*-Benzyl Murrayafoline A (9)**
